# Development of Small-Molecule Allosteric Modulators of Beta-Galactosidase (β-Gal) for the Treatment of GM1 Gangliosidosis and Morquio B

**DOI:** 10.3390/ijms27083631

**Published:** 2026-04-18

**Authors:** Natàlia Pérez-Carmona, Elena Cubero, Ana Ruano, Maria Pons-Vizcarra, Aida Delgado, Ana Trapero, Marc Reves, Laura Rodríguez-Pascau, Joanne Taylor, Marc Martinell, Xavier Barril, Ana María García-Collazo

**Affiliations:** 1 Gain Therapeutics Sucursal en España, Parc Científic de Barcelona, 08028 Barcelona, Spainecubero@gaintherapeutics.com (E.C.); aruano@gaintherapeutics.com (A.R.); mpons@gaintherapeutics.com (M.P.-V.); adelgado@gaintherapeutics.com (A.D.);; 2 Minoryx Therapeutics, 08302 Barcelona, Spainlrodriguez@minoryx.com (L.R.-P.); mmartinell@minoryx.com (M.M.); 3 Gain Therapeutics, Inc., Bethesda, MD 20814, USA; jtaylor@gaintherapeutics.com; 4 Facultat de Farmàcia, IBUB & IQTC, Universitat de Barcelona, 08028 Barcelona, Spain; 5 Catalan Institution for Research and Advanced Studies (ICREA), 08010 Barcelona, Spain

**Keywords:** beta-galactosidase, *GLB1*, GM1-gangliosidosis, pharmacological chaperone, lysosomal storage disorders, protein misfolding, substrate depletion, structurally targeted allosteric regulators (STARs), site-directed enzyme enhancement therapy (SEE-Tx), Magellan platform

## Abstract

GM1 gangliosidosis and Morquio B are rare lysosomal storage disorders (LSDs) with significant unmet medical needs. These disorders result from mutations in the galactosidase beta 1 (*GLB1*) gene, leading to impaired β-galactosidase (β-Gal) activity and toxic substrate accumulation. The lack of approved disease-modifying therapies for GM1 gangliosidosis and Morquio B, along with the challenges of achieving effective central nervous system delivery, has driven interest in small-molecule pharmacological chaperones (PCs) to restore β-Gal stability and function. Using Gain Therapeutics’ Magellan™ platform, a novel allosteric binding site on β-Gal was identified, enabling the discovery of a new class of Structurally Targeted Allosteric Regulators (STARs). Medicinal chemistry optimization produced a structurally unique STAR compound series, demonstrating broad β-Gal stabilizing effects. The therapeutic potential of these compounds was evaluated in vitro using a canine fibroblast model of GM1 gangliosidosis, where they were shown to significantly reduce toxic GM1 ganglioside accumulation. Immunocytochemistry-based assays confirmed substrate clearance and provided reliable structure–activity relationships, guiding further compound development. Notably, STARs achieved greater substrate clearance than the competitive PC N-nonyl-deoxygalactonojirimycin (NN-DGJ) under the conditions tested, as demonstrated by immunocytochemistry-based assays. While these findings are encouraging, further in vivo studies are required to validate the therapeutic efficacy of these few STAR compounds, particularly in addressing the neurodegenerative aspects of GM1 gangliosidosis. This study underscores the potential of the Magellan platform in identifying STAR molecules and provides a strong foundation for further optimization and preclinical validation in *GLB1*-related disorders, particularly GM1 gangliosidosis.

## 1. Introduction

β-Galactosidase (β-Gal) is a lysosomal enzyme that plays a critical role in the degradation of complex molecules by removing terminal β-galactose residues from various substrates, including gangliosides, oligosaccharides with terminal β-linked galactose, and glycosaminoglycans [1]. Mutations in the galactosidase beta 1 (*GLB1*) gene, which encodes β-Gal, result in reduced or absent enzymatic activity. This deficiency leads to the progressive and toxic accumulation of β-Gal substrates, such as GM1 ganglioside or keratan sulfate, within lysosomes. The buildup leads to cellular dysfunction and contributes to the development of two distinct lysosomal storage disorders (LSDs): GM1 gangliosidosis and Morquio B disease (mucopolysaccharidosis type IVB), respectively [1,2]. GM1 gangliosidosis is characterized by severe neurodegenerative symptoms due to the accumulation of GM1 ganglioside in the central nervous system (CNS). Both conditions are extremely rare. GM1 gangliosidosis has an estimated incidence of approximately 1 in 100,000 to 200,000 live births worldwide [1,3,4,5], while Morquio B disease is even rarer, with a reported prevalence of 1 in 250,000 to 1,000,000 live births [4,5].

Currently, there is no cure for either Morquio B disease or GM1 gangliosidosis. Research efforts aimed at treating LSDs have explored a range of therapeutic strategies, including substrate reduction therapy (SRT), stem cell transplantation, enzyme replacement therapy (ERT), and gene therapy [6]. These approaches aim to delay disease progression, improve patient quality of life, and prolong survival by either reducing substrate accumulation or introducing functional β-Gal through exogenous delivery or gene-based methods. Another promising strategy involves the use of small-molecule pharmacological chaperones (PCs) [7]. Unlike ERT and SRT, PC therapy not only targets substrate accumulation but also addresses protein folding defects, which may contribute to the underlying pathology of these disorders. Additionally, small-molecule PCs have the unique advantage of crossing the blood–brain barrier (BBB), making them particularly relevant for treating the neurodegenerative aspects of GM1 gangliosidosis. While many PCs act as competitive inhibitors by binding to the enzyme’s active site, recent efforts have focused on developing non-competitive, allosteric PCs that stabilize the enzyme without inhibiting its activity, offering a promising therapeutic approach for β-Gal-related disorders [8,9,10,11,12,13,14].

Previous PC approaches for *GLB1*-related disorders have primarily focused on competitive inhibitory chaperones, including iminosugars and other small molecules. Competitive inhibitors bind to the active site of β-Gal, stabilizing the enzyme and preventing its degradation in the endoplasmic reticulum, thereby increasing the amount of functional enzyme that reaches the lysosome. For example, the iminosugar N-nonyl-deoxygalactonojirimycin (NN-DGJ) has been shown to reduce GM1 ganglioside storage in cellular and animal models of GM1 gangliosidosis [8,15]. Matsuda et al. (2003) reported that N-octyl-DGJ improved β-Gal activity in fibroblasts derived from GM1 gangliosidosis patients, highlighting its potential as a PC [15]. Similarly, other iminosugar derivatives, such as N-octyl- and N-butyl-DGJ, have demonstrated efficacy in stabilizing mutant β-Gal and reducing substrate accumulation in vitro [16,17]. Rigat et al. (2012) further explored the structure–activity relationships of iminosugars, identifying modifications that enhanced their stabilizing effects on β-Gal in a feline GM1 gangliosidosis model [16].

Other small molecules have also been investigated as PCs for *GLB1*-related disorders. Clemente et al. (2022) described the development of novel iminosugar-based compounds, specifically trihydroxypiperidine derivatives, that stabilize β-Gal and reduce GM1 ganglioside accumulation in cellular models [18]. Siriwardena et al. (2014) identified a series of 1,5-dideoxy-1,5-iminoribitol C-glycosides with PC activity, demonstrating their ability to enhance mutant β-Gal stability and function in GM1 gangliosidosis patient fibroblasts, with one compound achieving up to a 6-fold increase in enzymatic activity [19]. These studies collectively underscore the potential of PCs to address the enzymatic dysfunction underlying GM1 gangliosidosis and Morquio B disease. However, many competitive inhibitors face limitations, including their potential to interfere with substrate binding and enzymatic activity, as well as challenges in achieving effective CNS delivery [7]. Stütz et al. (2021) provided a comprehensive review of the field, summarizing the progress and challenges associated with PC development for *GLB1*-related disorders [7].

The Magellan^TM^ innovative drug discovery platform (formerly SEE-Tx^®^, Site-directed Enzyme Enhancement Therapy) has facilitated the discovery of small molecules known as structurally targeted allosteric regulators (STARs), which can function as allosteric PCs [20,21]. This physics-based screening technology leverages three-dimensional protein structures, advanced supercomputing methods, and virtual screening to identify druggable allosteric sites and small molecules that bind to them, enabling the selection of potential PCs that can be further validated.

In this study, we build on these prior approaches by applying the Magellan platform to identify novel allosteric, druggable binding sites and discover STARs of β-Gal with potential therapeutic relevance. Unlike competitive inhibitory chaperones, STAR compounds are designed to bind to allosteric sites, stabilizing the enzyme without interfering with its active site or substrate binding. By combining advanced computational screening with medicinal chemistry strategies, we aimed to develop and optimize small molecules capable of stabilizing the β-Gal enzyme and enhancing its activity. To assess the therapeutic potential of these compounds, we employed a cell-based fluorescent imaging assay to evaluate their ability to reduce GM1 ganglioside accumulation in a cellular model of GM1 gangliosidosis. While the findings highlight the promise of STAR compounds as stabilizing PCs, further kinetic and mechanistic characterization is required to fully understand their mode of action. This study offers a novel approach to potentially address the underlying enzymatic dysfunction in LSDs.

## 2. Results

### 2.1. Allosteric Binding Site Selection Using Magellan Technology

The Magellan drug discovery platform was used to identify a novel druggable allosteric cavity, distinct from the active site, within the high-resolution structure of native human β-Gal (PDB ID: 3THC) [22]. Preliminary findings were briefly reported by Rudinskiy et al. (2023) [23]. The druggability of this newly identified pocket was evaluated using the MDmix v0.1 method [23,24,25,26,27], which combines molecular dynamics simulations in organic–aqueous solvent mixtures to reveal preferential solvent interaction sites. This analysis confirmed the presence of stable binding hotspots suitable for small-molecule interactions, validating the cavity for subsequent virtual screening using rDock v. 213.1 [28].

### 2.2. Discovery and Experimental Validation of Small-Molecule Hits That Stabilize β-Gal

#### 2.2.1. In Silico Identification of Small-Molecule Hits

A computational workflow screened 4,840,400 structurally unique compounds available from major commercial providers. The virtual screening applied cavity constraints, pharmacophoric restraints derived from MDmix hot spots, and an intermolecular docking score threshold of <−18.0 units, resulting in the selection of 9133 molecules. These compounds were docked into the binding site, ranked by normalized docking score (intermolecular energy divided by the number of non-hydrogen atoms), and clustered based on chemical similarity using Molecular ACCess System (MACCS) fingerprints, a set of 166 predefined structural keys commonly employed to encode the presence or absence of specific substructures in small molecules [29]. Representative compounds from diverse clusters were visually inspected to identify virtual hits for testing.

**Figure 1:**
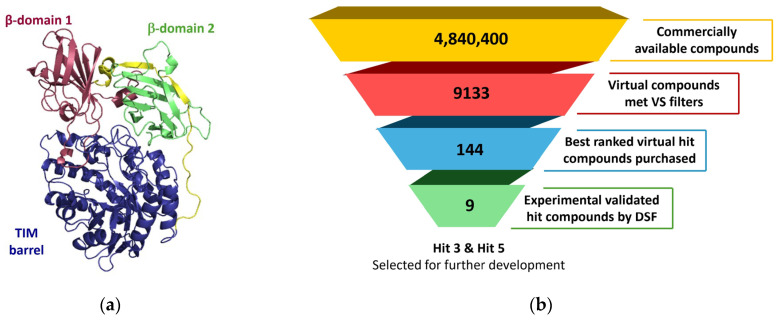
Magellan platform-driven virtual screening for non-competitive, allosteric pharmacological regulators of β-Gal. (**a**) Ribbon diagram of β-Gal monomer used for VS (PDB ID: 3THC), with domains individually colored: β-domain 1 (red), TIM barrel (blue), TIM-β1 loop (yellow), and β-domain 2 (green). (**b**) High-throughput, docking-based virtual screening workflow for identifying small molecules. β-Gal, β-galactosidase; DSF, differential scanning fluorimetry; PDB, Protein Data Bank; TIM, triosephosphate isomerase; VS, virtual screening.

A set of 98 top-scoring molecules from diverse clusters was selected and purchased for experimental validation. These virtual hits were assessed for their ability to stabilize recombinant human β-Gal using a label-free thermal shift assay performed with differential scanning fluorimetry (DSF) [30,31], which identified 3 hit compounds. To further explore the series, 46 commercially sourced analogs of the initial hits were tested using a fluorescence-based thermal shift assay, also conducted with DSF, resulting in 6 additional hits. In total, 144 compounds were tested, yielding 9 hit compounds that were advanced to hit-to-lead optimization (Figure 1).

#### 2.2.2. Experimental Validation of Hit Compounds by Thermal Stability Assessment of Recombinant GLB1 Protein

**Figure 2:**
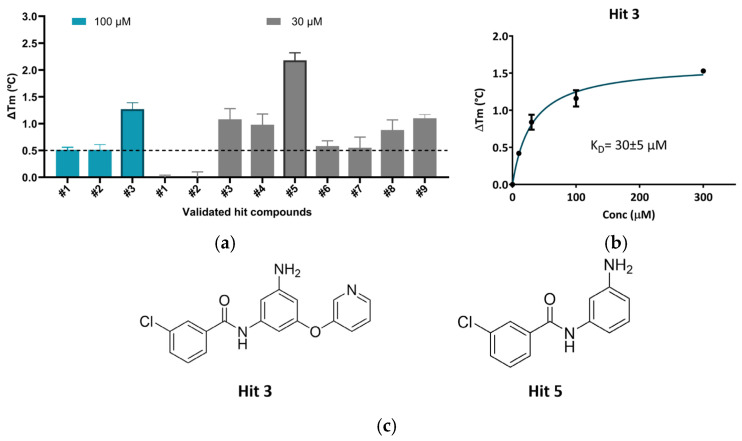
Binding of small-molecule hit compounds to β-Gal protein as measured by DSF. (**a**) Change in melting temperature (ΔTm) of recombinant human β-Gal in the presence of hit compounds #1–3 at 100 μM and #1–9 at 30 μM, measured at pH 7.4. Mean ΔTm ± SD values are shown. The dotted line indicates the DSF screening threshold (ΔTm ≥ 0.5 °C). (**b**) Dose-dependent effect of Hit 3 on β-Gal thermal stability, with mean ΔTm ± SD. (**c**) Chemical structure of Hit 3 and Hit 5. DSF, differential scanning fluorimetry; SD, standard deviation; Tm, melting temperature.

Nine virtual hit compounds that improve β-Gal thermal stability in DSF assays were identified during the initial experimental validation, conducted in two steps (Figure 2a; left panel and Table S1, Supplementary Materials). These validated compounds exhibited a shift in melting temperature (Tm) relative to the baseline, indicating protein stabilization. In the first step, 98 commercial compounds were tested for binding in DSF at a concentration of 100 µM. Among them, Hit 3 emerged as the most promising compound due to its strong stabilization capacity and was prioritized for further characterization. Dose-dependent DSF experiments confirmed the stabilizing effect of Hit 3, with an apparent binding affinity (K*_D_*) of 30 μM at pH 7.4 (Figure 2b).

In the second step, 46 compounds, including analogs of the initial hits, were screened at a lower concentration of 30 µM to identify more potent stabilizers. Notably, Hit 5, an analog of Hit 3, showed a Tm shift of 2.18 °C at 30 µM (Figure 2a; right panel). This screening not only confirmed the stabilizing effect of Hit 3 but also led to the discovery of Hit 5, which demonstrated even greater thermal stabilization capacity. Hits 3 and 5 share a common scaffold and represent a new chemical series (Figure 2c), which serves as the starting point for a focused medicinal chemistry program aimed at developing β-Gal stabilizers that function as PCs.

### 2.3. Immunocytochemistry as a Screening Tool for Evaluating Hit Compounds in GM1 Gangliosidosis Fibroblasts

To evaluate the ability of the compounds to reduce toxic substrate accumulation, we used an immunocytochemistry (ICC) assay, which measures the reduction in exogenously added GM1 ganglioside. This assay has been previously demonstrated as a valuable tool for characterizing lead compounds from various chemical series [23]. In this study, we highlight the potential of the ICC-based assay not only for compound characterization but also as a screening tool to establish structure–activity relationships (SARs). The cellular model used was a canine fibroblast cell line bearing the R60H missense mutation, one of the few commercially available at the time and previously validated [23]. This naturally occurring mutation in canine β-Gal is equivalent to the prevalent human β-Gal mutation R59H [32]. Cultured fibroblasts were supplemented with exogenous GM1 ganglioside, and its intracellular accumulation was monitored by ICC using specific antibodies. As expected, wild-type (WT) canine fibroblasts showed no substrate accumulation due to normal β-Gal function, whereas R60H mutant fibroblasts exhibited high intracellular levels of GM1 ganglioside.

**Figure 3:**
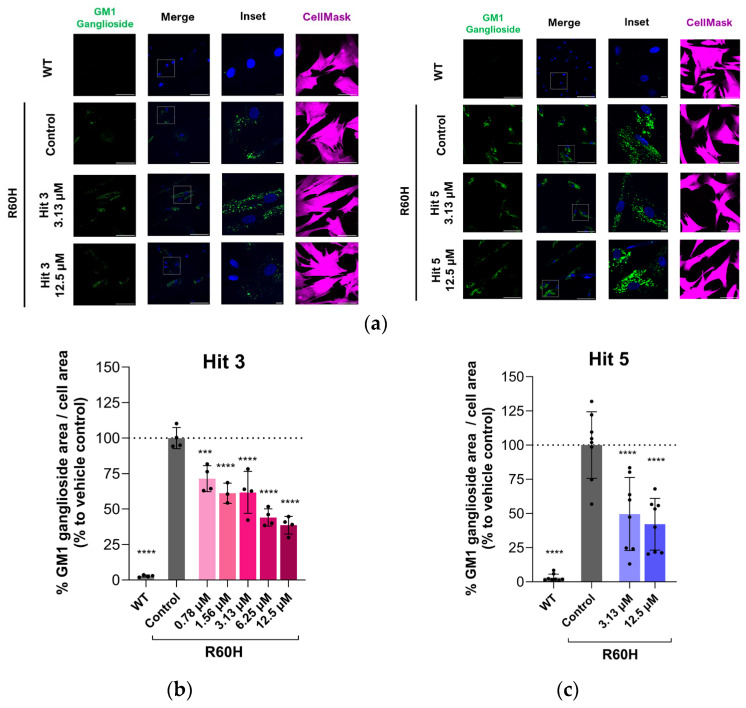
(**a**) Hit 3 (left panel) and Hit 5 (right panel) reduction in GM1 ganglioside accumulation in canine fibroblasts. Representative immunofluorescence images of WT and R60H β-Gal canine fibroblasts (untreated or treated with indicated compounds). Images show GM1 ganglioside antibody staining (first column), merge of GM1 ganglioside staining and DAPI (second column), inset of a region (third column), and CellMask staining (fourth column). Scale bar: 100 μM; inset scale 10 μM. (**b**,**c**) Dose–response of Hit 3 and Hit 5, respectively. Results are presented as mean ± SD. Data represent the percentage of GM1 ganglioside area per cell area relative to untreated R60H canine fibroblasts. Statistical analysis was performed using one-way ANOVA followed by Dunnett’s Multiple Comparison Test. Asterisks indicate statistically significant differences compared to untreated R60H canine fibroblasts: *** *p* < 0.001, **** *p* < 0.0001; WT, wild type.

To test the efficacy of the hit compounds, GM1 gangliosidosis fibroblasts were treated with the compounds for four days before measuring substrate depletion. Both Hit 3 and Hit 5 demonstrated a dose-dependent reduction in exogenous GM1 ganglioside accumulation, indicating that these compounds enable mutant β-Gal to degrade the substrate (Figure 3). As a control, the competitive PC N-nonyl-deoxygalactonojirimycin (NN-DGJ) was included but showed no effect on GM1 ganglioside reduction at concentrations of 25 µM and 1 µM, which were selected to avoid inhibition [23]. Consistent with a non-inhibitory pharmacological chaperone profile, none of the tested STAR compounds inhibited endogenous β Gal activity in biochemical assays using WT human fibroblast lysates, whereas NN-DGJ caused a clear dose dependent inhibition (Supplementary Materials, File S1).

### 2.4. Identification of a Novel Chemical Series of GLB1 STARs Through Scaffold Hopping

**Figure 4:**
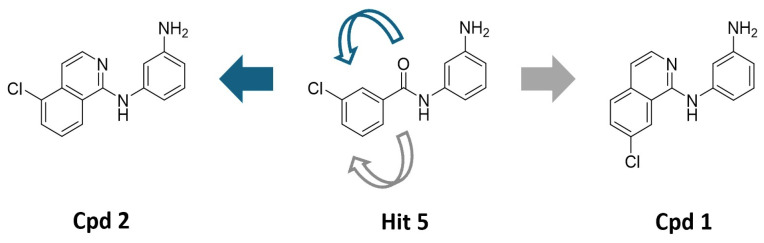
Cyclization of the amide motif in Hit 5 led to the development of a novel chemical series based on a new scaffold. Cpd 1 and Cpd 2 represent the first compounds generated through this scaffold-hopping strategy.

To discover a more active scaffold, a classical medicinal chemistry approach known as scaffold hopping was employed. This design strategy involves replacing the core chemical structure of a known active compound with a novel chemical motif while retaining its biological activity. Applying this strategy to the previously identified hit series scaffold resulted in the development of a new chemical series. This was achieved through a ring closure involving the carbonyl moiety in two possible orientations, leading to the creation of Compounds 1 and 2 (Cpd 1 and Cpd 2) as the first representatives of this series (Figure 4). The detailed synthetic procedure for these compounds and their characterization are provided in the Supplementary Materials (Experimental Procedure and File S2).

### 2.5. Dose-Dependent Efficacy of Cpd 1 and Cpd 2

**Figure 5:**
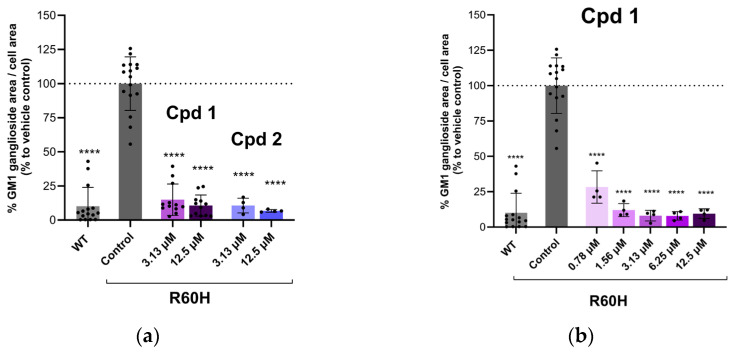
(**a**) Efficacy of Cpd 1 and Cpd 2 at the indicated concentrations in reducing GM1 ganglioside accumulation in R60H canine fibroblasts. (**b**) Dose–response of Cpd 1. Quantification of GM1 ganglioside area per cell area is expressed as a percentage relative to untreated cells. Results are presented as mean ± SD. Statistical analysis was performed using one-way ANOVA followed by Dunnett’s Multiple Comparison Test. Asterisks indicate statistically significant differences compared to untreated R60H canine fibroblasts: **** *p* < 0.0001.

The newly developed compounds were evaluated using the previously described cell-based assay in canine fibroblasts treated with an exogenous substrate. This assay measured the reduction in the exogenously added substrate after compound treatment. Both Cpd 1 and Cpd 2 demonstrated a significant dose-dependent decrease in substrate levels (Figure 5), confirming the successful identification of a new chemical series derived from the original hit series through the scaffold-hopping strategy.

### 2.6. SAR Exploration and Optimization of the Chemical Series

#### 2.6.1. SAR Exploration

**Figure 6:**
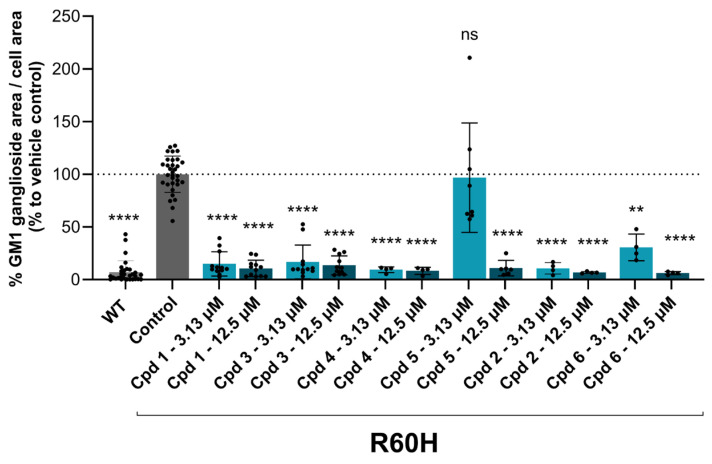
Analogs of Cpd 1 and Cpd 2 reduce GM1 ganglioside accumulation in R60H canine fibroblasts. Quantification of GM1 ganglioside area per cell area as a percentage relative to untreated. Results are presented as mean ± SD. Statistical analysis was performed using one-way ANOVA followed by Dunnett’s Multiple Comparison Test. Asterisks indicate statistically significant differences compared to untreated R60H canine fibroblasts: ns, not significant; ** *p* < 0.01; **** *p* < 0.0001.

Systematic SAR modifications were undertaken to further optimize the newly identified chemical series. The initial set of analogs was synthesized to explore the preferred R1 substituent. Using the scaffold-hopping approach described earlier, molecules with R1 groups (amino pyridines and aniline) were combined with either 5-chloroisoquinoline or 7-chloroisoquinoline. These novel compounds were tested in a cell-based canine fibroblast assay following 4 days of incubation at two screening concentrations: 12.5 μM and 3.13 μM. Preliminary SAR indicates no strong preference between 5- and 7-chloroisoquinoline substitutions, although the 5-chloroisoquinoline may exhibit slightly higher activity (Table 1 and Figure 6). The results also indicated that pyrazine derivatives at R1 were less well accommodated compared to pyridines and phenyl rings. Among the tested compounds, those with pyridines at the R1 position were particularly noteworthy due to their anticipated improved metabolic properties, making them a priority for further development.

#### 2.6.2. Exploration of Substituted Isoquinolines

To further investigate the role of the isoquinoline feature, an initial expansion was performed by designing and synthesizing a new set of 9 compounds. In this SAR exploration, the 2,6-diaminopyridine moiety was retained due to its demonstrated activity, while structural variations were introduced on the isoquinoline portion to assess its contribution to activity. The synthesis followed the same procedure as described for the previous analogs. These compounds were tested in a cell-based canine fibroblast assay after 4 days of incubation at two concentrations: 12.5 μM and 3.13 μM.

**Figure 7:**
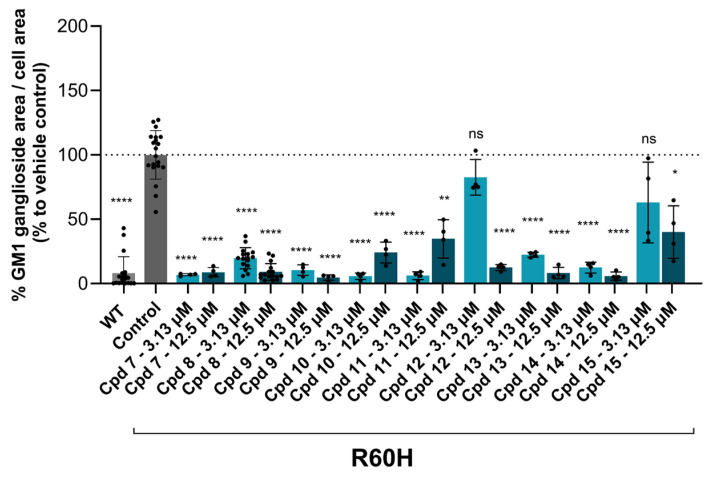
Substituted isoquinolines bearing a 2,6-diaminopyridine moiety reduce GM1 ganglioside accumulation in R60H canine fibroblasts. Quantification of GM1 ganglioside area per cell area is expressed as a percentage relative to untreated. Results are presented as mean ± SD. Statistical analysis was performed using one-way ANOVA followed by Dunnett’s Multiple Comparison Test. Asterisks indicate statistically significant differences compared to untreated R60H canine fibroblasts: ns, not significant; * *p* < 0.05; ** *p* < 0.01; **** *p* < 0.0001.

All tested compounds demonstrated a reduction in the exogenous substrate (Table 2 and Figure 7). A detailed analysis of the SAR data revealed several key findings. Compounds 10 and 11, both featuring a substituent at position 6 of the isoquinoline, showed an unexpected inverse dose response, the cause of which remains unclear. Substitutions at position 7 of the isoquinoline, as seen in Cpd 8, Cpd 13, and particularly Cpd 12, resulted in reduced potency. Cpd 15, which contains a strong electron-withdrawing group (CN) at position 5 of the isoquinoline, was the least active compound, indicating that such groups are poorly tolerated. In contrast, compounds Cpd 7, Cpd 9, and Cpd 14, with electro-donating or weak electron-donating groups at position 5 of the isoquinoline, were the most potent and exhibited preliminary concentration-dependent effects.

#### 2.6.3. Extended Dose–Response Analysis

**Figure 8:**
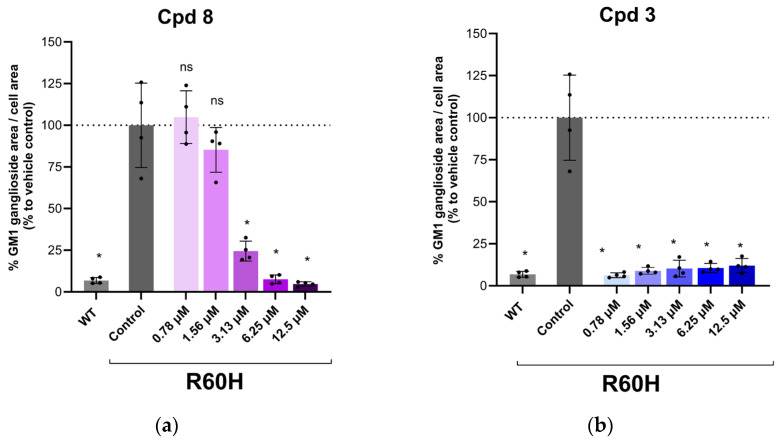
(**a**) Dose–response of Cpd 8. (**b**) Dose–response of Cpd 3. Quantification of GM1 ganglioside area per cell area as a percentage relative to untreated. Results are presented as mean ± SD. Statistical analysis was performed using one-way ANOVA followed by Dunnett’s Multiple Comparison Test. Asterisks indicate statistically significant differences compared to untreated R60H canine fibroblasts: ns, not significant; * *p* < 0.05.

To further validate the observed concentration-dependent effects, a subset of representative compounds was selected for extended dose–response analysis. The results of this analysis are illustrated in Figure 8. The extended dose–response analysis revealed distinct trends among the tested compounds. Cpd 8 demonstrated a clear dose-dependent reduction in GM1 ganglioside accumulation, with activity increasing proportionally to the concentration tested (Figure 8a). In contrast, Cpd 3 exhibited an activity plateau across the tested concentrations, as shown in Figure 8b.

#### 2.6.4. Exploration of Diaminoaryl Moiety

To further explore the contribution of the diaminoaryl fragment to activity, a third set of compounds was synthesized with R1 substituents incorporating pyridine and pyrimidine moieties featuring different nitrogen and diamino patterns. These compounds were designed to bear either 5- or 7-substituted isoquinoline groups and were evaluated for substrate depletion in a cell-based canine fibroblast assay after 4 days of incubation at two concentrations: 12.5 μM and 3.13 μM.

**Figure 9:**
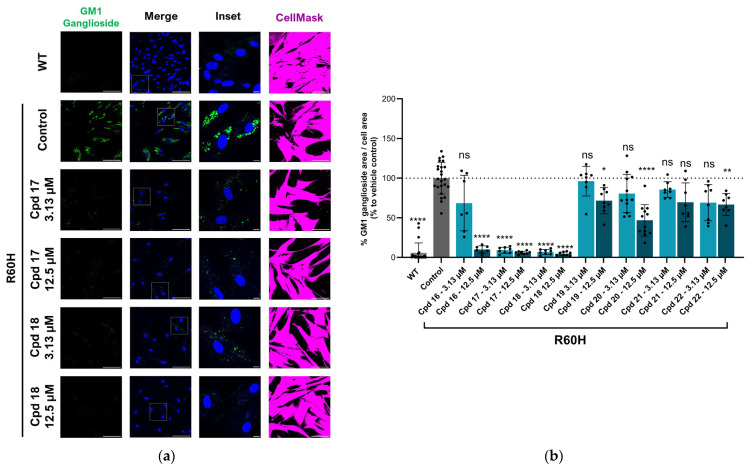
(**a**) Representative immunofluorescence images of WT or R60H β-Gal canine fibroblasts (untreated or treated with indicated compounds). Images show GM1 ganglioside antibody staining (first column), merge of GM1 ganglioside staining and DAPI (second column), inset of a region (third column), and CellMask staining (fourth column). Scale bar: 100 μm; inset scale 10 μm. (**b**) Quantification of GM1 ganglioside area per cell area at 3.13 μM and 12.5 μM concentrations. Results are expressed as a percentage relative to untreated cells and are presented as mean ± SD. Statistical analysis was performed using one-way ANOVA followed by Dunnett’s Multiple Comparison Test. Asterisks indicate statistically significant differences compared to untreated R60H canine fibroblasts: ns, not significant; * *p* < 0.05; ** *p* < 0.01; **** *p* < 0.0001.

The results supported the hypothesis that amino-pyridine substituents at R1 are better tolerated than other amino-heteroaryl groups, such as pyrimidine or pyrazine moieties. Among the tested compounds, Cpd 17 and Cpd 18 demonstrated high potency, confirming that 1,4-diaminopyridine substituents are as active as 1,3-diaminopyridines (Table 3 and Figure 9). The results indicate that diamino-pyridine is the most effective R1 substituent, with both meta and para-amino group configurations being well tolerated. A chloride atom at carbon 5 of the isoquinoline is preferred for optimal activity, though substitution at carbon 7 is also acceptable. These observations highlight key structural features for further optimization.

### 2.7. Confirmation of STARS Binding to β-Gal by Surface Plasmon Resonance (SPR)

The direct binding of a representative selection of compounds to β-Gal was studied using the surface plasmon resonance (SPR). SPR enables quantitative analysis of interactions between biomolecules, such as proteins and small molecules, by monitoring changes in the refractive index near a sensor surface [33,34].

**Figure 10:**
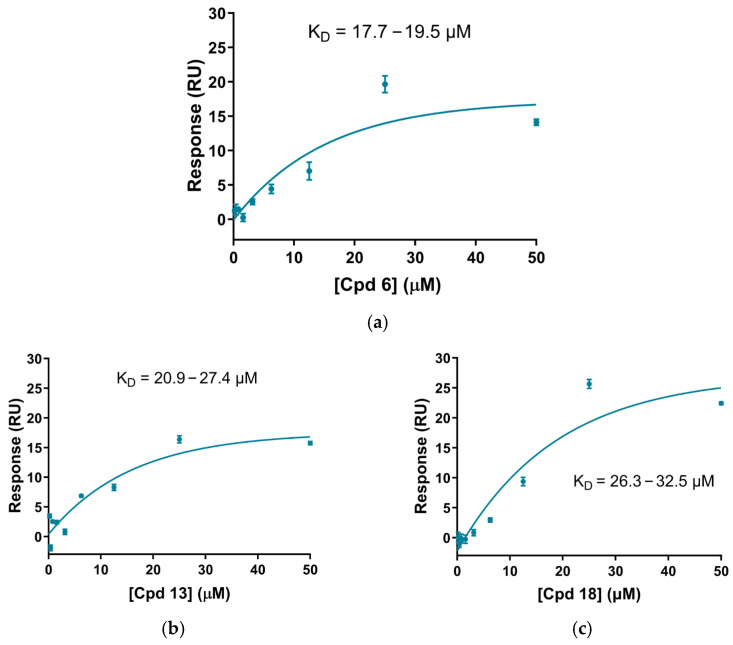
Surface plasmon resonance (SPR) dose–response for compounds (**a**) Cpd 6, (**b**) Cpd 13, and (**c**) Cpd 18, showing their binding to immobilized β-Gal at neutral pH (7.4).

The affinities of binding (K*_D_*) for the tested compounds are summarized in Table 4. Direct binding of STARs to recombinant human β-Gal WT was confirmed in a dose–response manner at neutral pH 7.4 (Figure 10). The results demonstrate that compounds from the novel isoquinoline chemical series bind β-Gal with measurable K*_D_*s. However, no direct correlation was observed between the binding data measured by SPR (expressed as K*_D_*) and the predicted potency in the ICC assay, likely due to the differing parameters involved in each assay. Nevertheless, the SPR data confirms the direct binding of the newly described compounds to the target protein.

### 2.8. BBB Penetration

**Figure 11:**
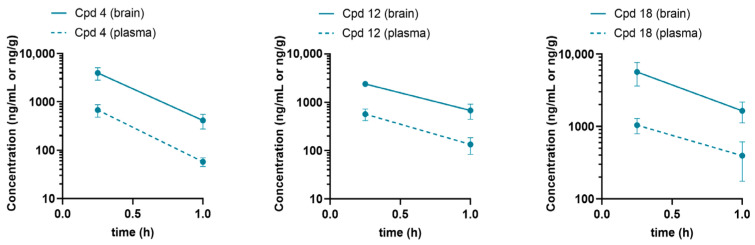
Plasma and brain pharmacokinetics of representative Cpds (4, 12 and 18) were measured at different time points following a single intraperitoneal (i.p.) administration of 10 mg/kg in male C57BL/6 mice. Cpd, compound.

Finally, we evaluated the ability of compounds from the chemical series to penetrate the blood–brain barrier (BBB) and reach the central nervous system (CNS). Representative compounds (Cpd 12, Cpd 18, and Cpd 4) were tested following intraperitoneal (i.p.) administration in male C57BL/6 wild-type (WT) mice. To assess pharmacokinetics (PK) and brain penetrability, compound concentrations were measured in both brain tissue (ng/g) and plasma (ng/mL) at a dose of 10 mg/kg, using the same formulation described in the Methods (5% N-methyl-2-pyrrolidone, 5% Solutol HS-15, and 90% saline). At 0.25 h post-administration, all three compounds exhibited substantial brain exposure, with Cpd 4 displaying the highest brain partition coefficient (K*p* = 5.93), followed by Cpd 18 (K*p* = 5.46) and Cpd 12 (K*p* = 4.40) (Table 5 and Figure 11). Together, these findings indicate that all three compounds effectively penetrate the BBB and achieve relevant exposure in the CNS.

## 3. Discussion

Through the integration of advanced computational approaches, we previously reported the identification of a novel allosteric binding site on β-Gal and the discovery of a chemical series of allosteric modulators, termed STARs. Using an integrated drug discovery pipeline combining Magellan (formerly SEE-Tx^®^) [23], MDmix [27], and rDock [28], we identified small molecules with promising drug-like profiles, showcasing the efficiency and innovation of our approach. In this study, we present these compounds as stabilizing non-inhibitory PCs discovered through an allosteric site-guided approach. Additionally, we validate the applicability of Magellan and our advanced computational methods in identifying novel therapeutic compounds, as validated by exogenous substrate depletion in the ICC-based assay.

Recent studies have highlighted the potential of β-Gal inhibitors as PCs for GM1 gangliosidosis and Morquio B. For instance, compounds such as N-substituted iminosugars and cyclopentanetriols have shown efficacy in stabilizing mutant β-Gal enzymes and improving their function in preclinical models [17,35]. These findings underscore the promise of PCs as a viable therapeutic option, particularly for patients with amenable mutations. However, the development of PCs faces several challenges, including ensuring specificity for mutant β-Gal, achieving effective BBB penetration, and addressing long-term safety concerns. Furthermore, the heterogeneity of *GLB1* mutations complicates PC design, necessitating personalized approaches tailored to individual genotypes [7].

The results of this study address several of these challenges. Notably, STAR compounds demonstrated the ability to cross the BBB and achieve substantial brain exposure, a critical advancement given that effective CNS delivery remains a major limitation of many current therapeutic strategies for LSDs [6,7,36,37]. Representative compounds from the novel chemical series (Cpd 4, Cpd 12, and Cpd 18) exhibited high brain partition coefficients, confirming their potential to target the neurodegenerative aspects of GM1 gangliosidosis. This putative capability is particularly relevant given the severe CNS involvement in GM1 gangliosidosis, where substrate accumulation in the brain leads to progressive neurodegeneration [1,3].

The scaffold-hopping approach employed in this study represents a significant innovation in the development of non-inhibitory PCs. By modifying the core structure of the initial hit compounds, this strategy enabled the generation of a novel chemical series with improved PK properties and enhanced activity. Scaffold hopping is widely recognized as a powerful tool in drug discovery, allowing for the optimization of potency, selectivity, and drug-like properties while maintaining the biological activity of the parent scaffold [38,39]. In this study, scaffold hopping not only improved the physicochemical properties of the compounds but also facilitated the identification of new chemical entities capable of stabilizing β-Gal and reducing toxic substrate accumulation in disease-relevant models.

Despite the promising findings, this study has several limitations. While the compounds demonstrated BBB penetration and favorable PK in mice, their therapeutic efficacy in vivo remains unexplored. Additionally, the study primarily focused on the R60H mutation in canine fibroblasts, leaving the broader applicability of the compounds across diverse β-Gal mutations uncertain. Long-term toxicity data are also lacking. Furthermore, discrepancies between SPR binding affinities and ICC assay potency highlight the need for further investigation into the mechanisms of compound activity. Future studies will focus on addressing these limitations, including comprehensive kinetic and mechanistic characterization, to advance these promising STAR compounds toward clinical application.

## 4. Materials and Methods

### 4.1. Virtual Screening Using Magellan Technology

The published 3D structure of human β-Gal (PDB ID: 3THC), determined by X-ray crystallography at a resolution of 1.8 Å [22], was used as the basis for virtual screening. Molecular dynamics simulations in organic-aqueous solvent mixtures (MDmix) identified a druggable cavity and key interaction sites (binding hot spots) [27]. These hot spots were applied as pharmacophoric restraints to guide docking and explore the flexibility of the binding site. A virtual library of approximately 5 million compounds available from commercial suppliers -Asinex (Moscow, Russia), Enamine (Kyiv, Ukraine), Key Organics (Camelford, United Kingdom), Life Chemicals (Kyiv, Ukraine), and Specs (Zoetermeer, The Netherlands)- was screened computationally using the docking program rDock [28]. The screening employed a high-throughput protocol with the standard scoring function and pharmacophoric restraints.

### 4.2. Differential Scanning Fluorimetry (DSF) Assay

The ability of compounds to stabilize β-Gal was assessed using a DSF assay. Thermal denaturation of purified human β-Gal was monitored with SYPRO Orange (Thermo Fisher Scientific, Waltham, MA, USA), which binds to hydrophobic parts of proteins that are exposed as the protein unfolds. Two independent experiments were conducted in triplicate. In the first round, 98 compounds (High Initial Diversity; HID) were tested at 100 μM, while in the second round, 3 validated hits and 46 additional compounds (High Affinity; HA) were tested at 30 μM. Each 25 μL reaction contained 12.5 μL of 1.5 μM recombinant human β-Gal protein (rhGLB1; Novoprotein, Shanghai, China) in PBS (pH 7.4), resulting in a final protein concentration of 1 μM, and 12.5 μL of compound solution dissolved in 100% DMSO and diluted in protein buffer to achieve a final DMSO concentration of 2%. SYPRO Orange was added at a final 10× concentration.

Fluorescence intensity was monitored as the temperature increased using a Roche LightCycler 480 II (Roche Diagnostics, Mannheim, Germany). Melting temperature (Tm) shifts were considered significant if the absolute ΔTm was ≥0.5 °C (instrumental criterion) and the ΔTm standard deviation (SD) was ≤0.2 °C (statistical criterion). Thermal shift dose–response curves were measured twice for Hit 3 at concentrations ranging from 10 to 300 μM in triplicate with rhGLB1.

DSF assays were performed at the Genomics Unit of the Scientific and Technological Centers (CCiTUB), Universitat de Barcelona.

### 4.3. Immunocytochemistry and Microscopy Imaging

#### 4.3.1. Cell Culture

WT β-Gal fibroblasts (Stemnovate Ltd., Cambridge, United Kingdom; ref. SV-2025-V04) and R60H canine fibroblasts (Coriell Institute for Medical Research, Camden, NJ, USA; ref. GM11473) were cultured in DMEM (Gibco, Thermo Fisher Scientific, Grand Island, NY, USA; ref. 11574486) supplemented with 10% fetal calf serum (FCS, Gibco, ref. 10500) and 1% penicillin/streptomycin (P/S, Gibco, ref. 15140122) at 37 °C and 5% CO_2_. Cells were seeded in 96-well plates (Ibidi GmbH, Gräfelfing, Germany; ref. 89626). After 48 h, GM1 bovine ganglioside (Sigma-Aldrich, St. Louis, MO, USA; ref. G7641) was added at a final concentration of 0.1 mg/mL. Compounds or DMSO (vehicle control) were added at the indicated concentrations for four days.

#### 4.3.2. Fixing and Staining

Canine fibroblasts were fixed with 4% paraformaldehyde (Electron Microscopy Sciences, Hatfield, PA, USA; ref. 15710) in PBS for 15 min at room temperature (RT). Cells were permeabilized with 0.3% Triton X-100 (Sigma-Aldrich, St. Louis, MO, USA; ref. T8787) in PBS for 15 min at RT. Cytoplasm was labeled with HCS CellMask (Thermo Fisher Scientific, Waltham, MA, USA; ref. T8787) for 15 min at RT (Table 6). Blocking was performed for 1 h at RT in PBS containing 0.5% bovine serum albumin (BSA, Sigma-Aldrich, ref. A9647) and 10% normal donkey serum (EMD Millipore, Billerica, MA, USA; ref. S30).

The primary antibody against ganglioside GM1 (Abcam, Cambridge, United Kingdom; ref. ab23943) was incubated overnight at 4 °C, followed by Alexa Fluor^®^ 488 Donkey anti-Rabbit IgG (Invitrogen, Carlsbad, CA, USA; ref. A21206) for 1 h at RT. Nuclear counterstaining was performed with DAPI (Thermo Fisher Scientific, Waltham, MA, USA; ref. 62248) in PBS for 10 min at RT. Fibroblasts were washed and stored at 4 °C in PBS until imaging.

#### 4.3.3. Imaging and Analysis

Stained cells were imaged using a LIPSI Nikon microscope (Nikon Europe B.V., Amsterdam, The Netherlands) with a 40× water immersion objective (NA = 1.15). For each well, 25 images per channel were acquired with a 10% overlap (5 × 5 grid) and a frame size of 2048 × 2048 pixels. Images were stitched into a single composite image per well using NIS-Elements AR 5.21 software. Cell area was defined using CellMask staining, and nuclei were identified with DAPI. The intensity, size, and number of green dots (representing GM1 ganglioside) were quantified within the cytoplasm, excluding the nuclei area. A ratio of GM1 ganglioside area to total cell area was calculated to account for cell size differences. In addition, only cells containing nuclei were included in the analysis.

#### 4.3.4. Statistical Analysis

Statistical analysis was performed using GraphPad Prism 10.6.1 (GraphPad Software, San Diego, CA, USA). Data are expressed as mean ± SD. One-way ANOVA with Dunnett’s multiple comparisons test was used to assess significance (*p* < 0.05) (for Figure 3b,c, Figure 5a,b, Figure 6, Figure 7, Figure 8a,b and Figure 9b). ICC assays were conducted at the Advanced Digital Microscopy Facility of IRB Barcelona.

### 4.4. Surface Plasmon Resonance (SPR) Binding Assay

All SPR experiments were conducted at 20 °C using a Biacore T200 (GE Healthcare, Chicago, IL, USA). SPR detects changes in refractive index due to mass changes on the sensor surface, enabling measurement of biomolecular interactions.

#### 4.4.1. Protein Immobilization

Recombinant human β-Gal (rhGLB1) from a Chinese hamster ovary (CHO) cell line, featuring a C-terminal 6-his tag (Met1-Val677) in 25 mM Tris and 150 mM NaCl, pH 7.5 (Bio-Techne, Minneapolis, MN, USA), was immobilized on a CM5 SPR sensor chip (GE Healthcare, Chicago, IL, USA; CM5, ref. 29149603) using standard amine coupling. A protein concentration of 100 µg/mL in 10 mM sodium acetate buffer (pH 4.0) was used to achieve a protein density of approximately 10,000 Response Units (RU) (10,000 RU on channel 2 and 12,700 RU on channel 3). PBS (pH 7.4) served as the running buffer during immobilization. Empty, activated, and deactivated parallel channels on the same sensor chip were used as reference channels.

#### 4.4.2. Binding Studies

Small-molecule ligands were diluted in running buffer (PBS with 5% DMSO, pH 7.4) from 10 mM DMSO stock solutions to create a 9-point, 2-fold serial dilution series up to 50 µM. Compounds were titrated in duplicate over the β-Gal surface. NN-DGJ, a reference compound with a reported K*_D_* of 1.1 µM, was used to validate binding activity.

#### 4.4.3. Data Analysis

Raw SPR signals from the active channel were double referenced by subtracting signals from the reference channel and running buffer. DMSO signal mismatches were corrected. Binding affinity values were calculated using a four-parameter logarithmic dose–response equation (GraphPad Prism 10.6.1) without constraint. SPR assays were performed on the Biomolecular Analysis Unit from Scientific and Technological Centers (CCiTUB), Universitat de Barcelona.

### 4.5. BBB Penetration (NeuroPK Assay)

The brain penetration of Cpd 12, Cpd 18, and Cpd 4 was evaluated following i.p. administration. Six male C57BL/6 mice received a 10 mg/kg dose of each compound in a formulation of 5% NMP, 5% Solutol^®^ HS-15, and 90% saline.

#### 4.5.1. Sample Collection and Quantification

Blood samples (approximately 150 µL) were collected under light isoflurane anesthesia from three mice at 0.25 and 1 h. Plasma was separated by centrifugation and stored at −70 ± 10 °C until analysis. Immediately after collecting blood, whole-body perfusion was performed using 10 mL PBS and brain samples were collected from each mouse at respective time points. Tissue samples were homogenized using ice-cold PBS (pH 7.4), and the homogenates were stored below −70 °C until analysis. The total homogenate volume was three times the brain weight. The concentration–time data from plasma and brain samples were used for pharmacokinetic analysis. Both plasma and brain samples were quantified using a fit-for-purpose liquid chromatography–tandem mass spectrometry (LC-MS/MS) method, with a lower limit of quantification (LLOQ) of 1.01 ng/mL for plasma and 2.03 ng/mL for brain.

#### 4.5.2. LC-MS/MS Conditions

Analyses were performed on a Kinetex™ XB-C18 column (1.7 µM, 50 mm × 2.1 mm) with a 2.2 min run time, 2 µL injection volume, and 0.6 mL/min flow rate. The mobile phase consisted of 0.1% formic acid in acetonitrile (A) and 0.1% formic acid in water (B) with the following gradient: 0.00 min/98% B, 0.30 min/98% B, 0.50 min/2% B, 1.40 min/2% B, 1.60 min/98% B, and 2.20 min/98% B.

## 5. Conclusions

This study details the discovery, SAR exploration, and biological evaluation of a novel chemical series exhibiting non-inhibitory PC activity on β-Gal. Through SAR optimization, we identified compounds capable of stabilizing the β-Gal structure, as demonstrated by thermal stabilization assays and the enhancement of exogenous toxic substrate clearance in cellular models. PK analyses further confirmed that these compounds can cross the BBB, addressing a critical challenge for treating diseases with neuropathic involvement linked to β-Gal deficiency.

The scaffold-hopping approach employed in this study provides a robust framework for the optimization of PCs, enabling the development of compounds tailored to specific *GLB1* mutations. This personalized approach could address the heterogeneity of *GLB1*-related disorders, offering hope for patients with diverse genotypes. Additionally, the scaling-up and automation of the ICC screening assays described in this study could enable the development of a robust, high-throughput tool for identifying PCs that stabilize and improve the intracellular trafficking of mutant lysosomal enzymes.

## Figures and Tables

**Table 1:** Quantification of GM1 ganglioside area relative to cell area, expressed as a percentage of the vehicle control (DMSO), for Cpd 1 and Cpd 2 analogs at concentrations of 12.5 μM and 3.13 μM.

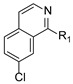				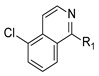			
**R_1_**	**Cpd**	**% GM1 Ganglioside** **Area/Cell Area** **(% to Vehicle Control)**		**R_1_**	**Cpd**	**% GM1 Ganglioside** **Area/Cell Area** **(% to Vehicle Control)**	
		**12.5 µM**	**3.13 µM**			**12.5 µM**	**3.13 µM**
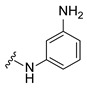	**1**	11	15	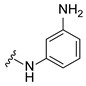	**2**	7	11
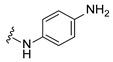	**3**	14	17	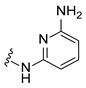	**6**	6	31
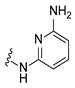	**4**	8	9				
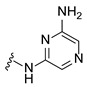	**5**	11	97				

Cpd, compound.

**Table 2:** Quantification of GM1 ganglioside area relative to cell area, expressed as a percentage of untreated, for substituted isoquinolines containing a 2,6-diaminopyridine moiety at concentrations of 12.5 μM and 3.13 μM.

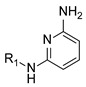							
**R_1_**	**Cpd**	**% GM1 Ganglioside** **Area/Cell Area** **(% to Vehicle Control)**		**R_1_**	**Cpd**	**% GM1 Ganglioside** **Area/Cell Area** **(% to Vehicle Control)**	
		**12.5 µM**	**3.13 µM**			**12.5 µM**	**3.13 µM**
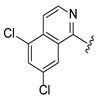	**7**	9	7	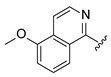	**12**	12	83
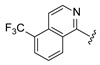	**8**	9	20	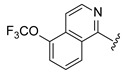	**13**	8	22
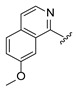	**9**	5	12	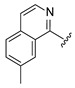	**14**	6	12
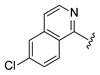	**10**	24	6	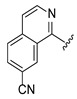	**15**	40	63
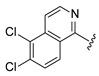	**11**	35	6				

Cpd, compound.

**Table 3:** Quantification of GM1 ganglioside area relative to cell area, expressed as a percentage of the vehicle control (DMSO), for 5- and 7-substituted isoquinoline bearing various pyridine or pyrimidine R1 groups at concentrations of 12.5 μM and 3.13 μM.

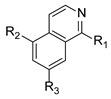					
**R_1_**	**R_2_**	**R_3_**	**Cpd**	**% GM1 Ganglioside Area/Cell Area** **(% to Vehicle Control)**	
				**12.5 µM**	**3.13 µM**
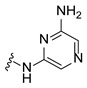	-CF_3_	-H	**16**	10	69
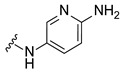	-Cl	-H	**17**	6	9
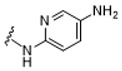	-Cl	-H	**18**	5	7
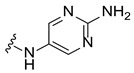	-Cl	-H	**19**	72	96
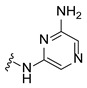	-OMe	-H	**20**	47	80
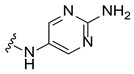	-H	-CN	**21**	70	86
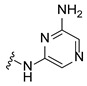	-H	-CN	**22**	67	69

Cpd, compound.

**Table 4:** SPR direct binding affinities (K*_D_*) measured at pH 7.4 for selected compounds.

Compound	Dose Response (pH 7.4)	K*_D_* (µM)
NN-DGJ (control)	Yes	0.37–1.12
Cpd 6	Yes	17.7–19.5
Cpd 8	Yes	21.5–24.3
Cpd 10	Yes	36.9–45.8
Cpd 11	Yes	38.3–46.3
Cpd 12	Yes	39.5–61
Cpd 13	Yes	20.9–27.4
Cpd 16	Yes	60.1–62.8
Cpd 17	Yes	72.2–110
Cpd 18	Yes	26.3–32.5
Cpd 19	Yes	21–23.7

Cpd, compound; K*_D_*, dissociation constant; NN-DGJ, N-nonyl-deoxygalactonojirimycin; SPR, surface plasmon resonance.

**Table 5:** Summary of plasma (ng/mL) and brain tissue (ng/g) concentrations of compounds Cpd 12, Cpd 18, and Cpd 4 at specified times post-administration.

Compound	Time (h)	Plasma Concentration (ng/mL)	Brain Concentration (ng/g)	Brain-K*p*
Cpd 18 (i.p. 10 mg/kg)	0.25	1042	5647	5.46
	1	395	1645	4.50
Cpd 12 (i.p. 10 mg/kg)	0.25	570	2389	4.40
	1	134	678	5.32
Cpd 4 (i.p. 10 mg/kg)	0.25	678	3950	5.93
	1	58	412	7.70

Cpd, compound; h, hour; K*p*, partition coefficient; i.p., intraperitoneal.

**Table 6:** Summary of antibodies used.

Antibody	Manufacturer	Catalog Number/Clone	Dilution
HCS CellMask	ThermoFisher	T8787	1:5000 (IF)
Ganglioside GM1	Abcam	ab23943	1:500 (IF)
Alexa Fluor^®^ 488 Donkey anti-Rabbit IgG	Invitrogen	A21206	1:2000 (IF)

IF, immunofluorescence.

## Data Availability

The data presented in this study is available on request from the corresponding author. The data is not publicly available due to company confidentiality restrictions.

## Supplementary Materials

The following supporting information can be downloaded at: https://www.mdpi.com/article/10.3390/ijms27083631/s1.

## Abbreviations

The following abbreviations are used in this manuscript:

ANOVA	Analysis of variance
BBB	Blood–brain barrier
β-Gal	Beta-galactosidase
BSA	Bovine Serum Albumin
CHO	Chinese hamster ovary
CNS	Central nervous system
Cpd	Compound
DAPI	4′,6-diamidino-2-phenylindole
DMEM	Dulbecco’s Modified Eagle Medium
DMSO	Dimethyl sulfoxide
DSF	Differential scanning fluorimetry
ERT	Enzyme replacement therapy
FCS	Fetal calf serum
GLB1	Galactosidase beta 1
GM1	Monosialotetrahexosylganglioside
ICC	Immunocytochemistry
i.p.	Intraperitoneal
K*_D_*	Dissociation constant
Kp	Partition coefficient
LLOQ	Lower Limit of Quantification
LC-MS/MS	Liquid chromatography–tandem mass spectrometry
LSDs	Lysosomal storage disorders
MDmix	Molecular Dynamics in Organic–Aqueous Solvent Mixtures
MPS IV	Mucopolysaccharidosis type IVB
NMP	N-methyl-2-pyrrolidone
NN-DGJ	N-nonyl-deoxygalactonojirimycin
PBS	Phosphate-buffered saline
PC	Pharmacological chaperone
PK	Pharmacokinetics
RT	Room temperature
RU	Resonance units
SAR	Structure–activity relationship
SD	Standard deviation
SEE-Tx	Site-directed enzyme enhancement therapy
SPR	Surface Plasmon Resonance
SRT	Substrate reduction therapy
STARs	Structurally targeted allosteric regulators
Tm	Melting temperature
VS	Virtual screening
WT	Wild type
