# Supplementary material for: Development of Small-Molecule Allosteric Modulators of Beta-Galactosidase (β-Gal) for the Treatment of GM1 Gangliosidosis and Morquio B
Source: Int J Mol Sci. 2026 Apr 18;27(8):3631. doi: 10.3390/ijms27083631 (PMC13115887; doi:10.3390/ijms27083631)

VITAS M CHEMICAL LIMITED

Vitas M Chemical Limited, 15F, Radio City, 505 Hennessy Road,
Causeway Bay, Hong Kong. Tel. +852 98659192
e-mail: irina@vitasmlab.com, www.vitasmlab.com.

Certificate of Analysis

**Product:**<br>N-[3-amino-5-(pyridin-3-yloxy)phenyl]-3-chlorobenzamide		
Lot No./ID : STL170208	Mfg. Date: N/A	Exp Date: N/A
Packed Qty: 10 Date of sampling: 23.05.2013		
Test	Specifications	Observations
**Description**<br><br>**Compound Structure/Name**<br><br>N-[3-amino-5-(pyridin-3-yloxy)phenyl]-3-chlorobenzamide<br><br>**Identification:**<br>i) ^{1}H-NMR	Shall comply	N-[3-amino-5-(pyridin-3-yloxy)phenyl]-3-chlorobenzamide<br><br>Complies

This is a computer generated document. No signature is required.

Lot No./ID : STL170208. Identification: ^{1}H-NMR

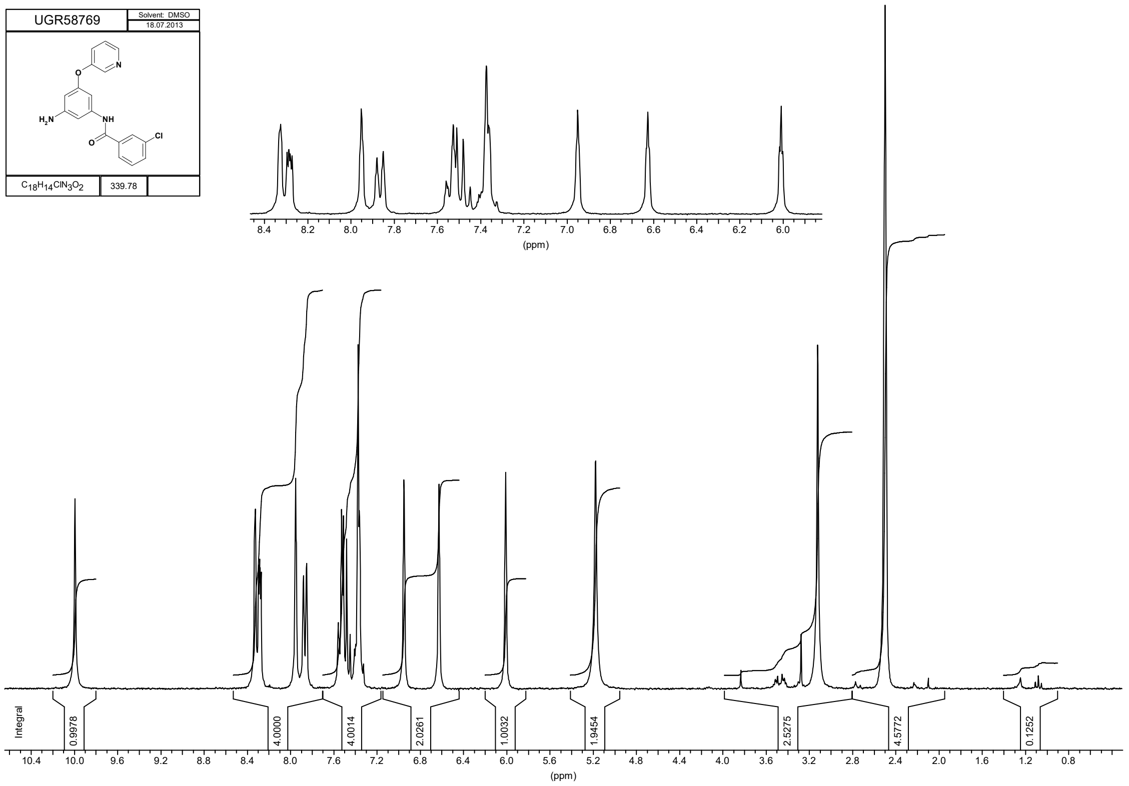

Supplement: Supplementary file 1 [file ijms-27-03631-s001.zip › CoA_Hit3.pdf]
