# Supplementary material for: Development of Small-Molecule Allosteric Modulators of Beta-Galactosidase (β-Gal) for the Treatment of GM1 Gangliosidosis and Morquio B
Source: Int J Mol Sci. 2026 Apr 18;27(8):3631. doi: 10.3390/ijms27083631 (PMC13115887; doi:10.3390/ijms27083631)
Supplement: Supplementary file 1 [file ijms-27-03631-s001.zip › Experimental Procedure.pdf]

Experimental Procedure (synthesis of compounds)

General Experimental Methods

Solvents were distilled and dried using standard methods prior to use. Unless otherwise specified, ^1H NMR spectrum was recorded in CD_3OD, or DMSO-d_6 solutions using a Varian 400 MHz spectrometer. Chemical shifts (δ) are reported in parts per million (ppm) relative to the residual solvent peak, and the coupling constants (J) are reported in hertz (Hz). Reaction progress was monitored by thin-layer chromatography (TLC) and liquid chromatography-mass spectrometry (LC-MS) to determine consumption of starting materials. MW (molecular weight) calculated is an isotopic average and the "found mass" is referring to the most abundant isotope detected in the LC-MS.

Hereinafter, the term “h” means hours, “eq” means equivalents, “min” means minutes, “$Pd_2(dba)_3$” means tris(dibenzylideneacetone)-dipalladium(0), “XantPhos” means 4,5-Bis(diphenylphosphino)-9,9-dimethylxanthene; “$SnCl_2$” means tin(II) chloride; “DCM” means dichloromethane; “MeOH” means methanol; “EtOAc” means ethyl acetate.

High-performance liquid chromatography (HPLC):

The HPLC measurements were performed using a HPLC Waters Alliance HT comprising a pump (Edwards RV12) with degasser, an autosampler, a diode array detector and a column as specified in the respective methods below. Flow from the column was split to a MS spectrometer. The MS detector was configured with an electrospray ionization source (micromass ZQ4000), Nitrogen was used as the nebulizer gas. Data acquisition was performed with MassLynx software.

<u>Method A:</u>

The reverse phase HPLC purifications were carried out on a YMC-Pack ODS-AQ (50x4.6 mm, D S. 3 µm, 12 nm). Solvent A: water 0.1% formic acid; Solvent B: acetonitrile with 0.1% formic acid. Gradient: 5% of B to 100% of B within 3.5 min. Flow rate: 1.6 mL/min at 50ºC.

Method B:

The reverse phase HPLC purifications were carried out on a Xbridge™ C18 3.5 □m (50X4.6mm). Solvent A: NH_4HCO_3 10mM pH9; Solvent B: acetonitrile. Gradient: 5% of B to 100% of B within 3.5 min. Flow rate: 1.6 mL/min at 50ºC.

Synthesis of Compound 1 (*N¹*-(7-chloroisoquinolin-1-yl)benzene-1,3-diamine)

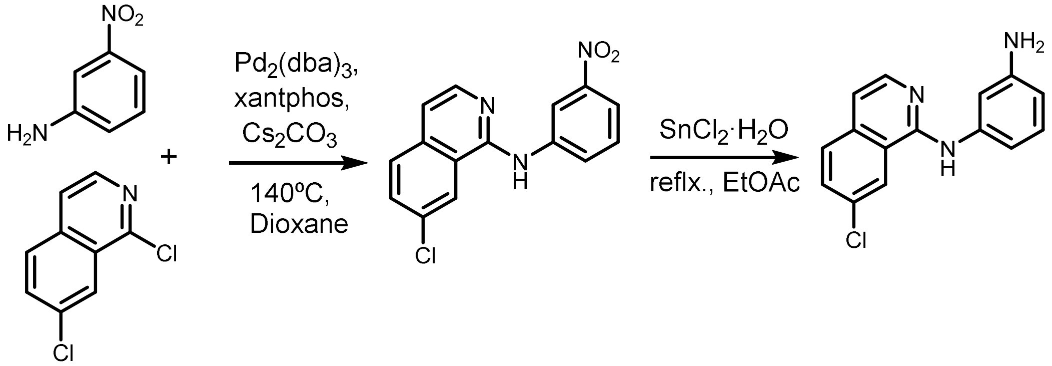

A mixture of 1,7-dichloroisoquinoline (1 eq.), 3-nitroaniline (1.5 eq.), $Pd_2(dba)_3$ (0.1 eq.), XantPhos (0.2 eq.), and Cs_2CO_3 (2 eq.) was dissolved in pre-degassed dioxane (5.3 mL/mmol). The reaction mixture was heated at 140ºC for 5h under a nitrogen atmosphere. Upon completion, the mixture was filtered through a Celite pad and concentrated under reduced pressure. The filtrate was extracted with EtOAc, washed with brine, and dried over anhydrous Na_2SO_4. The crude product was purified by flash column chromatography (hexanes/EtOAc) to afford the desired product 7-chloro-*N*-(3-nitrophenyl)isoquinolin-1-amine (yield: 40%). HPLC-MS (Method B): Rt= 3.35 min, $[M+H]^+$ m/z 300, 301.

A mixture of the nitro intermediate 7-chloro-*N*-(3-nitrophenyl)isoquinolin-1-amine and $SnCl_2 \cdot H_2O$ (4 eq.) in EtOAc (12 mL/mmol) was refluxed overnight. The obtained solution was washed with $NaHCO_3$ (sat. sol.) (5x) and extracted with EtOAc (3x). The combined organic layers were dried with $MgSO_4$, filtered, and concentrated under vacuum. The residue was purified by flash column chromatography (DCM/MeOH) to yield the desired amino product *N¹*-(7-chloroisoquinolin-1-yl)benzene-1,3-diamine (Cpd 1) (yield: 63%). HPLC-MS (Method B): Rt= 1.65 min (98%), $[M+H]^+$ m/z 270, 272. ^1H NMR (400 MHz, DMSO-d_6) δ 8.94 (s, 1H), 8.68 (s, 1H), 7.98 (d, *J* = 5.7 Hz, 1H), 7.84 (d, *J* = 8.7

Hz, 1H), 7.70 (dd, J = 8.7, 2.0 Hz, 1H), 7.14 (d, J = 5.8 Hz, 2H), 6.97 – 6.91 (m, 2H), 6.30 – 6.19 (m, 1H), 5.00 (s, 2H).

The following compounds were synthesized following the same synthetic route as Cpd 1.

Compound 2 (N^1-(5-chloroisoquinolin-1-yl)benzene-1,3-diamine)

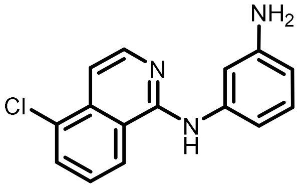

3-nitroaniline was reacted with 1,5-dichloroisoquinoline to afford 5-chloro-N-(3-nitrophenyl)isoquinolin-1-amine (yield: 40%). HPLC-MS (Method B): Rt= 3.15 min, $[M+H]^+$ m/z 300.

5-chloro-N-(3-nitrophenyl)isoquinolin-1-amine was treated with $SnCl_2 \cdot H_2O$ to obtain Cpd 2 (yield: 75%). HPLC-MS (Method B): Rt= 2.58 min (96%), $[M+H]^+$ m/z 270, 272. ^1H NMR (400 MHz, CD_3OD) δ 8.19 (dt, J = 8.5, 1.0 Hz, 1H), 7.88 (d, J = 6.1 Hz, 1H), 7.69 (dd, J = 7.6, 0.9 Hz, 1H), 7.42 (dd, J = 8.4, 7.6 Hz, 1H), 7.31 (d, J = 6.0 Hz, 1H), 6.99 (t, J = 8.0 Hz, 2H), 6.81 (d, J = 7.8 Hz, 1H), 6.39 (ddd, J = 7.9, 2.2, 0.9 Hz, 1H).

Compound 3 (N^1-(7-chloroisoquinolin-1-yl)benzene-1,4-diamine)

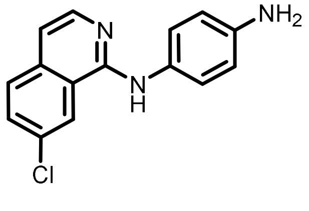

4-nitroaniline was reacted with 1,7-dichloroisoquinoline to afford 7-chloro-*N*-(4-nitrophenyl)isoquinolin-1-amine (yield: 13%). HPLC-MS (Method B): Rt= 3.30 min, $[M+H]^+$ m/z 300, 302.

7-chloro-*N*-(4-nitrophenyl)isoquinolin-1-amine was treated with $SnCl_2 \cdot H_2O$ to obtain Cpd 3 (yield: 11%). HPLC-MS (Method B): Rt= 1.62 min (94%), $[M+H]^+$ m/z 270, 272. ^1H NMR (400 MHz, CD_3OD) δ 8.39 (d, J = 1.9 Hz, 1H), 7.76 (s,

1H), 7.73 (dd, *J* = 11.1, 2.3 Hz, 1H), 7.65 – 7.61 (m, 1H), 7.30 – 7.24 (m, 2H), 7.01 (d, *J* = 5.9 Hz, 1H), 6.79 – 6.74 (m, 2H).

Compound 18 (N^2-(5-chloroisoquinolin-1-yl)pyridine-2,5-diamine)

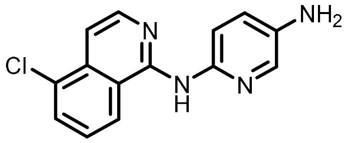

5-nitropyridin-2-amine was reacted with 1,5-dichloroisoquinoline to afford 5-chloro-*N*-(5-nitropyridin-2-yl)isoquinolin-1-amine , which was used directly in the subsequent reaction without further purification (yield: quantitative). HPLC-MS (Method A): Rt= 2.72 min, $[M+H]^+$ m/z 300, 302.

5-chloro-*N*-(5-nitropyridin-2-yl)isoquinolin-1-amine was treated with $SnCl_2{\cdot}H_2O$ to obtain Cpd 18 (yield: 77%). HPLC-MS (Method A): Rt= 1.78 min (100%), $[M+H]^+$ m/z 271, 273. ^{1}H NMR (400 MHz, CD_3OD) δ 8.29 (d, *J* = 8.4 Hz, 1H), 8.07 (s, 1H), 7.81 (dd, *J* = 7.6, 0.9 Hz, 1H), 7.58 – 7.52 (m, 1H), 7.46 (t, *J* = 7.9 Hz, 2H), 7.38 (s, 1H), 6.25 (d, *J* = 8.7 Hz, 1H).

Synthesis of Compound 6 (*N*^{2}-(5-chloroisoquinolin-1-yl)pyridine-2,6-diamine)

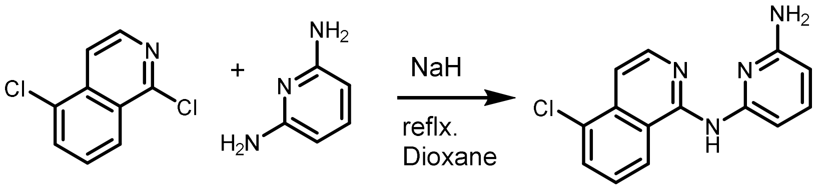

A mixture of pyridine-2,6-diamine (2.2 eq.) and sodium hydride (2.6 eq.) in dry dioxane was stirred for 30 min at room temperature (Rt). Then, 1,5-dichloroisoquinoline (1 eq.) was added portion-wise and the mixture was refluxed overnight. On cooling, water (2 mL) was added, and the solvent was removed under vacuum. The reaction mixture was diluted with ethyl acetate (EtOAc) and washed with water. The combined organic layers were dried over anhydrous $MgSO_4$, filtered, and concentrated under vacuum. The crude mixture was purified by flash column chromatography (hexanes/ EtOAc) to obtain the desired amino

product N^2-(5-chloroisoquinolin-1-yl)pyridine-2,6-diamine (yield: 17%). Pale yellow solid. HPLC-MS (Method A): Rt= 3.3 min (98.7%), $(M+H)^+$ m/z 271, 273.

^1H NMR (400 MHz, DMSO-d_6): δ 9.26 (s, 1H), 8.58 (d, J = 8.4 Hz, 1H), 8.24 (d, J = 6.0 Hz, 1H), 7.95 (d, J = 7.6 Hz, 1H), 7.61 (t, J = 8.0 Hz, 1H), 7.52-7.48 (m, 2H), 7.43 (t, J = 8.0 Hz, 1H), 6.20 (d, J = 8.0 Hz, 1H), 5.71 (s, 2H).

Synthesis of Compound 4 (N^2-(7-chloroisoquinolin-1-yl)pyridine-2,6-diamine)

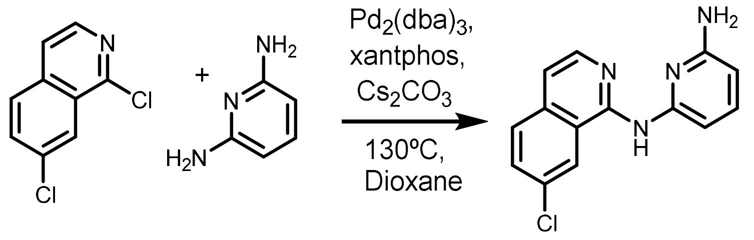

A mixture of 1,7-dichloroisoquinolin (1 eq.), pyridine-2,6-diamine(1.5 eq.), $Pd_2(dba)_3$ (0.1 eq.), XantPhos (0.2 eq.), and Cs_2CO_3 (2 eq.) was dissolved in pre-degassed dioxane (5.3 mL/mmol). The reaction mixture was heated at 130ºC for 2h under a nitrogen atmosphere. Upon completion, the mixture was filtered through a Celite pad and concentrated under reduced pressure. The filtrate was diluted with water, extracted with EtOAc, washed with brine, and dried over anhydrous Na_2SO_4. The crude product was purified by flash column chromatography (hexanes/EtOAc) to afford the desired product N^2-(7-chloroisoquinolin-1-yl)pyridine-2,6-diamine. (yield: 26%). Yellow solid. HPLC-MS (Method A): Rt= 1.85 min (98%), $[M+H]^+$ m/z 271, 273. ^1H NMR (400 MHz, DMSO-d_6) δ 9.25 (s, 1H), 8.70 (s, 1H), 8.08 (d, J = 5.1 Hz, 1H), 7.88 (d, J = 8.5 Hz, 1H), 7.72 (d, J = 8.8 Hz, 1H), 7.45 (d, J = 8.0 Hz, 1H), 7.37 (t, J = 7.8 Hz, 1H), 7.28 (d, J = 5.2 Hz, 1H), 6.13 (d, J = 7.5 Hz, 1H), 5.64 (s, 2H).

The following compounds were synthesized following the same synthetic route as Cpd 4.

Compound 7 (N^2-(5,7-Dichloroisoquinolin-1-yl)pyridine-2,6-diamine)

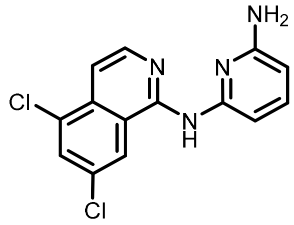

Pyridine-2,6-diamine was reacted with 1,5,7-trichloroisoquinoline to afford Cpd 7 (yield: 18%). HPLC-MS (Method A): Rt= 2.05 min (95%), $[M+H]^+$ m/z 305, 307. ^1H NMR (400 MHz, DMSO-d_6) δ 9.42 (s, 1H), 8.74 (s, 1H), 8.19 (d, *J* = 5.8 Hz, 1H), 8.03 (s, 1H), 7.38 (d, *J* = 5.5 Hz, 3H), 6.14 (s, 1H), 5.66 (s, 2H).

Compound 8 (N^2-(5-(trifluoromethyl)isoquinolin-1-yl)pyridine-2,6-diamine)

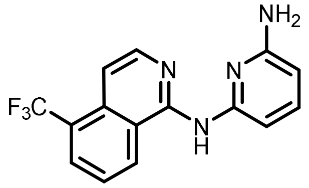

Pyridine-2,6-diamine was reacted with 1-chloro-5-(trifluoromethyl)isoquinoline to afford Cpd 8 (yield: 9%). HPLC-MS (Method A): Rt= 2.03 min (100%), $[M+H]^+$ m/z 305. ^1H NMR (400 MHz, CD_3OD) δ 8.62 (d, *J* = 8.5 Hz, 1H), 8.11 (d, *J* = 7.4 Hz, 1H), 7.71 (t, *J* = 8.0 Hz, 1H), 7.46 (td, *J* = 7.9, 5.2 Hz, 2H), 7.35 (s, 2H), 6.27 (d, *J* = 8.5 Hz, 1H).

Compound 13 (N^2-(5-(trifluoromethoxy)isoquinolin-1-yl)pyridine-2,6-diamine)

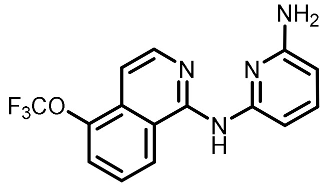

Pyridine-2,6-diamine was reacted with 1-chloro-5-(trifluoromethoxy)isoquinoline to afford Cpd 13 (yield: 22%). HPLC-MS (Method A): Rt= 2.05 min (96%), $[M+H]^+$ m/z 321. ^1H NMR (400 MHz, CD_3OD) δ 8.35 (d, *J* = 7.9 Hz, 1H), 8.09 (broad singlet, 1H), 7.67 (dt, *J* = 16.0, 8.0 Hz, 2H), 7.47 (t, *J* = 7.9 Hz, 2H), 7.41 – 7.26 (broad signal, 1H), 6.27 (d, *J* = 8.7 Hz, 1H).

Compound 14 (N^2-(7-methylisoquinolin-1-yl)pyridine-2,6-diamine)

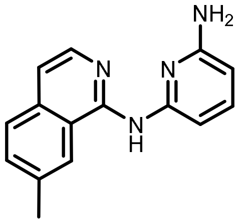

Pyridine-2,6-diamine was reacted with 1-chloro-7-methylisoquinoline to afford Cpd 14 (yield: 9%). HPLC-MS (Method A): Rt= 1.83 min (93%), $[M+H]^+$ m/z 251. ^{1}H NMR (400 MHz, CD_3OD): δ 8.09 (s, 1H), 7.90 (s, 1H), 7.70 (d, *J* = 8.3 Hz, 1H), 7.60 – 7.54 (m, 1H), 7.44 (t, *J* = 7.9 Hz, 1H), 7.33 (s, 1H), 7.16 (t, *J* = 7.9 Hz, 1H), 6.27 – 6.17 (m, 1H), 5.83 (d, *J* = 7.9 Hz, NH), 2.57 (s, 3H).

Compound 16 (N^2-(5-(trifluoromethyl)isoquinolin-1-yl)pyrazine-2,6-diamine)

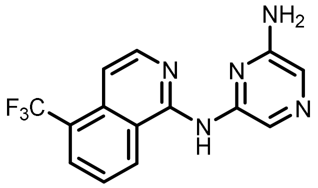

Pyrazine-2,6-diamine was reacted with 1-chloro-5-(trifluoromethyl)isoquinoline to afford Cpd 16 (yield: 34%). HPLC-MS (Method A): Rt= 1.82 min (98%), $[M+H]^+$ m/z 306. ^{1}H NMR (400 MHz, CD_3OD): δ 8.67 (br s, 2H), 8.11 (br s, 2H), 7.71 (br s, 2H), 7.52 (br s, 2H).

Compound 10 (N^2-(6-chloroisoquinolin-1-yl)pyridine-2,6-diamine)

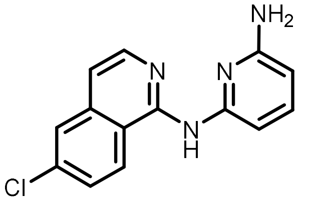

Pyridine-2,6-diamine was reacted with 1,6-dichloroisoquinoline to afford Cpd 10 (yield: 21%). HPLC-MS (Method A): Rt= 1.85 min (97%), $[M+H]^+$ m/z 271, 273. ^{1}H NMR (400 MHz, CD_3OD) δ 8.29 (d, *J* = 8.9 Hz, 1H), 7.94 (s, 1H), 7.79 (d, *J* = 1.6 Hz, 1H), 7.54 (dd, *J* = 9.0, 2.1 Hz, 1H), 7.43 (t, *J* = 7.9 Hz, 1H), 7.27 (s, 1H), 7.10 (s, 1H), 6.23 (d, *J* = 8.4 Hz, 1H).

Compound 11 (N^2-(5,6-dichloroisoquinolin-1-yl)pyridine-2,6-diamine)

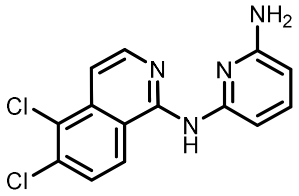

Pyridine-2,6-diamine was reacted with 1,5,6-trichloroisoquinoline to afford Cpd 11 (yield: 11%). HPLC-MS (Method A): Rt= 2.03 min (92%), $[M+H]^+$ m/z 305, 307. ^{1}H NMR (400 MHz, CD_3OD) δ 8.29 (d, *J* = 9.1 Hz, 1H), 8.10 (s, 1H), 7.69 (d, *J* = 9.0 Hz, 1H), 7.49 (s, 1H), 7.46 (t, *J* = 8.0 Hz, 1H), 7.29 (s, 1H), 6.26 (dd, *J* = 8.0, 0.7 Hz, 1H).

Compound 12 (N^2-(5-methoxyisoquinolin-1-yl)pyridine-2,6-diamine)

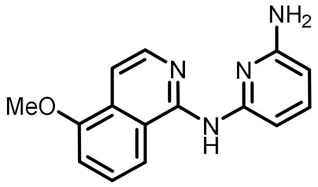

Pyridine-2,6-diamine was reacted with 1-chloro-5-methoxyisoquinoline to afford Cpd 12 (yield: 13%). HPLC-MS (Method A): Rt= 1.83 min (92%), $[M+H]^+$ m/z 267. ^{1}H NMR (400 MHz, CD_3OD) δ 7.97 (d, *J* = 5.7 Hz, 1H), 7.83 (d, *J* = 8.5 Hz, 1H), 7.55 (t, *J* = 8.4 Hz, 2H), 7.44 (t, *J* = 7.9 Hz, 1H), 7.34 – 7.24 (m, 1H), 7.19 (d, *J* = 7.8 Hz, 1H), 6.23 (d, *J* = 7.9 Hz, 1H), 4.01 (s, 3H).

Compound 20 (N^2-(5-methoxyisoquinolin-1-yl)pyrazine-2,6-diamine)

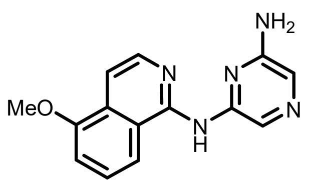

Pyrazine-2,6-diamine was reacted with 1-chloro-5-methoxyisoquinoline to afford Cpd 20 (yield: 15%). HPLC-MS (Method A): Rt= 1.67 min (97%), $[M+H]^+$ m/z 268. ^{1}H NMR (400 MHz, CD_3OD) δ 8.49 (s, 1H), 7.91 (s, 2H), 7.54 (dd, *J* = 18.3, 10.0 Hz, 3H), 7.19 (d, *J* = 7.8 Hz, 1H), 4.01 (s, 3H).

Compound 15 (1-((6-aminopyridin-2-yl)amino)isoquinoline-7-carbonitrile)

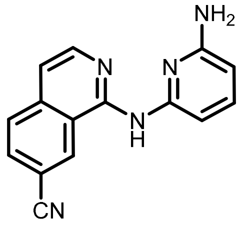

Pyridine-2,6-diamine was reacted with 1-chloroisoquinoline-7-carbonitrile to afford Cpd 15 (yield: 8%). Brown solid. HPLC-MS (Method A): Rt= 2.8 min (92%), $[M+H]^+$ m/z 262. ^1H NMR (400 MHz, DMSO): δ 9.5 (s, 1H), 9.21 (s, 1H), 8.20 (s, 1H), 7.98 (m, 2H), 7.48 (d, J =6.8 Hz, 1H), 7.39 (t, J = 8.0 Hz, 1H), 7.31 (s, 1H), 6.16 (d, J =7.6 Hz, 1H), 5.70 (s, 2H). Minor tautomer peaks were observed at δ 14.9, 8.85, 7.39 and 6.16.

Compound 22 (1-((6-aminopyrazin-2-yl)amino)isoquinoline-7-carbonitrile)

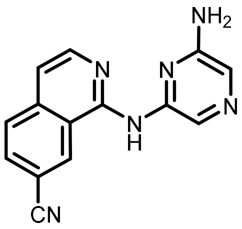

Pyrazine-2,6-diamine was reacted with 1-chloroisoquinoline-7-carbonitrile to afford Cpd 22 (yield: 3%). Brown solid. HPLC-MS (Method A): Rt= 4.04 min (96%), $[M+H]^+$ m/z 263. ^1H NMR (400 MHz, DMSO): δ 9.69 (s, 1H), 9.23 (s, 1H), 8.68 (s, 1H), 8.25 (d, J = 6.0 Hz, 1H), 8.03 (s, 1H), 7.58 (s, 1H), 7.39 (d, J = 5.6 Hz, 1H), 6.19 (s, 2H), 5.76 (s, 1H), 1H). Minor tautomer peaks were observed at δ 14.3, 8.89, 8.04, 7.80, 7.68, 7.49, 7.39, 6.73 and 6.42.

Compound 5 (N^2-(7-Chloroisoquinolin-1-yl)pyrazine-2,6-diamine)

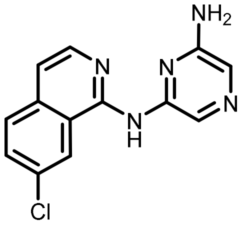

Pyrazine-2,6-diamine was reacted with 1,7-dichloroisoquinoline to afford Cpd 5 (yield: 24%). HPLC-MS (Method A): Rt= 1.62 min, $[M+H]^+$ m/z 272, 274. ^1H NMR

(400 MHz, DMSO-d6) *δ* 9.51 (s, 1H), 8.74 (s, 1H), 8.64 (s, 1H), 8.12 (d, *J* = 5.2 Hz, 1H), 7.91 (d, *J* = 9.2 Hz, 1H), 7.75 (d, *J* = 8.4 Hz, 1H), 7.54 (s, 1H), 7.35 (d, *J* = 5.2 Hz, 1H), 6.15 (s, 2H). Minor tautomer peaks were observed at *δ* 11.32, 8.56, 7.41, 6.71 and 6.38.

Compound 19 (*N*5-(5-chloroisoquinolin-1-yl)pyrimidine-2,5-diamine)

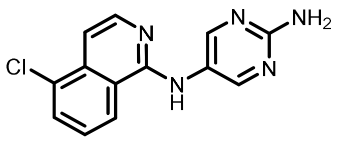

Pyrimidine-2,5-diamine was reacted with 1,7-dichloroisoquinoline to afford Cpd 19 (yield: 7%). Orange solid. HPLC-MS (Method A): Rt= 7.71 min (96%), $[M+H]^+$ m/z 272, 274.^{1}H NMR (400 MHz, DMSO-d6): *δ* 9.15 (s, 1H), 8.49 (s, 1H), 8.43 (d, *J* = 8.4 Hz, 1H), 8.00 (d, *J* = 6.0 Hz, 1H), 7.89 (d, *J* = 7.6 Hz, 1H), 7.58-7.62 (m, 1H), 7.26-7.27 (m, 1H), 6.50 (brs, 2H).

Synthesis of Compound 17 (*N*5-(5-chloroisoquinolin-1-yl)pyridine-2,5-diamine)

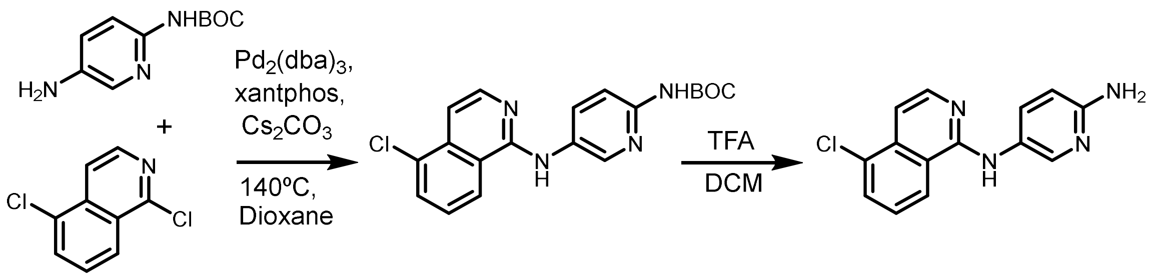

A mixture of 1,5-dichloroisoquinoline (1 eq.), *tert*-butyl(5-aminopyridin-2-yl)carbamate (1.5 eq), $Pd_2(dba)_3$ (0.1 eq.), XantPhos (0.2 eq.) and cesium carbonate (2 eq.) in dioxane (5.3 mL/mmol) (pre-degassed) was heated at 140ºC for 5h under nitrogen atmosphere. The mixture was filtered through a celite pad and concentrated under reduced pressure. The filtrate was extracted with ethyl acetate, washed with brine and dried with sodium sulphate. The residue was purified by flash column chromatography (DCM/MeOH) to obtain the desired product *tert*-butyl(5-((5-chloroisoquinolin-1-yl)amino)pyridin-2-yl)carbamate

which was used directly in the subsequent reaction without further purification (yield: quantitative). HPLC-MS (Method A): Rt= 2.48 min, $[M+H]^+$ m/z 371, 373.

Trifluoroacetic acid (TFA) (2 mL) was added to a solution of *tert*-butyl(5-((5-chloroisoquinolin-1-yl)amino)pyridin-2-yl)carbamate in DCM (2 mL). The solution was stirred at Rt for 5h and concentrated under vacuum. The resultant residue was purified by flash column chromatography (DCM/MeOH) to obtain the desired amine product *N*⁵-(5-chloroisoquinolin-1-yl)pyridine-2,5-diamine (yield: 33%). HPLC-MS (Method A): Rt= 1.60 min (99%), $[M+H]^+$ m/z 271, 273. ^{1}H NMR (400 MHz, CD_3OD) δ 8.26 (dt, *J* = 8.5, 0.9 Hz, 1H), 8.09 (d, *J* = 2.6 Hz, 1H), 7.90 (d, *J* = 6.1 Hz, 1H), 7.78 (dt, *J* = 8.9, 2.4 Hz, 1H), 7.71 (dd, *J* = 8.8, 2.6 Hz, 1H), 7.51 (dd, *J* = 8.4, 7.6 Hz, 1H), 7.35 (dd, *J* = 6.1, 0.9 Hz, 1H), 6.67 (dd, *J* = 8.8, 0.7 Hz, 1H).

The following compounds were synthesized following the same synthetic route as Cpd 17.

Compound 9 (N^2-(7-methoxyisoquinolin-1-yl)pyridine-2,6-diamine)

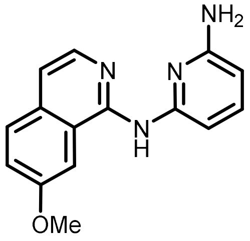

t*ert*-butyl(6-aminopyridin-2-yl)carbamate was reacted with 1-chloro-7-methoxyisoquinoline to afford tert-butyl (6-((7-methoxyisoquinolin-1-yl)amino)pyridin-2-yl)carbamate.

tert-butyl (6-((7-methoxyisoquinolin-1-yl)amino)pyridin-2-yl)carbamate was treated with TFA to obtain Cpd 9 (yield: 45%). Yellow solid. HPLC-MS (Method B): Rt= 1.86 min (98%), $[M+H]^+$ m/z 267. ^{1}H NMR (400 MHz, CD_3OD) δ 7.97 (brs, 1H), 7.87-7.85 (m, 2H), 7.54 (t, J = 8.0 Hz, 2H), 7.37 (d, J = 6.4 Hz, 1H), 6.89 (d, J = 8.0 Hz, 1H), 6.34 (d, J = 8.0 Hz, 1H), 4.02 (s, 3H).

Compound 21 (1-((2-aminopyrimidin-5-yl)amino)isoquinoline-7-carbonitrile)

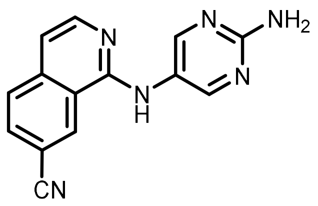

Tert-butyl(5-aminopyrimidin-2-yl)carbamate was reacted with 1-chloroisoquinoline-7-carbonitrile to afford 1-(2-amino-5-pyrimidinylamino)-7-isoquinolinecarbonitrile (yield: 58%).

1-(2-amino-5-pyrimidinylamino)-7-isoquinolinecarbonitrile was treated with TFA to obtain Cpd 21 (yield: 12%). Pale green solid. HPLC-MS (Method A): Rt= 8.57 min (98%), $[M+H]^+$ m/z 263.^{1}H NMR (400 MHz, DMSO-d_6): *δ* 9.26 (brs, 1H), 9.03 (s, 1H), 8.53 (s, 2H), 8.05 (d, *J* = 6.0 Hz, 1H), 7.98 (m, 2H), 7.17-7.18 (m, 1H), 6.50 (brs, 2H).

Compound 23 (N^4-(7-Chloroisoquinolin-1-yl)pyridine-2,4-diamine).

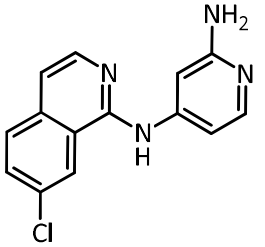

Tert-butyl(4-aminopyridin-2-yl)carbamate was reacted with 1,7-dichloroisoquinoline to afford *tert*-butyl(4-((7-chloroisoquinolin-1-yl)amino)pyridin-2-yl)carbamate.(used in the next step without purification).

Tert-butyl(4-((7-chloroisoquinolin-1-yl)amino)pyridin-2-yl)carbamate was treated with TFA to obtain Cpd 23 (yield: 48%, two steps). HPLC-MS (Method A): Rt= 1.83 min (93.2%), $[M+H]^+$ m/z 270, 272. ^{1}H NMR (400 MHz, DMSO-d_6) δ 10.11 (s, 1H), 8.66 (d, *J* = 1.8 Hz, 1H), 8.26 (d, *J* = 5.7 Hz, 1H), 8.04 (d, *J* = 8.8 Hz, 1H), 7.88 – 7.83 (m, 1H), 7.79 (d, *J* = 7.2 Hz, 1H), 7.73 (d, *J* = 2.1 Hz, 1H), 7.62 (s, 2H), 7.59 (d, *J* = 5.8 Hz, 1H), 7.12 (dt, *J* = 5.6, 2.8 Hz, 1H).

Full ^{1}H NMR and LC-MS spectra for all compounds discussed in the main text are provided in **File S2**.
