# Supplementary material for: Development of Small-Molecule Allosteric Modulators of Beta-Galactosidase (β-Gal) for the Treatment of GM1 Gangliosidosis and Morquio B
Source: Int J Mol Sci. 2026 Apr 18;27(8):3631. doi: 10.3390/ijms27083631 (PMC13115887; doi:10.3390/ijms27083631)
Supplement: Supplementary file 1 [file ijms-27-03631-s001.zip › File S1.pdf]

File S1. Effect of STAR Compounds on β-Galactosidase Activity in Human Fibroblast Lysates (Biochemical Assays).

The ability of the selected compounds to affect the enzymatic activity of GLB1 was investigated in biochemical assays performed in cell lysates from human wild-type (WT) fibroblasts using 4-methylumbelliferyl-β-D-galactopyranoside (4-MU-β-D-Gal) as substrate in citrate buffer pH 4. Under these conditions, the pharmacological chaperone NN-DGJ inhibited endogenous β-Gal activity in a dose-dependent manner, as expected for a competitive inhibitor. In contrast, the original hit compounds identified using the computational platform (Hit 3 and Hit 5) did not display inhibition of β-Gal activity at any of the concentrations tested. Similarly, Cpd 4 and its analogue Cpd 23, as representative members of the chemical series, did not inhibit the enzyme and showed a neutral effect on activity over the tested concentration range (Figure S2). These exploratory data support a non-inhibitory pharmacological chaperone profile for these compounds under the assay conditions used.

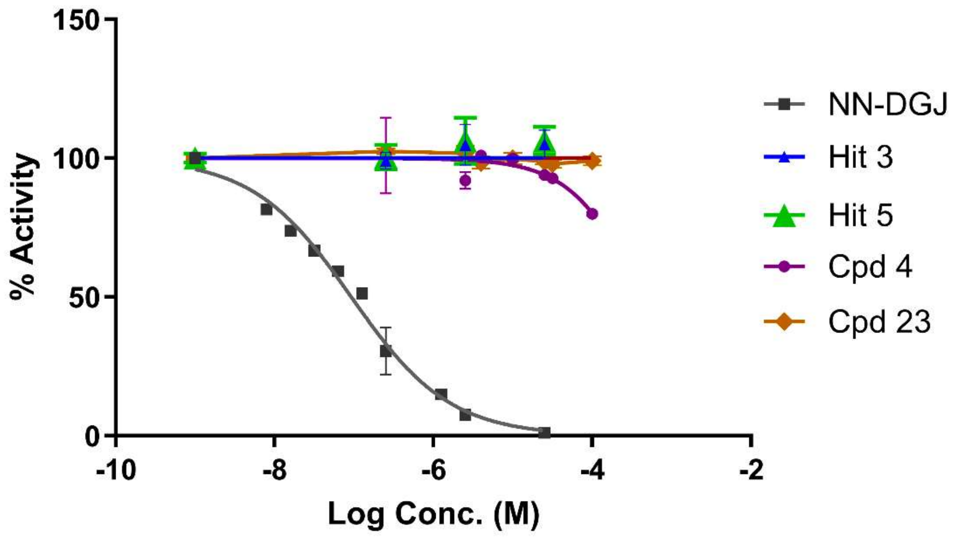

Figure S1. Effect of NN-DGJ and selected STAR compounds on β-galactosidase activity in lysates from WT β-Gal-expressing human fibroblasts. NN-DGJ was used as a reference competitive pharmacological chaperone and showed a clear dose-dependent inhibition of endogenous β-Gal activity, whereas Hit 3, Hit 5, Cpd 4 and Cpd 23 did not inhibit the enzyme over the tested concentration range.

Cpd 23 is described in the **Experimental Procedure**, even though it was not tested in the ICC assay due to compound shortage.

Materials and Methods

Enzyme inhibition assay

Lysates were prepared from wild-type β-galactosidase-expressing human fibroblasts using a lysis buffer containing 0.9% NaCl and 0.01% Triton X-100. Protein concentration was measured with a BCA protein assay kit, and samples were normalized to a final concentration of 0.5 mg/mL. The lysates (final concentration: 81.7 μg/mL) were subsequently incubated with the designated compound.

The enzymatic assay was performed in a reaction buffer comprising 0.20 mM 4-MU-β-D-Gal (4-MU) in 0.1 M citrate buffer (pH 4.0), supplemented with 100 mM NaCl and 0.02% NaN_3. Reactions were incubated at 37 °C for 30 minutes.

To stop the reaction, 140 μL of 200 mM glycine-NaOH buffer (pH 10.7) was added. Fluorescence from the released 4-MU was measured using a GloMax® Discover microplate reader, with excitation at 340 nm and emission at 460 nm.
