# Supplementary material for: Development of Small-Molecule Allosteric Modulators of Beta-Galactosidase (β-Gal) for the Treatment of GM1 Gangliosidosis and Morquio B
Source: Int J Mol Sci. 2026 Apr 18;27(8):3631. doi: 10.3390/ijms27083631 (PMC13115887; doi:10.3390/ijms27083631)

File S2. Characterization of synthesized compounds

Compound 1 (N^1-(7-chloroisoquinolin-1-yl)benzene-1,3-diamine)

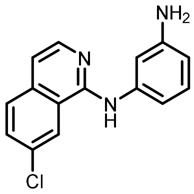

- ^{1}H NMR

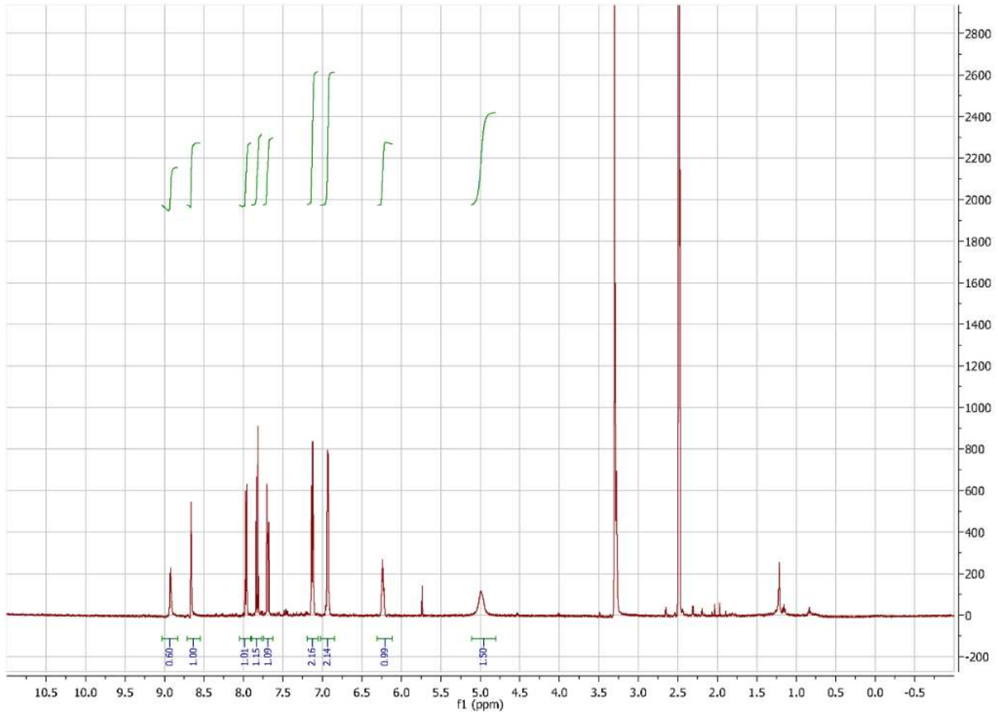

- HPLC-MS

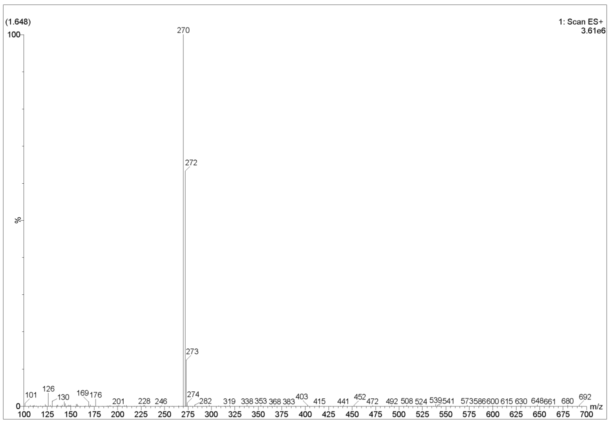

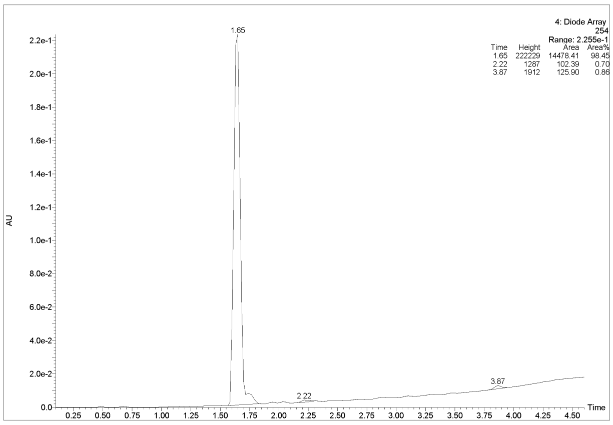

Compound 2 (*N*[1]-(5-chloroisoquinolin-1-yl)benzene-1,3-diamine)

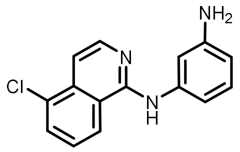

- ^{1}H NMR

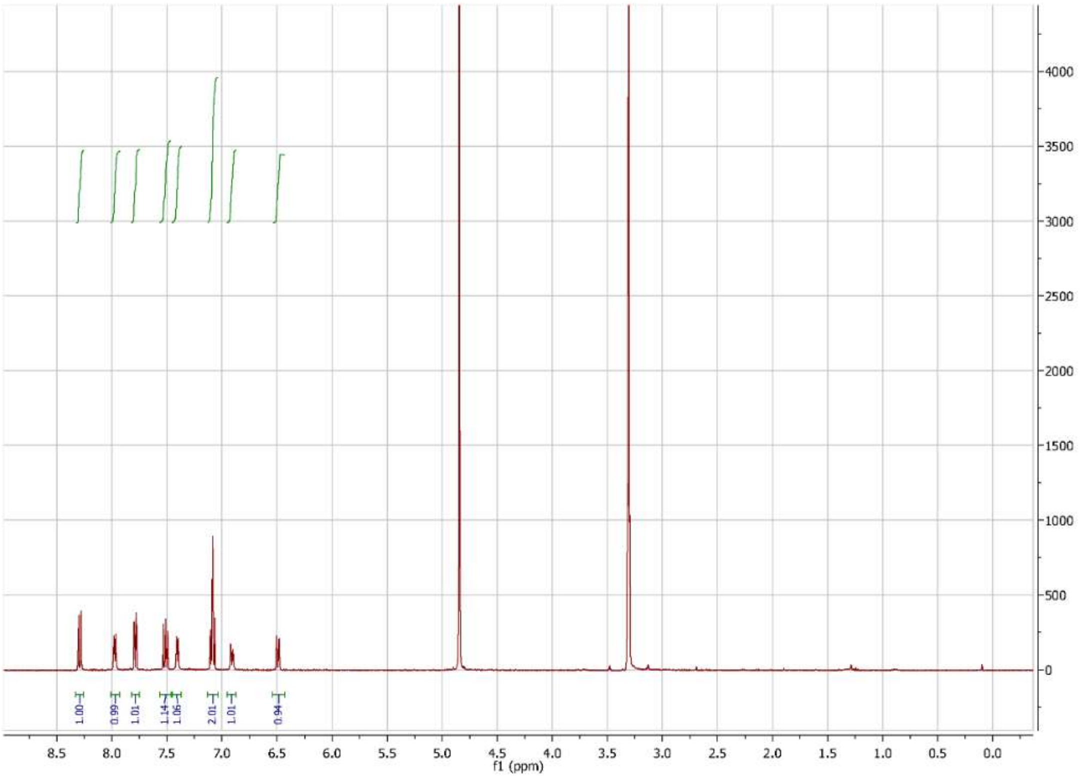

- HPLC-MS

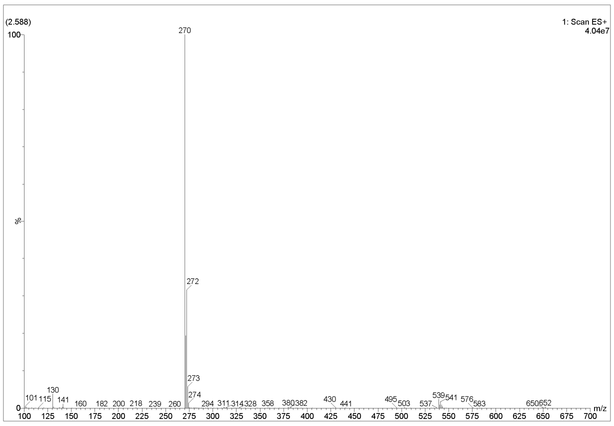

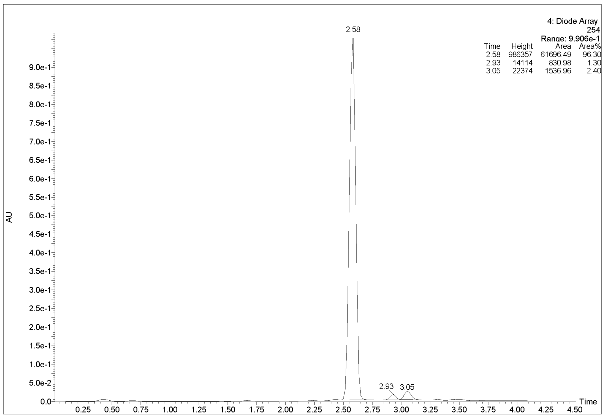

Compound 3 (N^1-(7-chloroisoquinolin-1-yl)benzene-1,4-diamine)

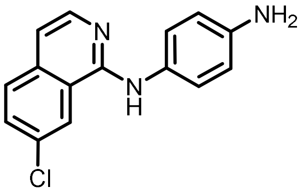

- ^{1}H NMR

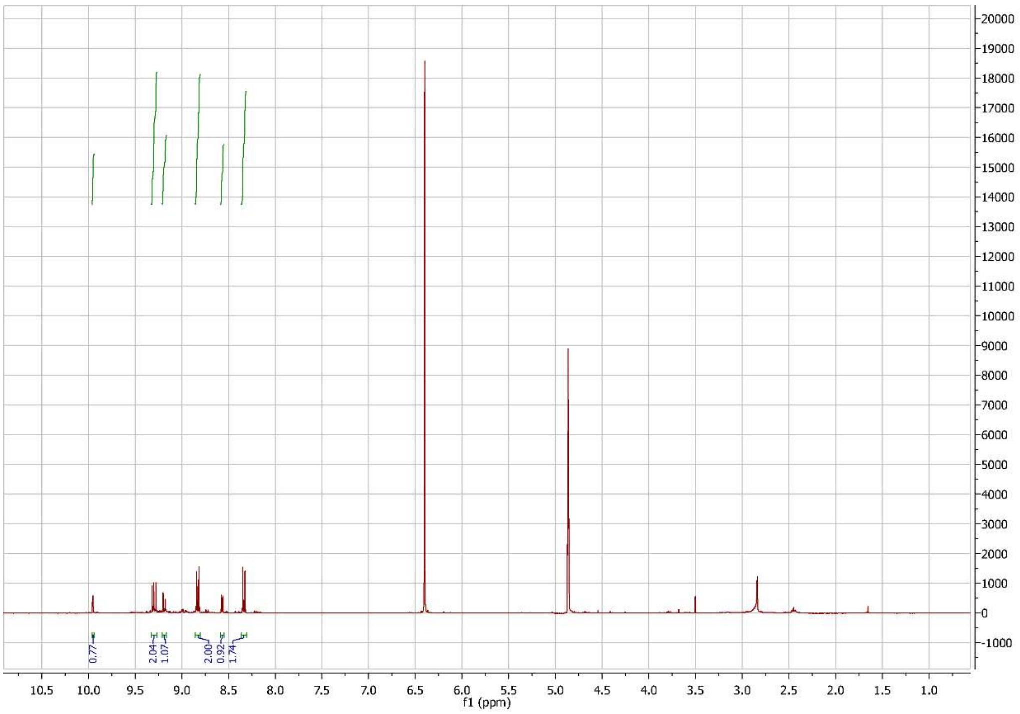

- HPLC-MS

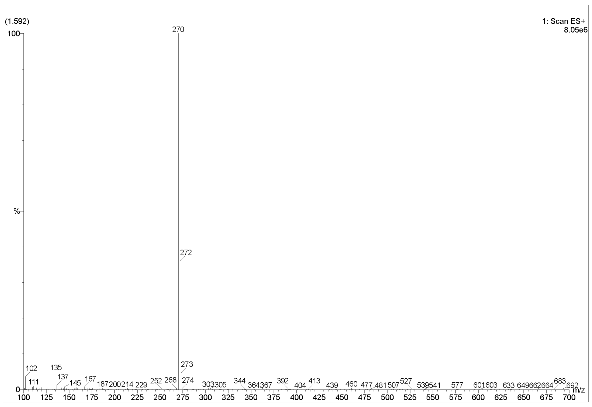

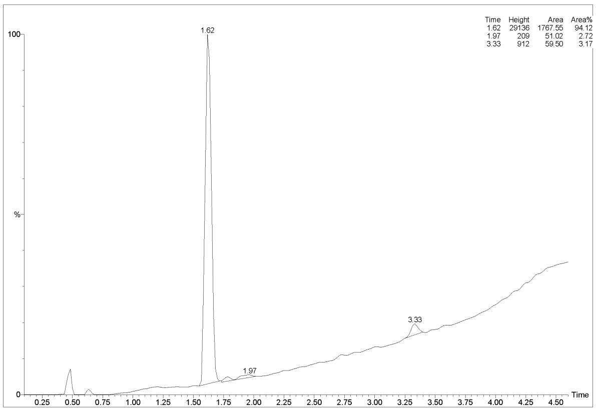

Compound 4 (*N*[2]-(7-chloroisoquinolin-1-yl)pyridine-2,6-diamine)

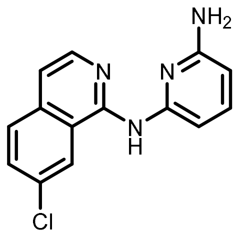

- ^{1}H NMR

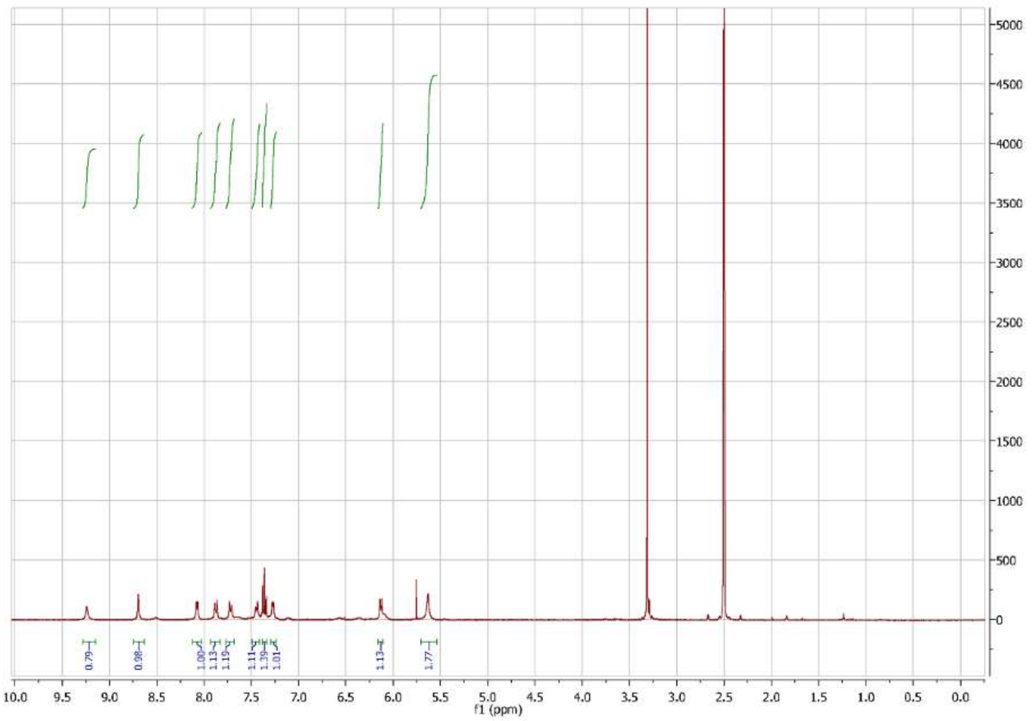

- HPLC-MS

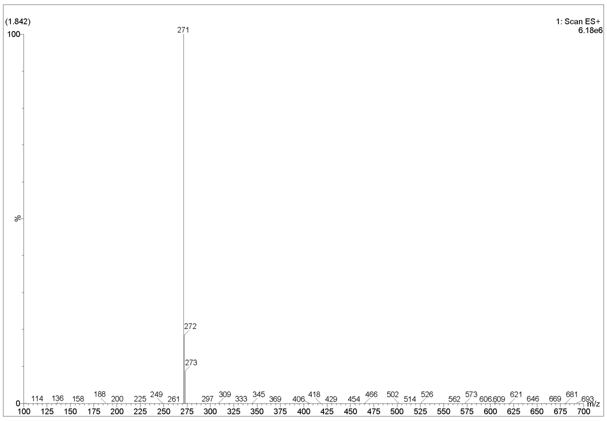

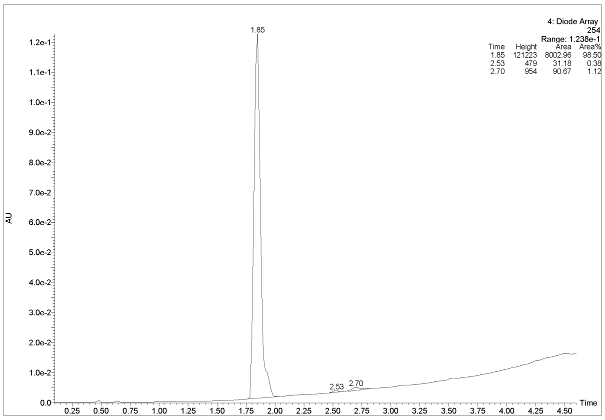

Compound 5 (N^2-(7-Chloroisoquinolin-1-yl)pyrazine-2,6-diamine)

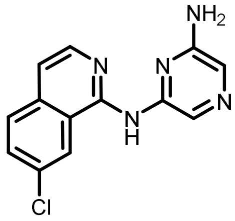

- ^{1}H NMR

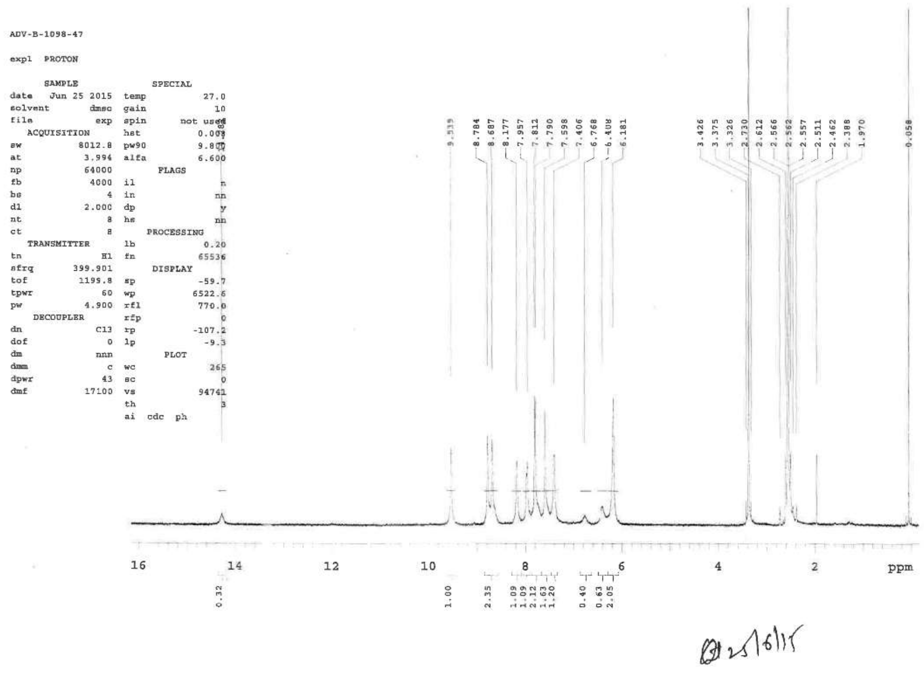

- HPLC-MS

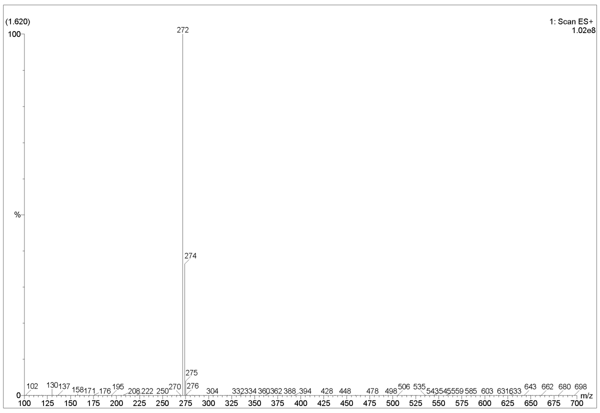

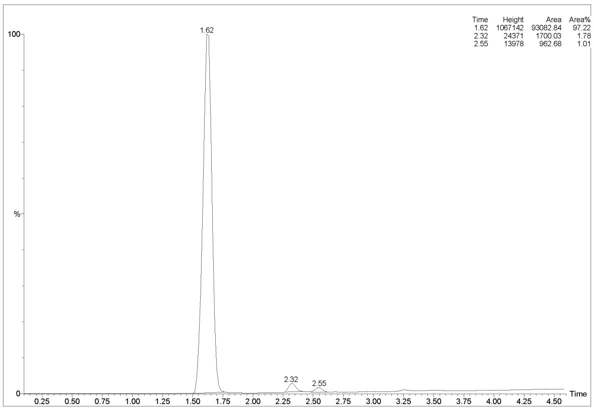

Compound 6 (N^2-(5-chloroisoquinolin-1-yl)pyridine-2,6-diamine)

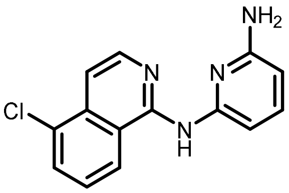

- ^{1}H NMR

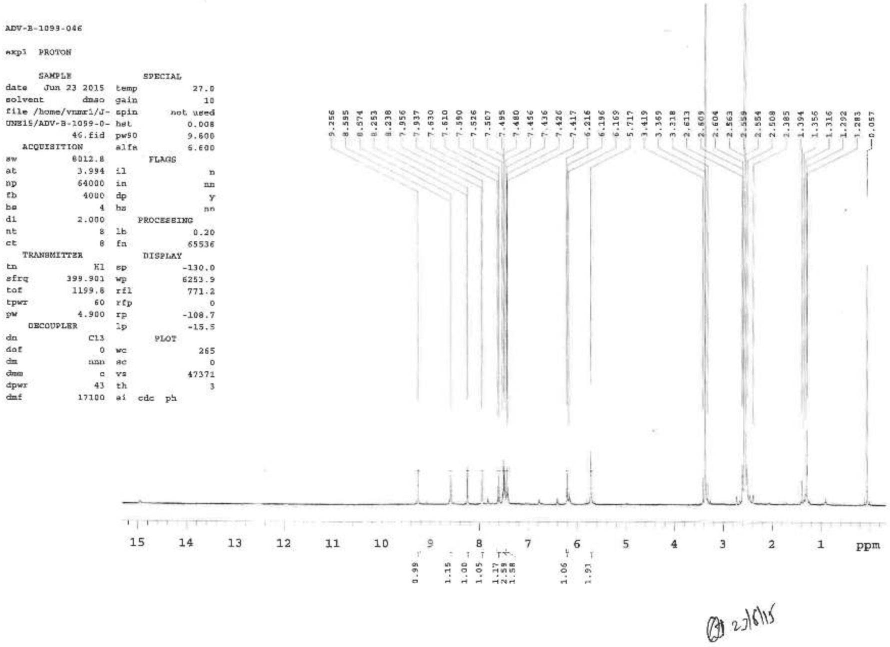

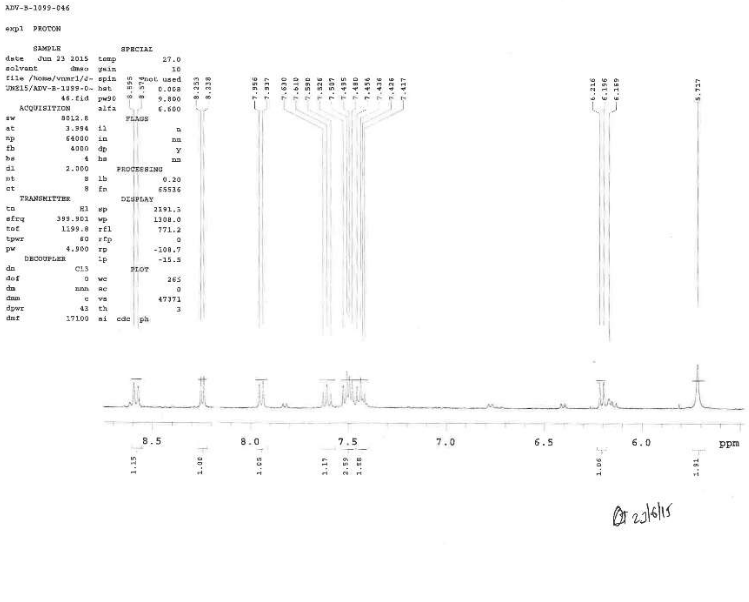

Compound 6 (N^2-(5-chloroisoquinolin-1-yl)pyridine-2,6-diamine)

- HPLC-MS

Injection Date : 6/22/2015 Seq Line :8.00
Sample Name : ADV-B-1099-46 Location :Vial 25
Acq Operator : Sai Injection Volume :10.00 ul
Injection time : 3:10:26 PM
Acq.Method : D:\data\JUNE 2015\220615 2015-06-22 14-08-53\FA_595+VE210NM.M
Data file : D:\DATA\JUNE 2015\220615 2015-06-22 14-08-53\PAE-1194-15.D

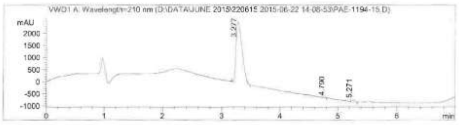

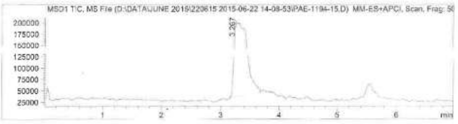

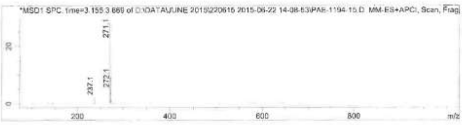

VWD1 A, Wavelength=210 nm

Peak #	Compound Name	Meas. Ret. T Min	Area	Area %
1		3.28	18341.5	97.40
2		4.79	47.5	0.25
3		5.27	442.1	2.35

MSD1 TIC, MS File

Peak #	Compound Name	Meas. Ret. T Min	Area	Area %
1		3.27	2534420.0	100.00

Data file :D:\BACK UP CHEM32\1\DATA\JUNE 2015\220615 2015-06-22 14-55-51\PAE-1194-15.D
Injection Date : 6/22/2015 Seq Line : 2
Sample Name : ADV-B-1099-46 Location : Vial 3
Acq Operator : Sai Inj. No. : 1
Injection Time : 3:32:15 PM Inj. Vol. : 10 µL
Sample info :

Analysis Method :D:\Back Up Chem32\1\DATA\JUNE 2015\220615 2015-06-22 14-55-51\SYM_9010KA.M

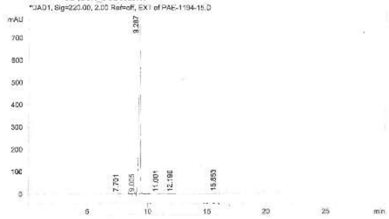

Signal 1: DAD1, Sig=220.00, 2.00 Ref=off, EXT

Peak #	RT [min]	Name	Area	Area %
1	7.701		9.7	0.148
2	8.581		11.4	0.175
3	9.005		2.8	0.043
4	9.287		6454.1	98.792
5	9.586		4.3	0.066
6	11.001		3.6	0.055
7	12.190		10.9	0.166
8	15.853		36.3	0.555

PAE-HPLC-001 Page 1 of 1

Compound 7 (N^2-(5,7-Dichloroisoquinolin-1-yl)pyridine-2,6-diamine)

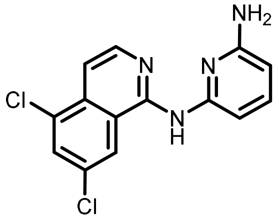

- ^{1}H NMR

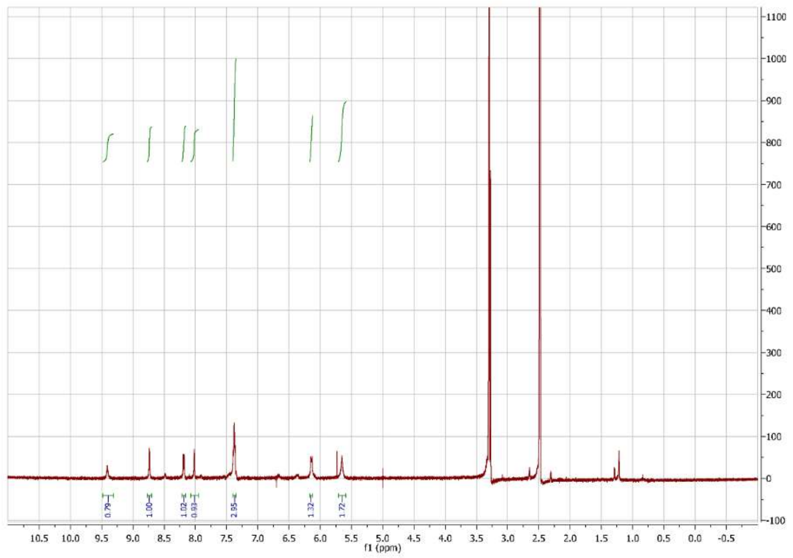

- HPLC-MS

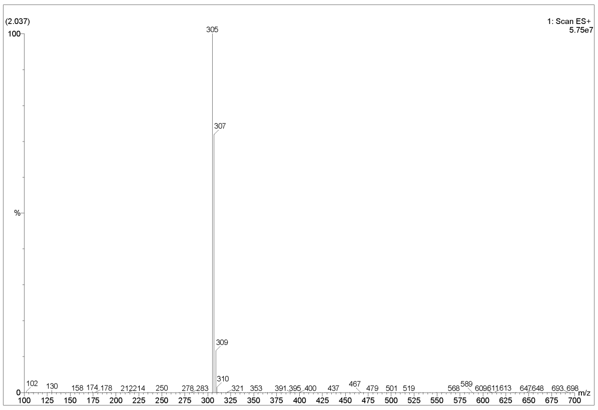

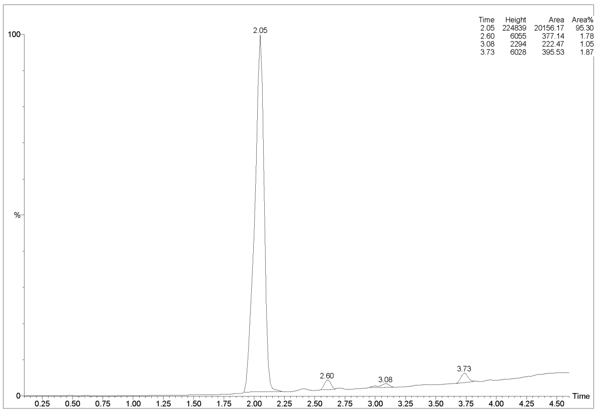

Compound 8 (N^2-(5-(trifluoromethyl)isoquinolin-1-yl)pyridine-2,6-diamine)

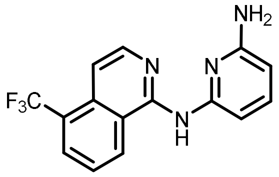

- ^{1}H NMR

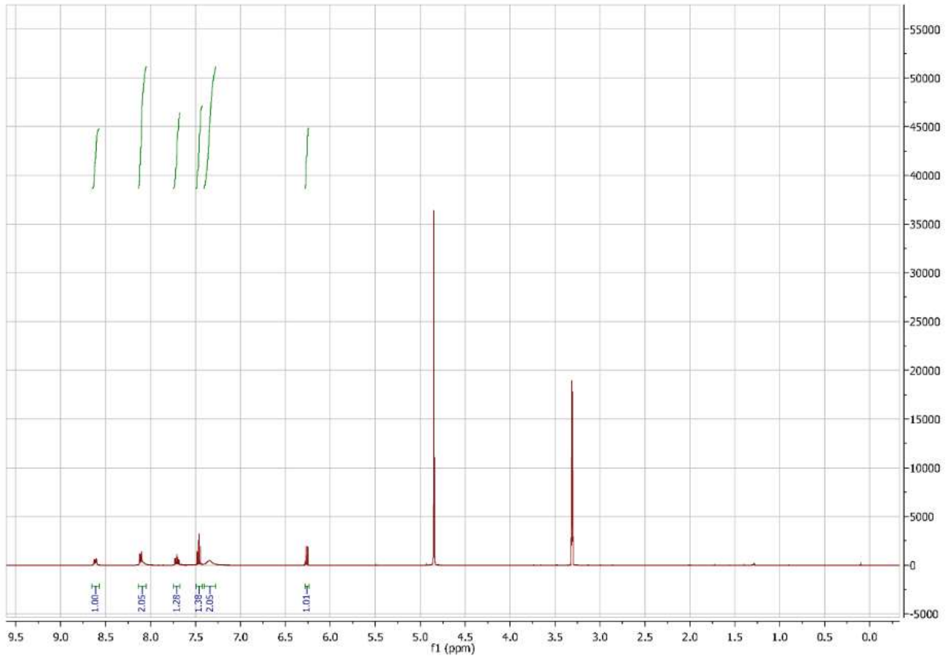

- HPLC-MS

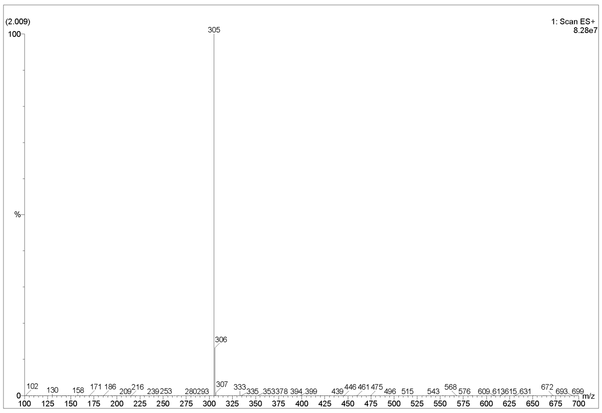

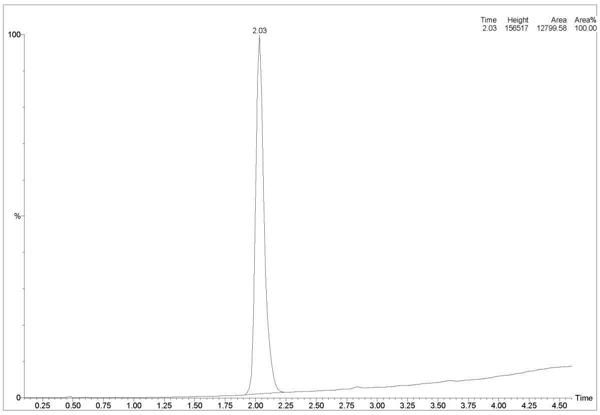

Compound 9 (N^2-(7-methoxyisoquinolin-1-yl)pyridine-2,6-diamine)

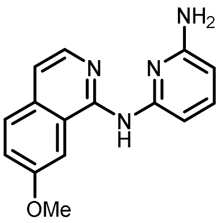

- ^{1}H NMR

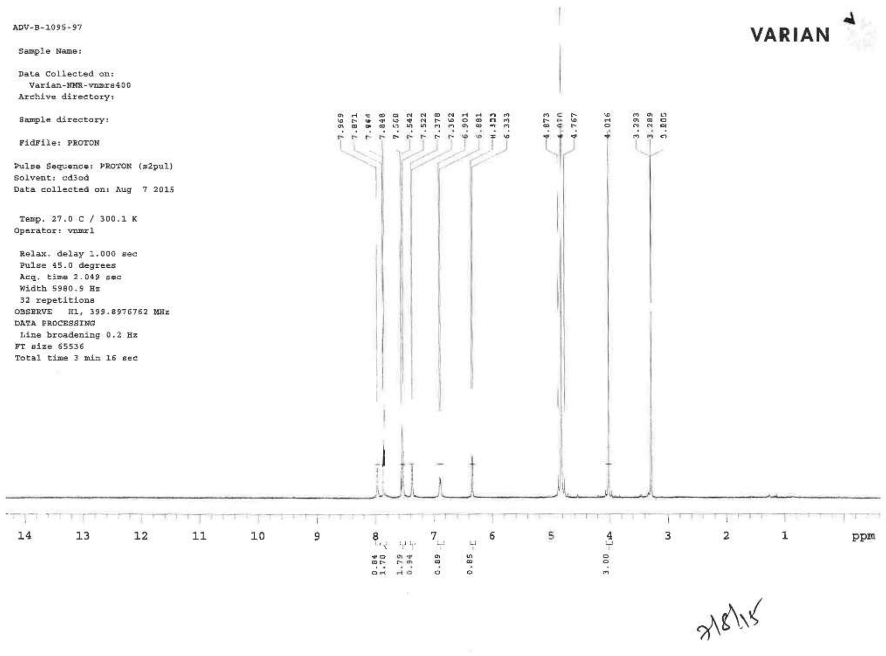

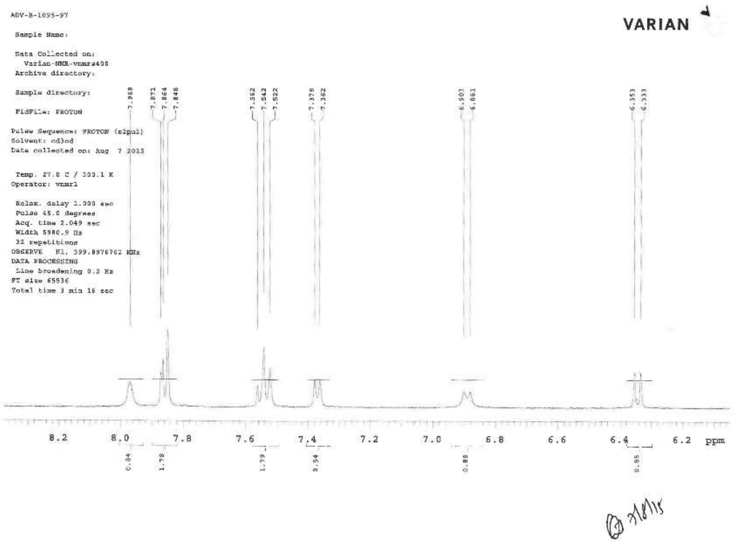

Compound 9 (N^2-(7-methoxyisoquinolin-1-yl)pyridine-2,6-diamine)

- HPLC-MS

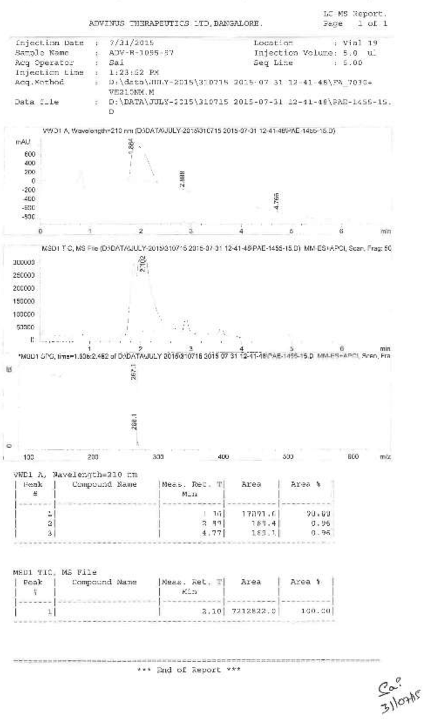

LC MS Report.
ADVINUS THERAPEUTICS LTD,BANGALORE. Page 1 of 1

Injection Date : 7/31/2015 Location : Vial 19
Sample Name : ADV-R-1055-57 Injection Volume: 5.0 ul
Acq Operator : Sai Seq Line : 5.00
Injection time : 1:23:22 PM
Acq.Method : D:\data\JULY-2015\310715 2015-07-31 12-41-48\FA_7030+VE210NM.M
Data file : D:\DATA\JULY-2015\310715 2015-07-31 12-41-48\PAE-1455-15.D

VWD1 A, Wavelength=210 nm

Peak #	Compound Name	Meas. Ret. T Min	Area	Area %
1		[illegible]	17091.6	98.09
2		[illegible]	169.4	0.96
3		4.77	163.1	0.96

MSD1 TIC, MS File

Peak #	Compound Name	Meas. Ret. T Min	Area	Area %
1		2.10	7212822.0	100.00

*** End of Report ***

Sai 31/07/15

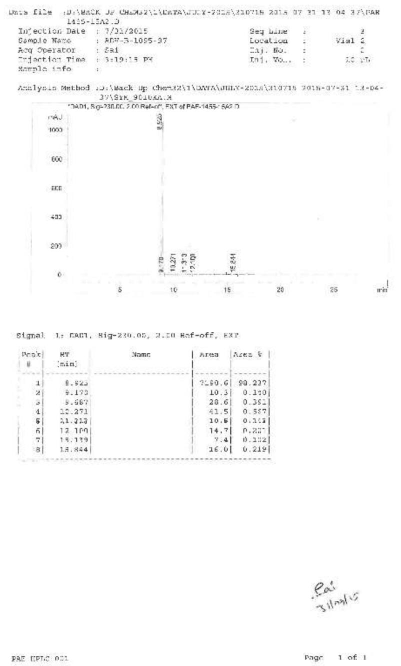

Data file : D:\BACK UP CHEM32\1\DATA\JULY-2015\310715 2015 07 31 13 04 37\PAE 1455-15A2.D
Injection Date : 7/31/2015 Seq Line : 2
Sample Name : ADV-R-1055-57 Location : Vial 2
Acq Operator : Sai Inj. No. : [illegible]
Injection Time : 3:19:15 PM Inj. Vo... : [illegible]
Sample info :

Analysis Method : D:\Back Up Chem32\1\DATA\JULY-2015\310715 2015-07-31 13-04-37\SYR_9010KA.M

Signal 1: DAD1, Sig=230.00, 2.00 Ref=off, EXT

Peak #	RT [min]	Name	Area	Area %
1	8.923		7190.6	98.237
2	9.173		10.3	0.140
3	9.667		28.6	0.391
4	10.271		41.5	0.567
5	11.213		10.6	0.145
6	12.100		14.7	0.201
7	15.139		7.4	0.102
8	15.844		16.0	0.219

Sai 31/07/15

PAE HPLC 001 Page 1 of 1

Compound 10 (*N*2-(6-chloroisoquinolin-1-yl)pyridine-2,6-diamine)

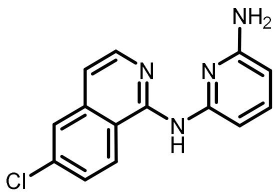

- 1H NMR

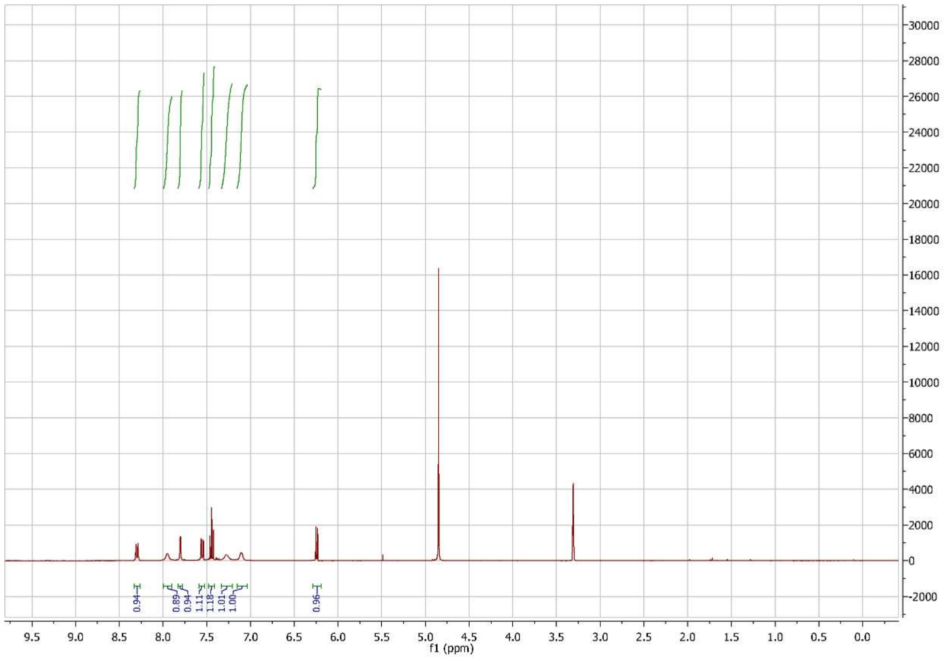

- HPLC-MS

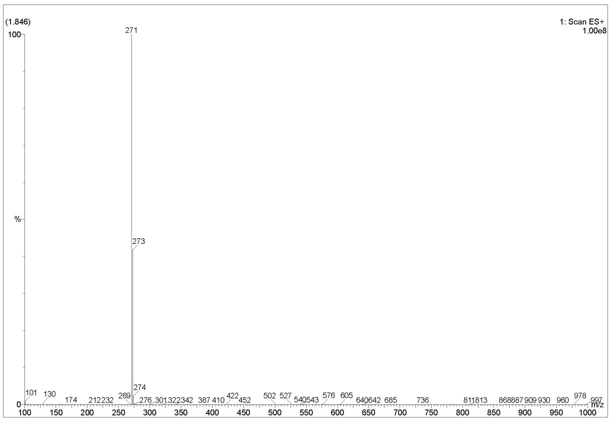

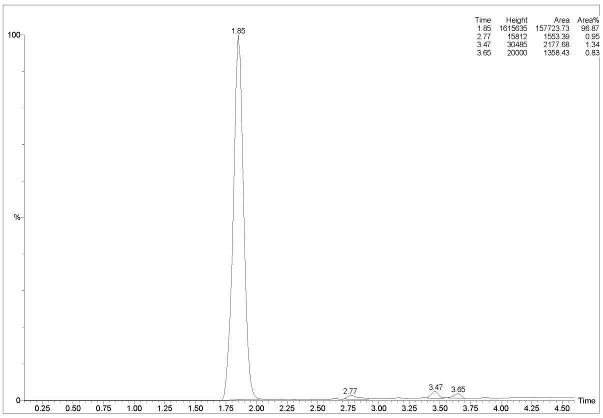

Compound 11 (*N*[2]-(5,6-dichloroisoquinolin-1-yl)pyridine-2,6-diamine)

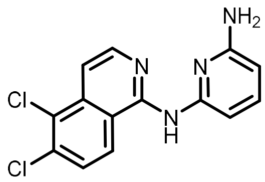

- ^{1}H NMR

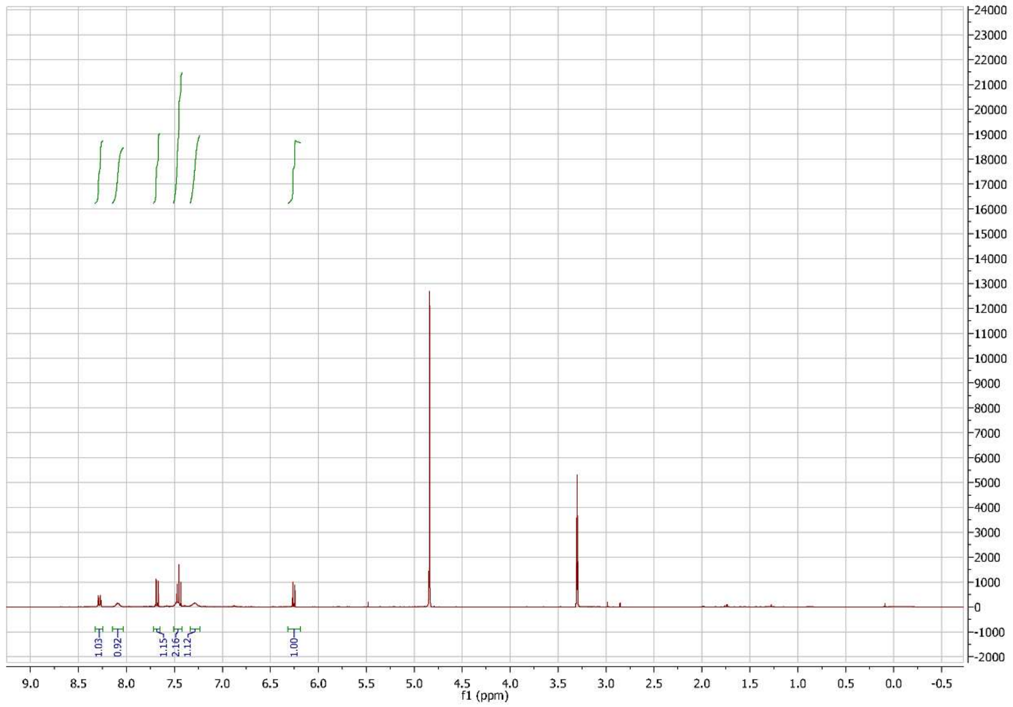

- HPLC-MS

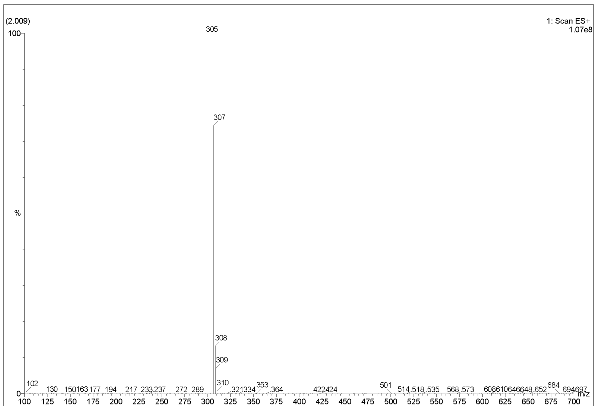

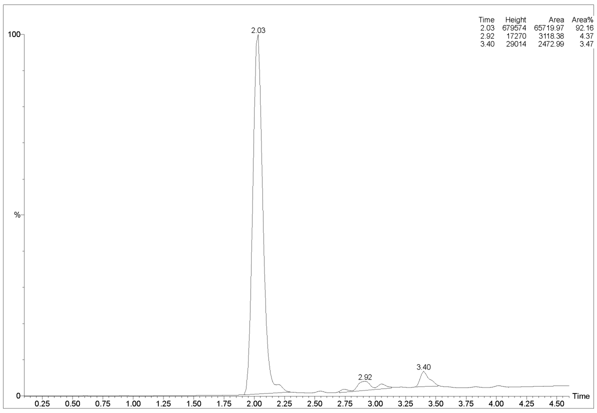

Compound 12 (*N*²-(5-methoxyisoquinolin-1-yl)pyridine-2,6-diamine)

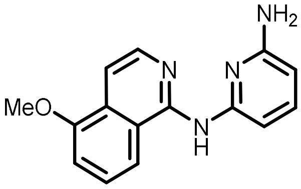

- ^{1}H NMR

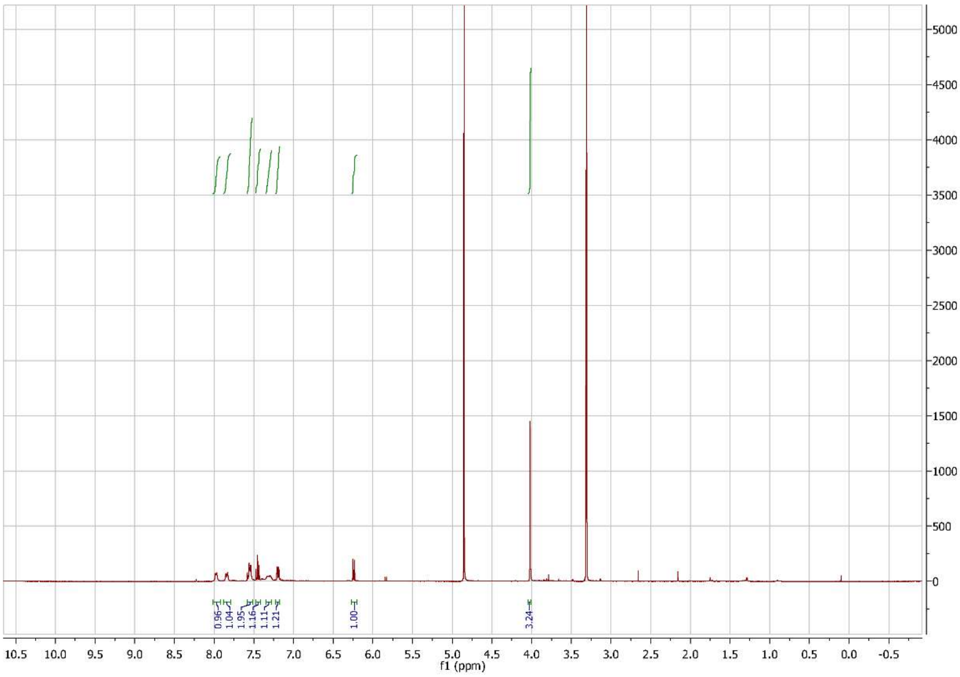

- HPLC-MS

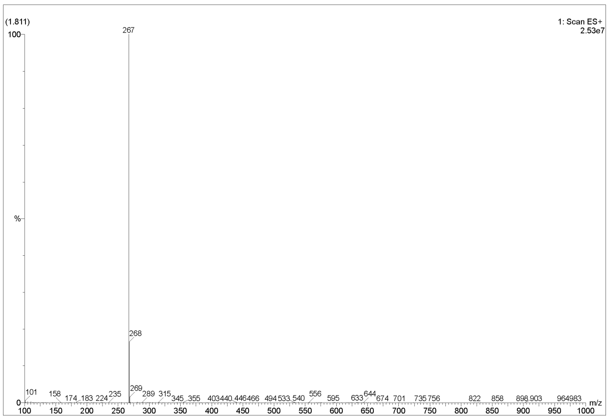

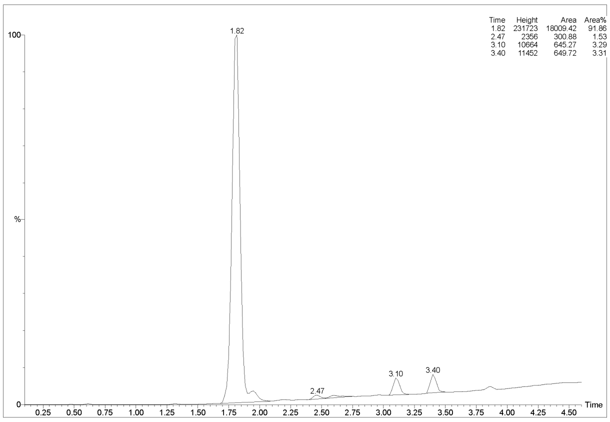

Compound 13 (*N*²-(5-(trifluoromethoxy)isoquinolin-1-yl)pyridine-2,6-diamine)

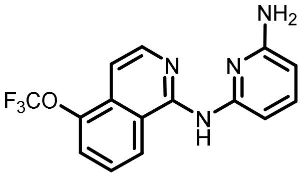

- ^{1}H NMR

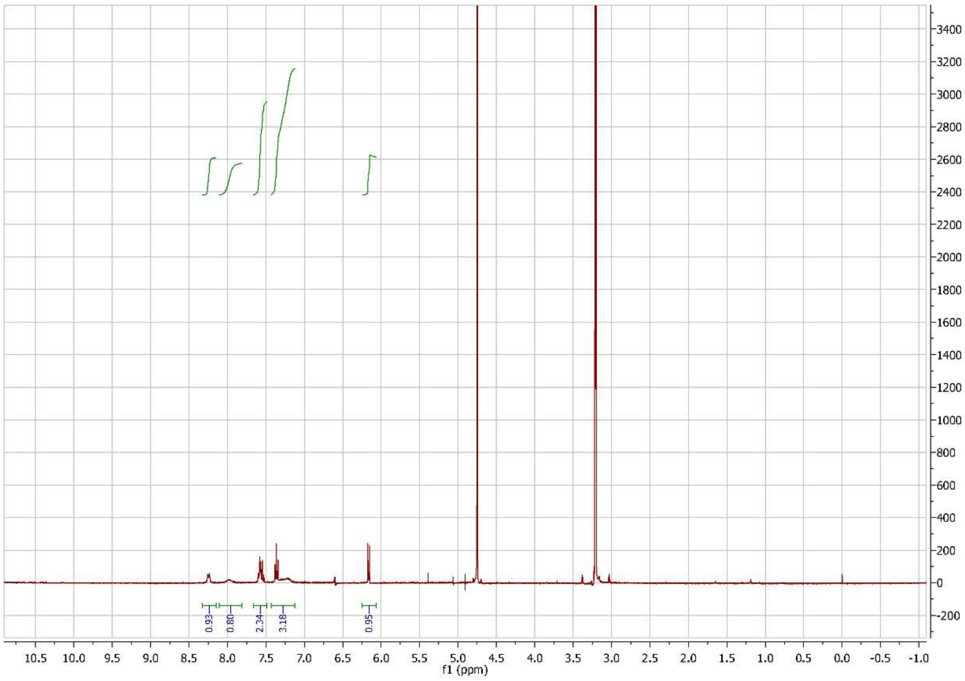

- HPLC-MS

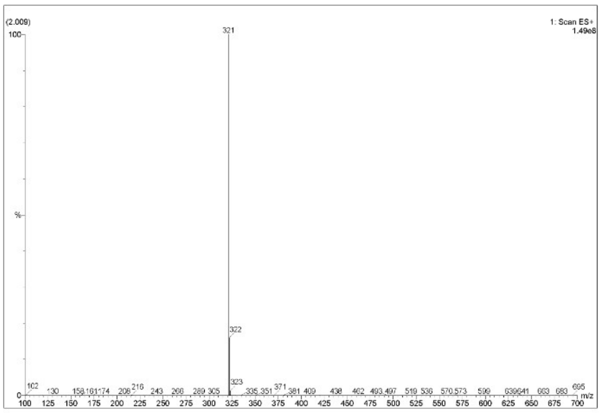

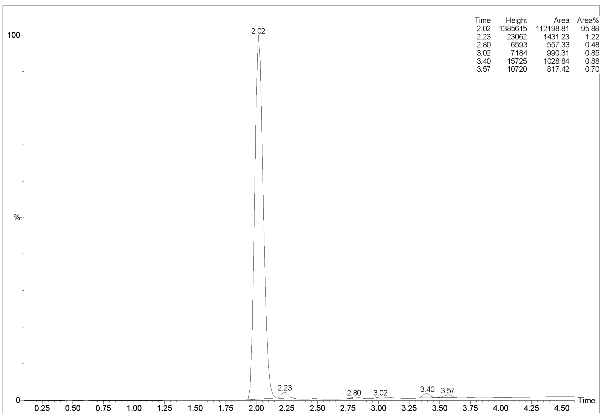

Time	Height	Area	Area%
2.02	1385615	112198.81	95.88
2.23	23062	1431.23	1.22
2.80	6593	557.33	0.48
3.02	7184	990.31	0.85
3.40	15725	1028.84	0.88
3.57	10720	817.42	0.70

Compound 14 (*N*[2]-(7-methylisoquinolin-1-yl)pyridine-2,6-diamine)

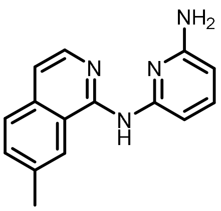

- ^{1}H NMR

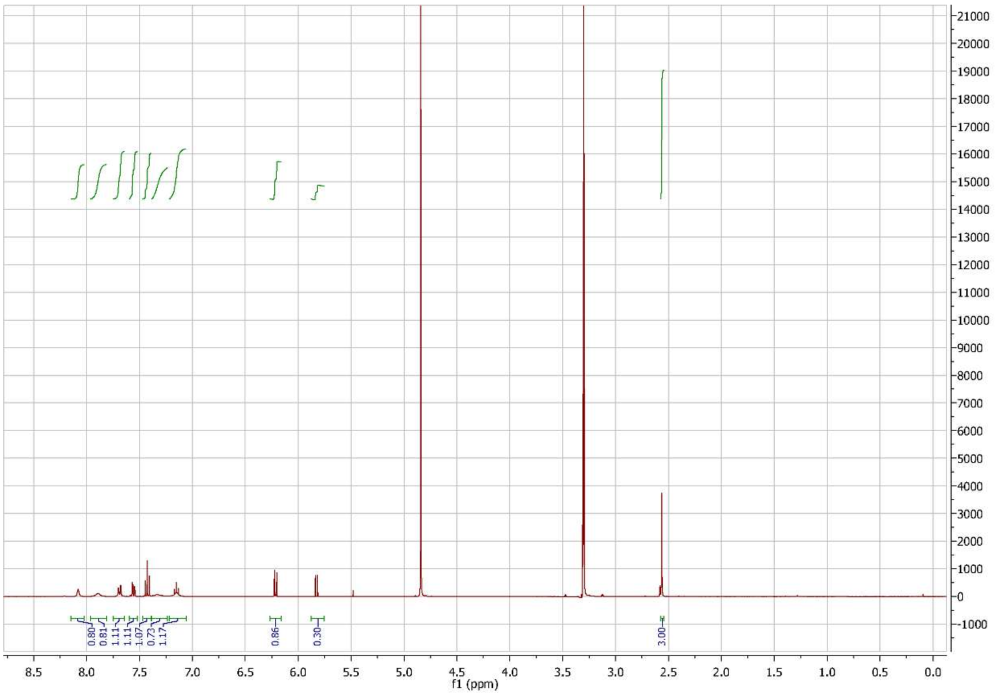

- HPLC-MS.

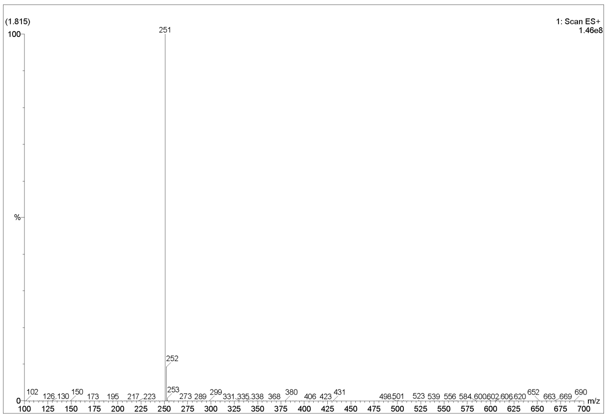

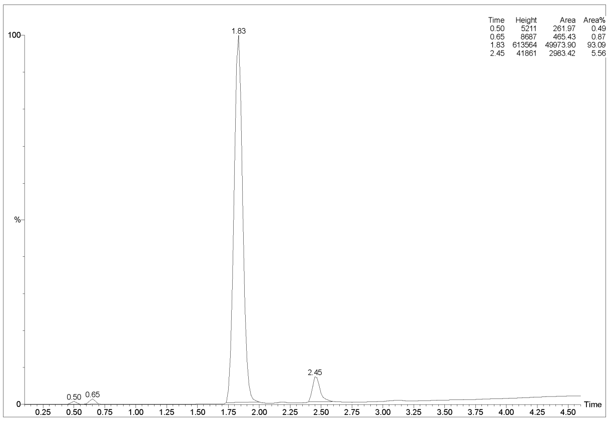

Compound 15 (1-((6-aminopyridin-2-yl)amino)isoquinoline-7-carbonitrile)

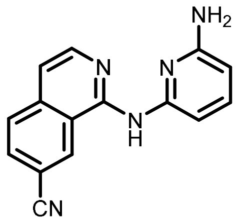

- ^{1}H NMR

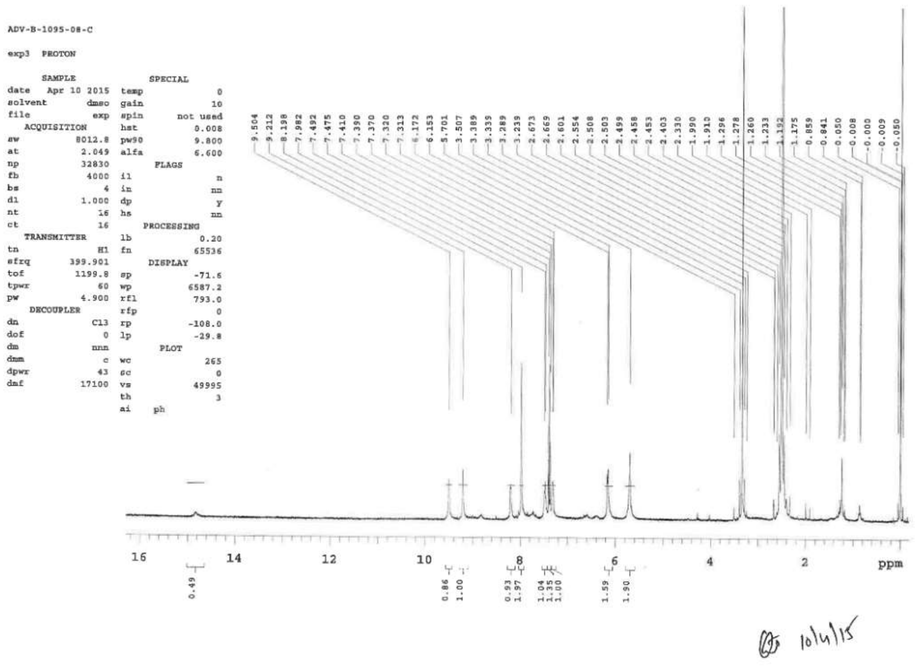

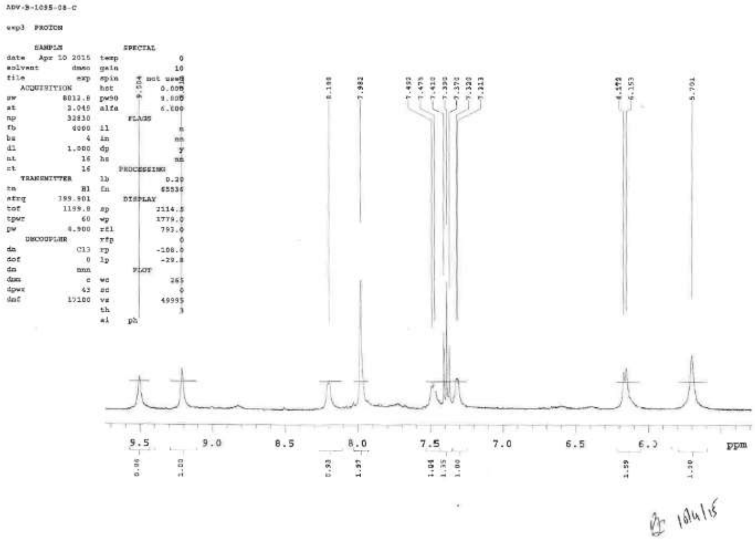

Compound 15 (1-((6-aminopyridin-2-yl)amino)isoquinoline-7-carbonitrile)

- HPLC-MS

```
Injection Date   :  4/10/2015                        Seq Line         :1.00
Sample Name      :  ADV-B-1095-08-C                  Location         :Vial 26
Acq Operator     :  Sai                              Injection Volume :5.00   ul
Injection time   :  1:17:53 PM
Acq.Method       :  D:\data\APRIL 2015\100415 2015-04-10 13-14-41\FA_595+
                    VE210NM.M
Data file        :  D:\DATA\APRIL 2015\100415 2015-04-10 13-14-41\PAE-0649-
                    15.D
```

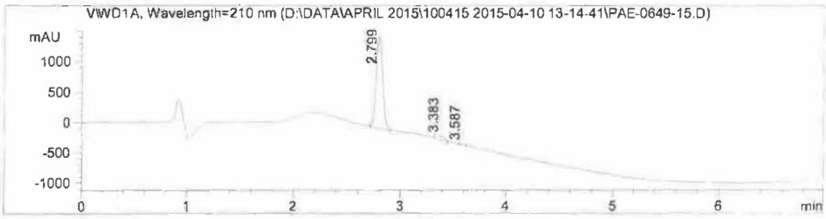

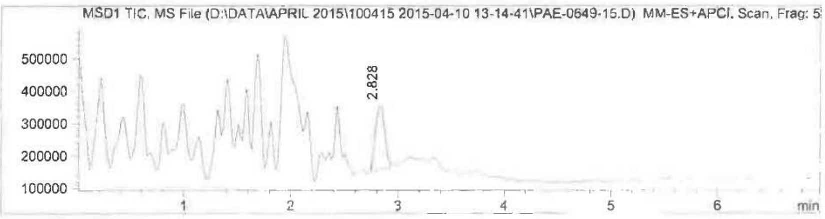

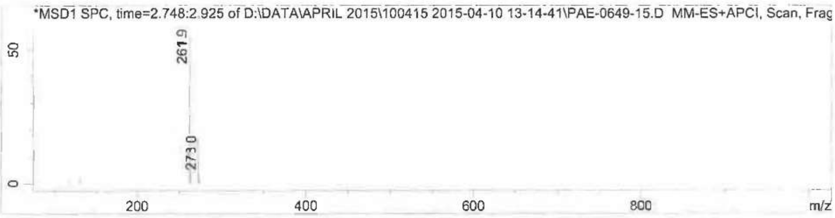

VWD1 A, Wavelength=210 nm

Peak #	Compound Name	Meas. Ret. T Min	Area	Area %
1		2.80	6509.3	94.73
2		3.38	301.9	4.39
3		3.59	60.4	0.88

MSD1 TIC, MS File

Peak #	Compound Name	Meas. Ret. T Min	Area	Area %
1		2.83	11069[illegible]16.0	100.00

10/4/15

Compound 16 (*N*²-(5-(trifluoromethyl)isoquinolin-1-yl)pyrazine-2,6-diamine)

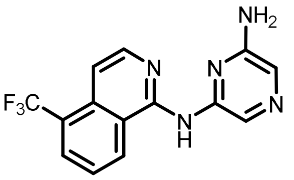

- ¹H NMR

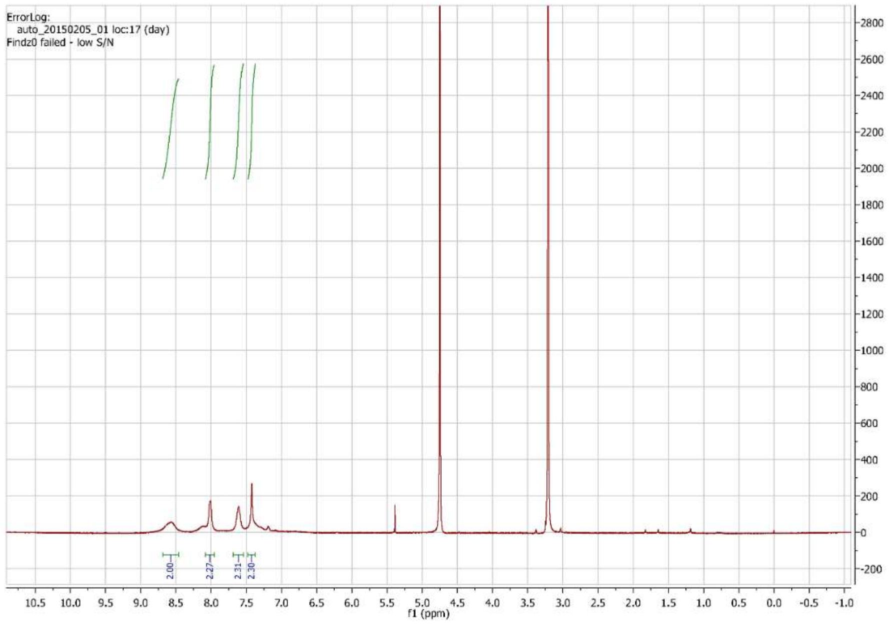

- HPLC-MS

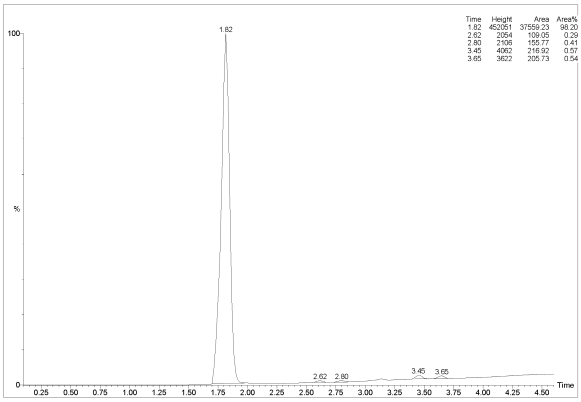

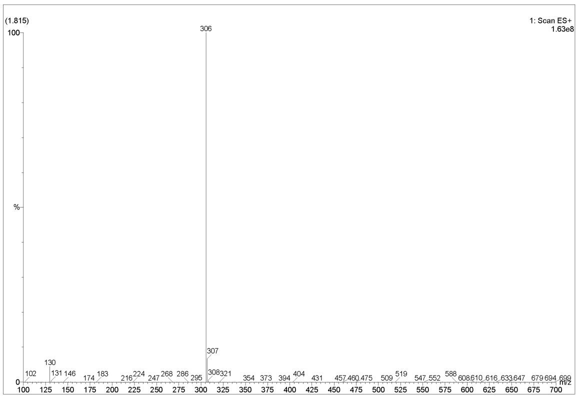

Compound 17 (*N*5-(5-chloroisoquinolin-1-yl)pyridine-2,5-diamine)

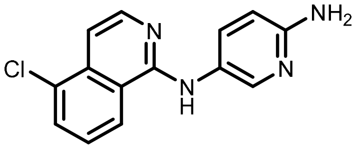

- ^{1}H NMR

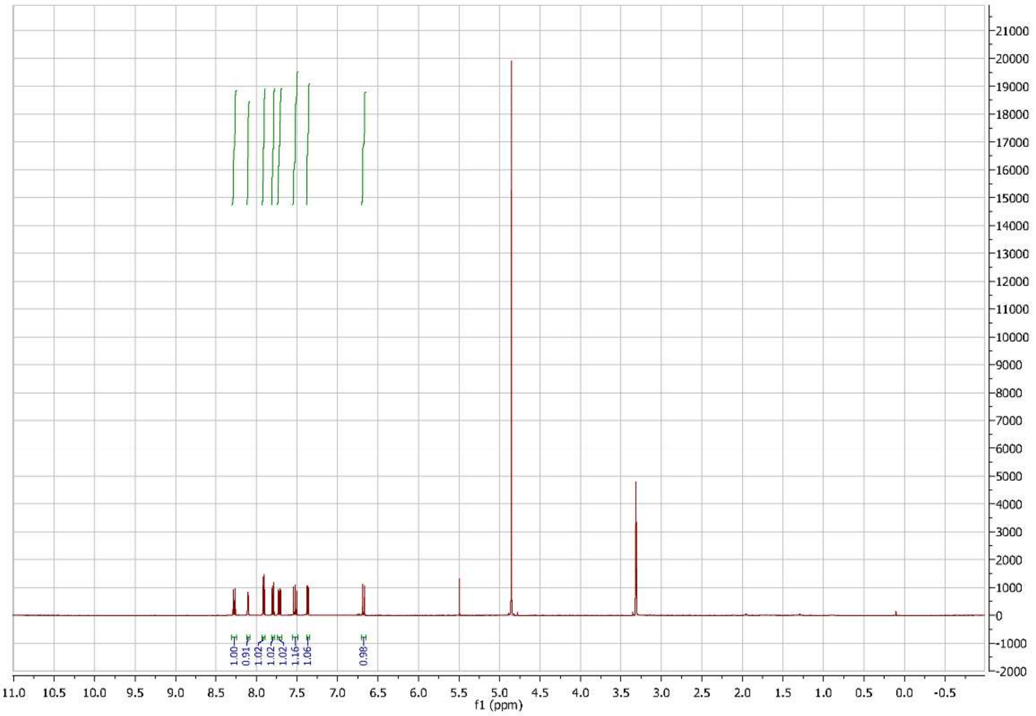

- HPLC-MS

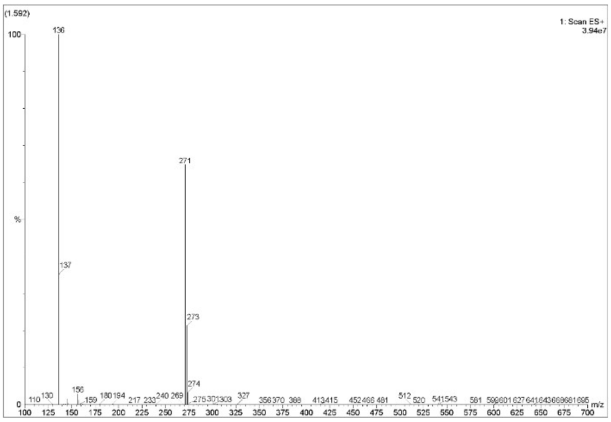

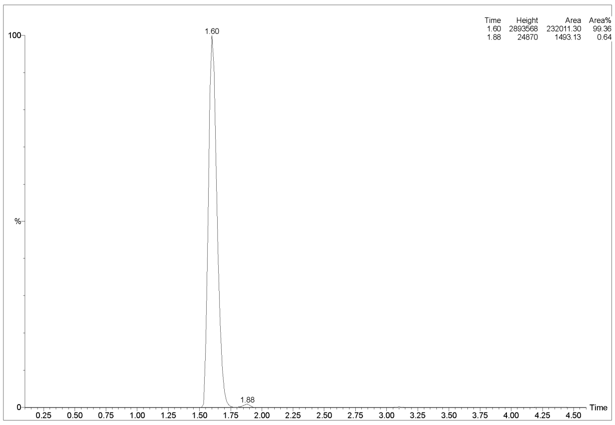

Compound 18 (*N*2-(5-chloroisoquinolin-1-yl)pyridine-2,5-diamine)

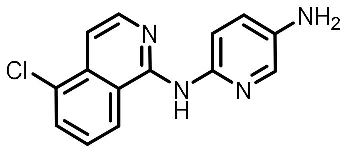

- ^{1}H NMR

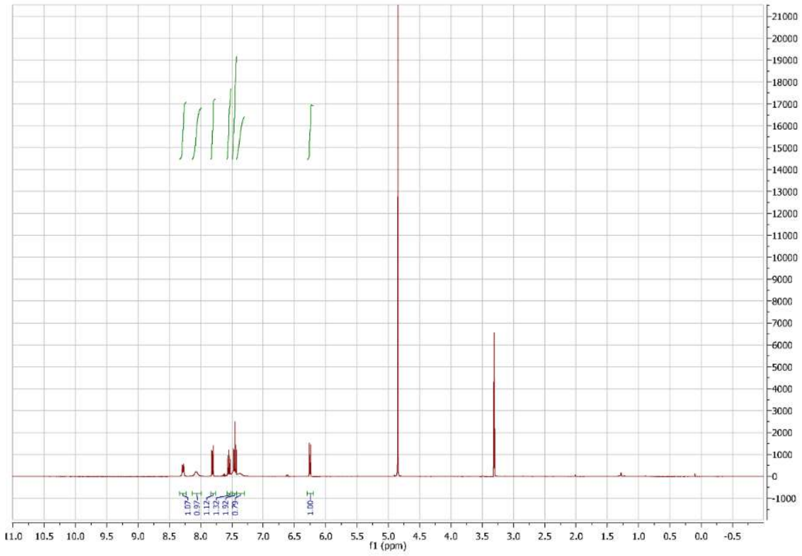

- HPLC-MS

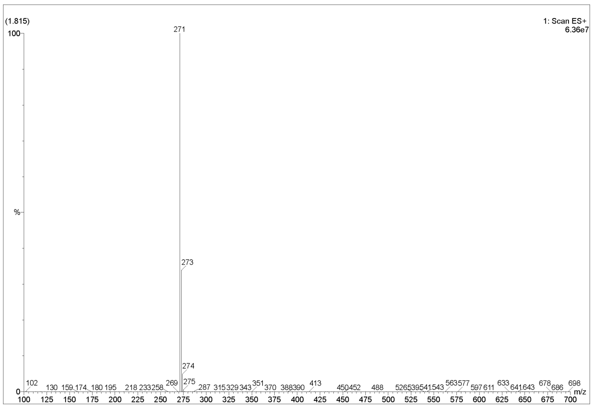

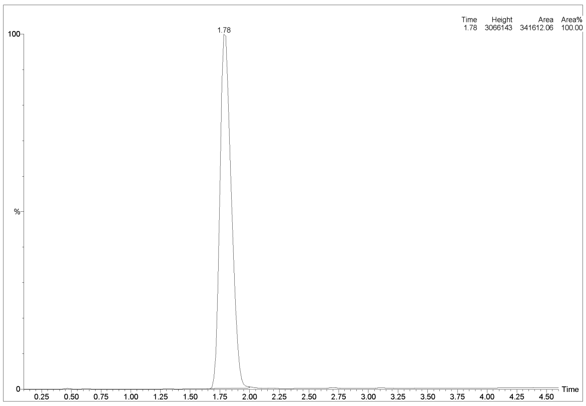

Compound 19 (N^5-(5-chloroisoquinolin-1-yl)pyrimidine-2,5-diamine)

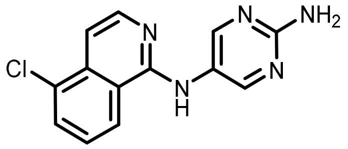

- ^{1}H NMR

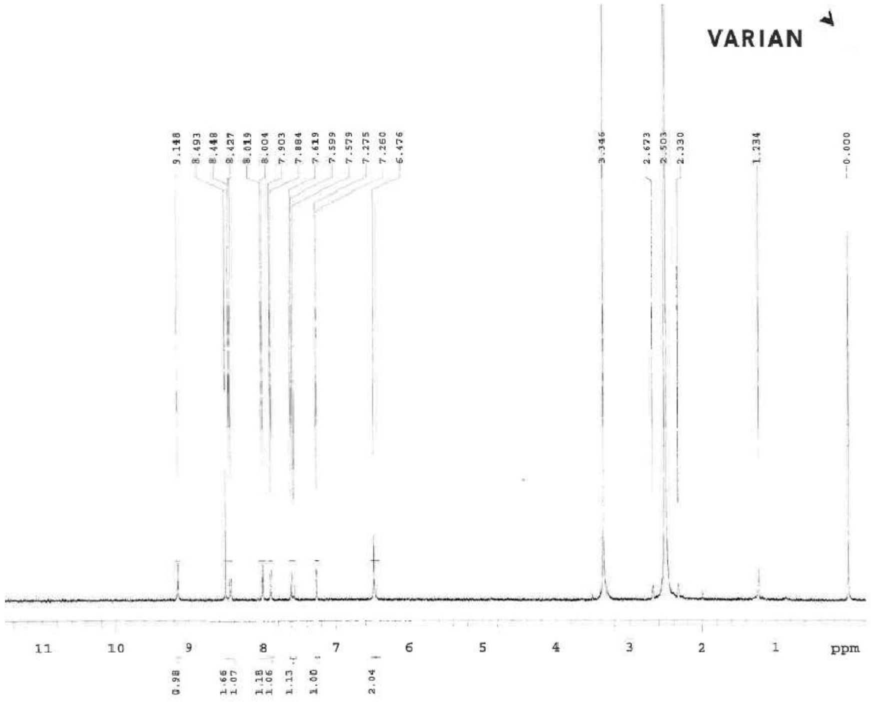

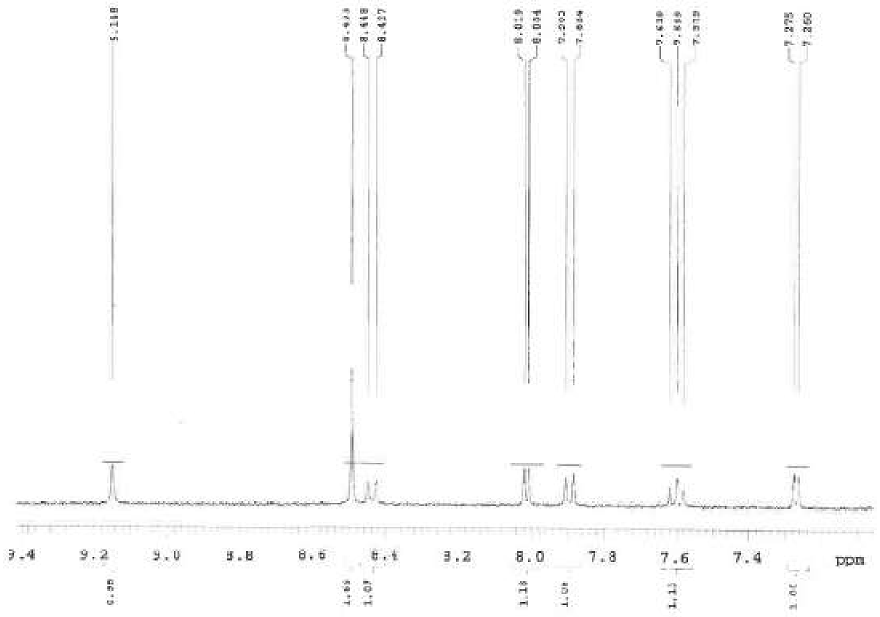

Compound 19 (N^5-(5-chloroisoquinolin-1-yl)pyrimidine-2,5-diamine)

- HPLC-MS

Analysis Method :D:\Back Up Chem32\1\DATA\FEB 2016\010216 2016-02-01 13-16-17\SYM_9010KA.M

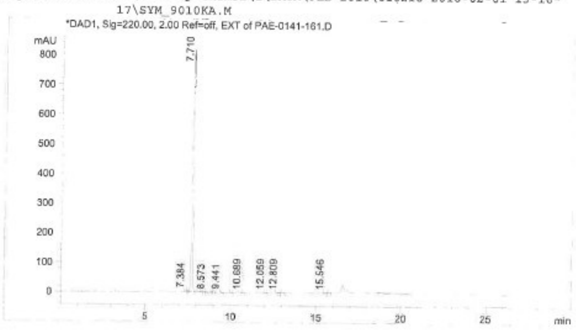

Signal 1: DAD1, Sig=220.00, 2.00 Ref=off, EXT

Peak #	RT [min]	Name	Area	Area %
1	7.384		16.7	0.361
2	7.449		22.5	0.488
3	7.710		4422.0	95.837
4	8.303		12.4	0.269
5	8.433		5.8	0.126
6	8.573		1.8	0.040
7	8.889		2.7	0.059
8	9.022		39.9	0.866
9	9.441		4.6	0.100
10	10.091		2.4	0.052
11	10.689		1.9	0.042
12	12.059		7.5	0.162
13	12.740		18.1	0.392
14	12.809		2.7	0.059
15	13.015		28.2	0.611
16	15.546		6.8	0.148
17	15.777		17.9	0.387

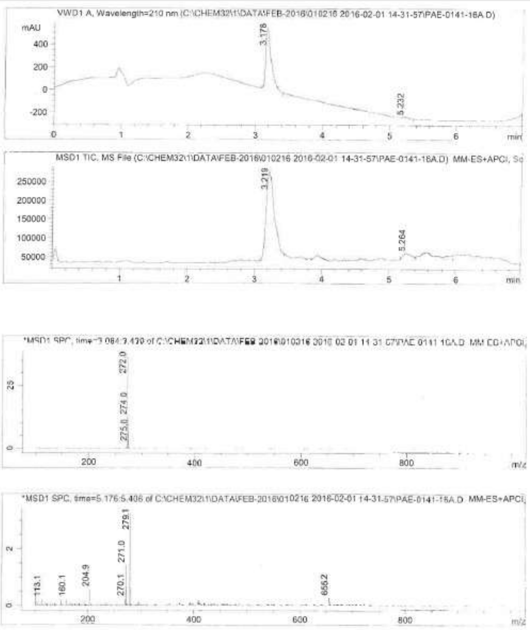

VWD1 A, Wavelength=210 nm

Peak #	Compound Name	Meas. Ret. T Min	Area	Area %
1		3.18	3334.5	96.45
2		5.23	122.8	3.55

Compound 20 (N^2-(5-methoxyisoquinolin-1-yl)pyrazine-2,6-diamine)

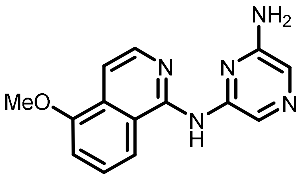

- ^{1}H NMR

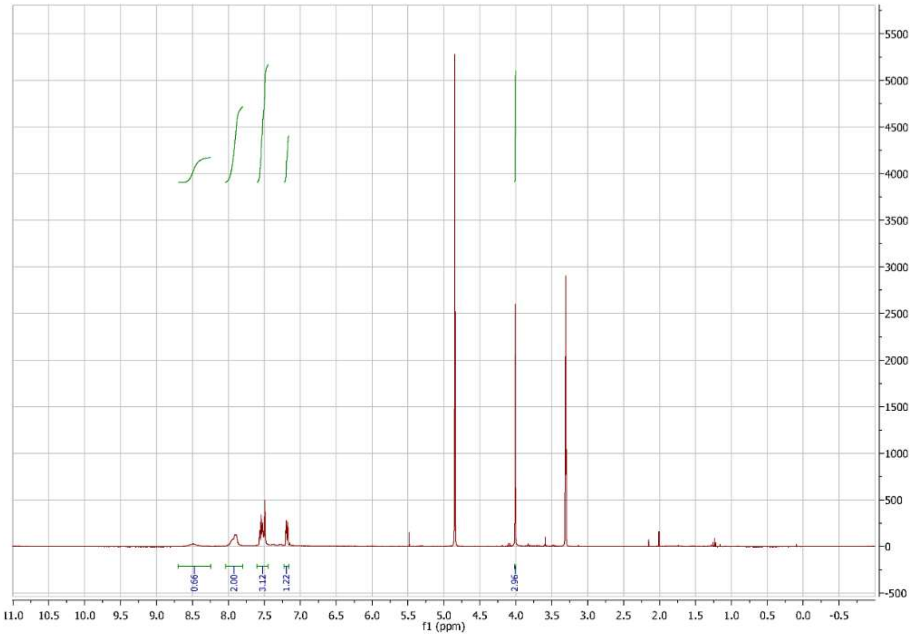

- HPLC-MS

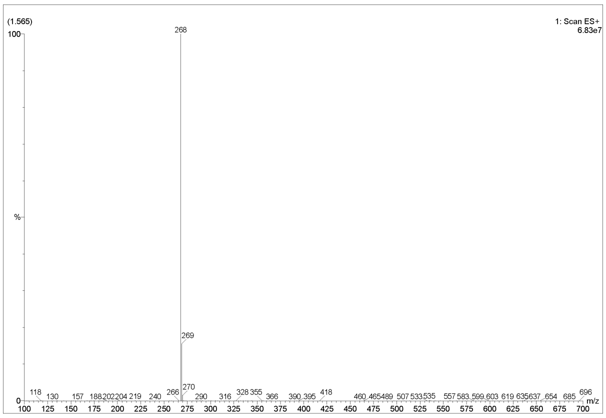

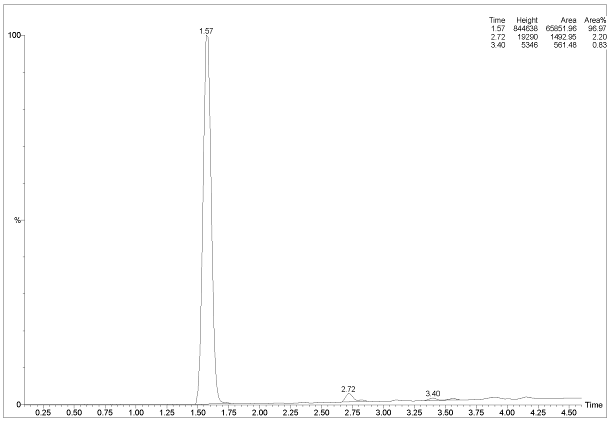

Compound 21 (1-((2-aminopyrimidin-5-yl)amino)isoquinoline-7-carbonitrile)

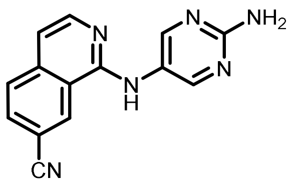

- ^{1}H NMR

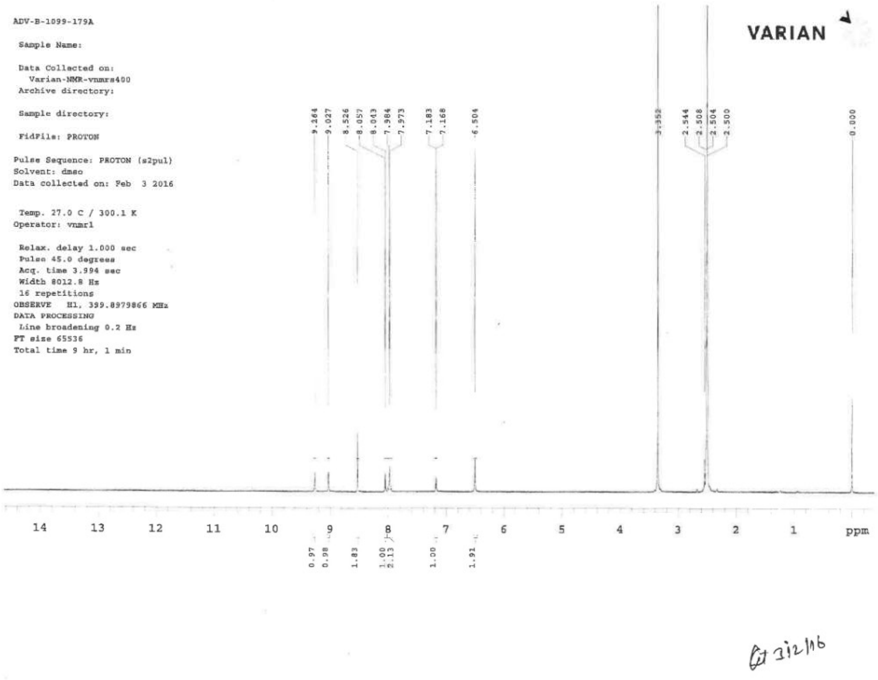

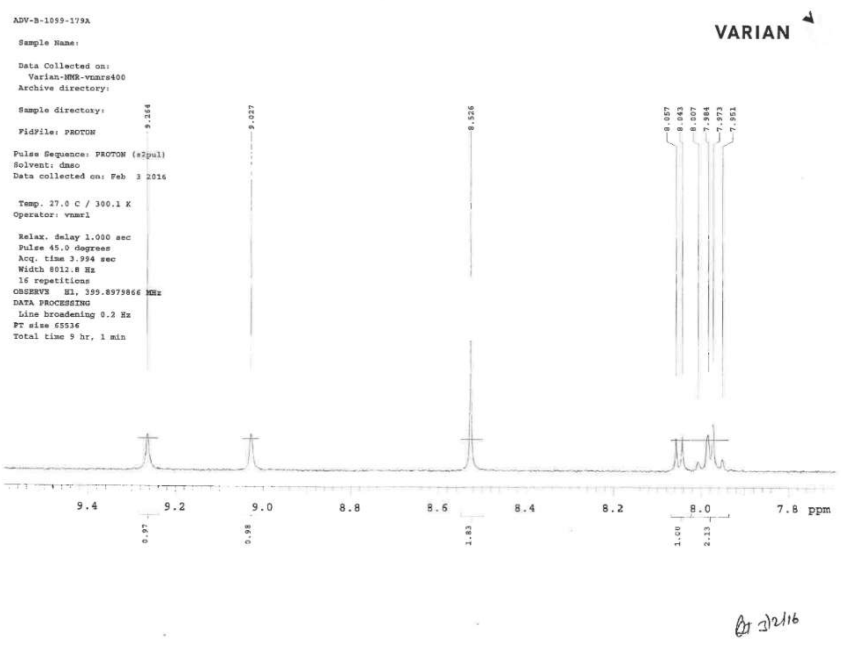

Compound 21 (1-((2-aminopyrimidin-5-yl)amino)isoquinoline-7-carbonitrile)

- HPLC-MS

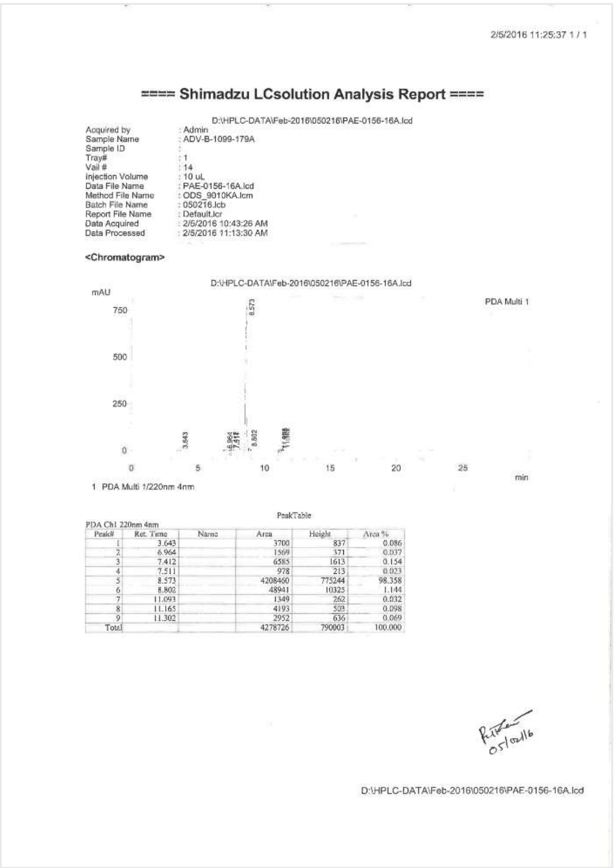

2/5/2016 11:25:37 1 / 1

==== Shimadzu LCsolution Analysis Report ====

D:\HPLC-DATA\Feb-2016\050216\PAE-0156-16A.lcd

Acquired by	: Admin
Sample Name	: ADV-B-1099-179A
Sample ID	:
Tray#	: 1
Vail #	: 14
Injection Volume	: 10 uL
Data File Name	: PAE-0156-16A.lcd
Method File Name	: ODS_9010KA.lcm
Batch File Name	: 050216.lcb
Report File Name	: Default.lcr
Data Acquired	: 2/5/2016 10:43:26 AM
Data Processed	: 2/5/2016 11:13:30 AM

<Chromatogram>

D:\HPLC-DATA\Feb-2016\050216\PAE-0156-16A.lcd

1 PDA Multi 1/220nm 4nm

PeakTable

PDA Ch1 220nm 4nm

Peak#	Ret. Time	Name	Area	Height	Area %
1	3.643		3700	837	0.086
2	6.964		1569	371	0.037
3	7.412		6585	1613	0.154
4	7.511		978	213	0.023
5	8.573		4208460	775244	98.358
6	8.802		48941	10325	1.144
7	11.093		1349	262	0.032
8	11.165		4193	503	0.098
9	11.302		2952	636	0.069
Total			4278726	790003	100.000

D:\HPLC-DATA\Feb-2016\050216\PAE-0156-16A.lcd

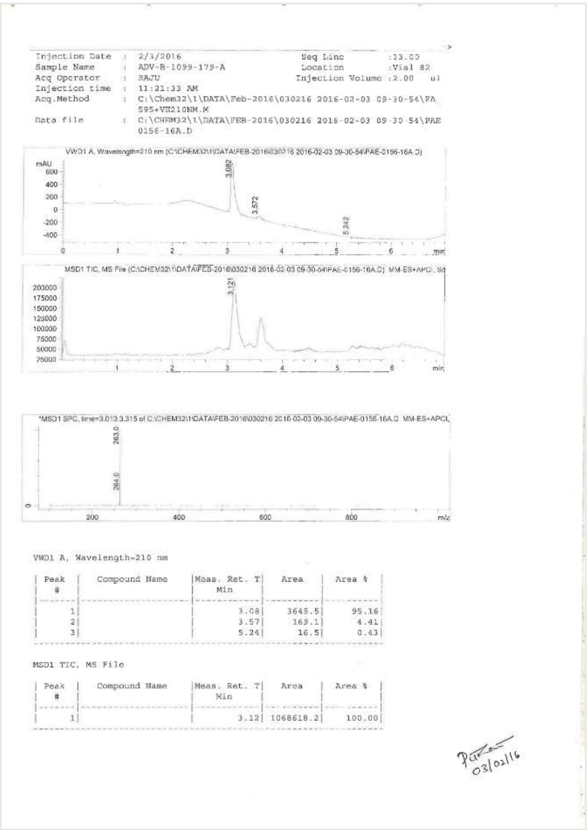

Injection Date	: 2/3/2016	Seq Line	:13.00
Sample Name	: ADV-B-1099-179-A	Location	:Vial 82
Acq Operator	: RAJU	Injection Volume	:2.00 ul
Injection time	: 11:21:33 AM		
Acq.Method	: C:\Chem32\1\DATA\Feb-2016\030216 2016-02-03 09-30-54\FA 595+VE210NM.M		
Data file	: C:\CHEM32\1\DATA\FEB-2016\030216 2016-02-03 09-30-54\PAE 0156-16A.D		

VWD1 A, Wavelength=210 nm

Peak #	Compound Name	Meas. Ret. T Min	Area	Area %
1		3.08	3645.5	95.16
2		3.57	169.1	4.41
3		5.24	16.5	0.43

MSD1 TIC, MS File

Peak #	Compound Name	Meas. Ret. T Min	Area	Area %
1		3.12	1068618.2	100.00

Compound 22 (1-((6-aminopyrazin-2-yl)amino)isoquinoline-7-carbonitrile)

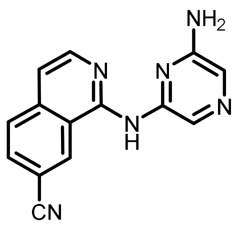

- ^{1}H NMR

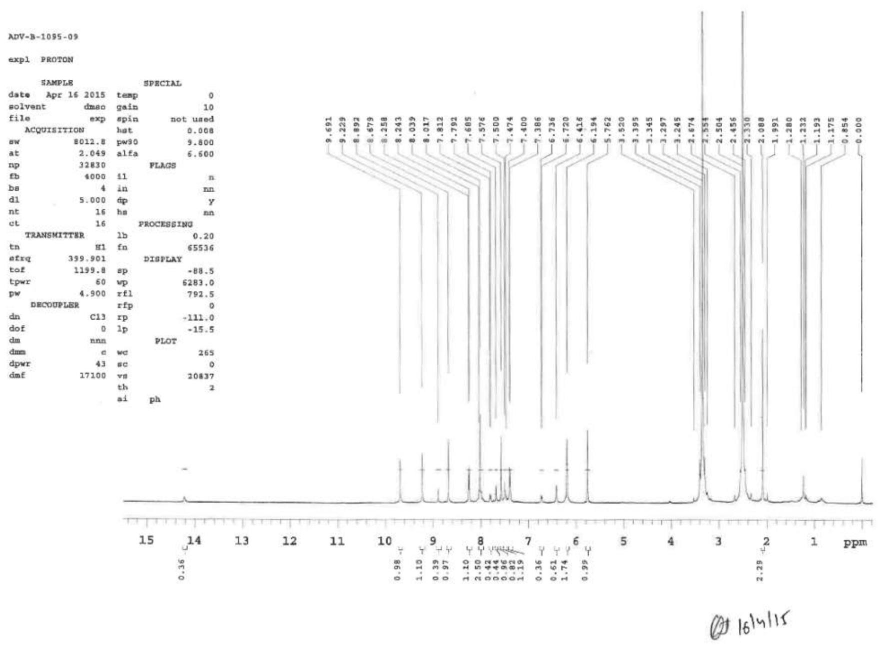

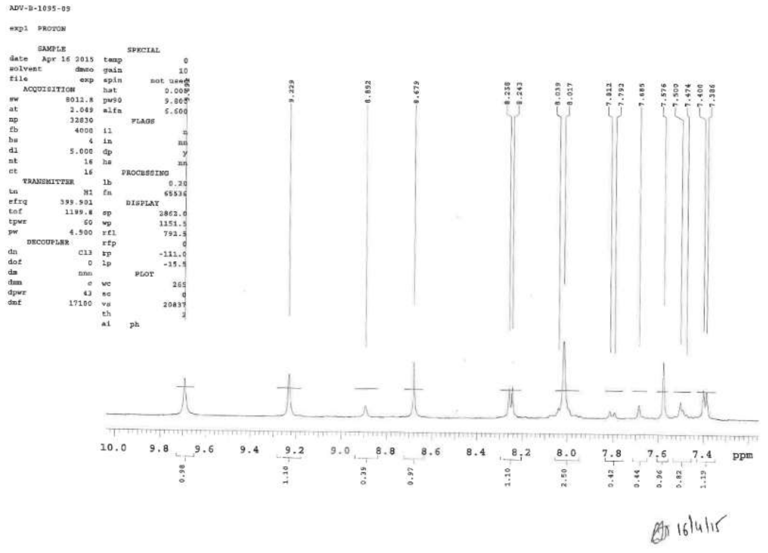

Compound 22 (1-((6-aminopyrazin-2-yl)amino)isoquinoline-7-carbonitrile)

- HPLC-MS

Injection Date : 4/16/2015 Seq Line :1.00
Sample Name : ADV-B-1095-09 Location :Vial 14
Acq Operator : Sai Injection Volume :10.00 ul
Injection time : 2:10:21 PM
Acq.Method : D:\data\APRIL 2015\160415 2015-04-16 14-08-07\AA_595+VE(210).M
Data file : D:\DATA\APRIL 2015\160415 2015-04-16 14-08-07\PAE-0692-15.D

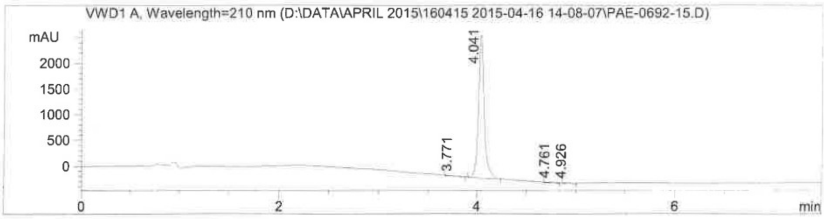

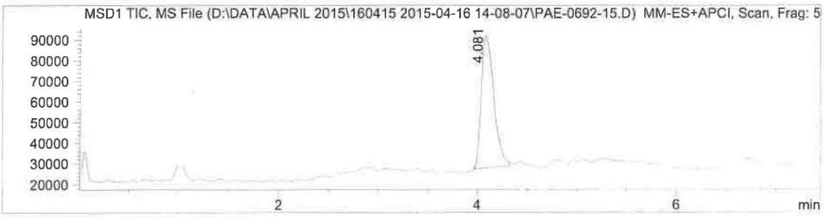

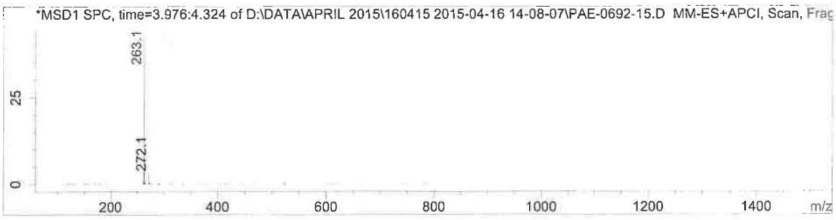

VWD1 A, Wavelength 210 nm

Peak #	Compound Name	Meas. Ret. T Min	Area	Area %
1		3.77	184.2	1.54
2		4.04	11478.3	95.77
3		4.76	141.4	1.18
4		4.93	181.2	1.51

MSD1 TIC, MS File

Peak #	Compound Name	Meas. Ret. T Min	Area	Area %
1		4.08	560001.9	100.00

Sai 16/4/16

Compound 23 (*N*[4]-(7-Chloroisoquinolin-1-yl)pyridine-2,4-diamine).

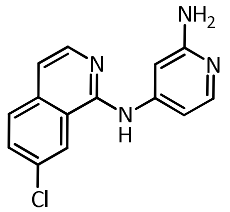

- ^{1}H NMR

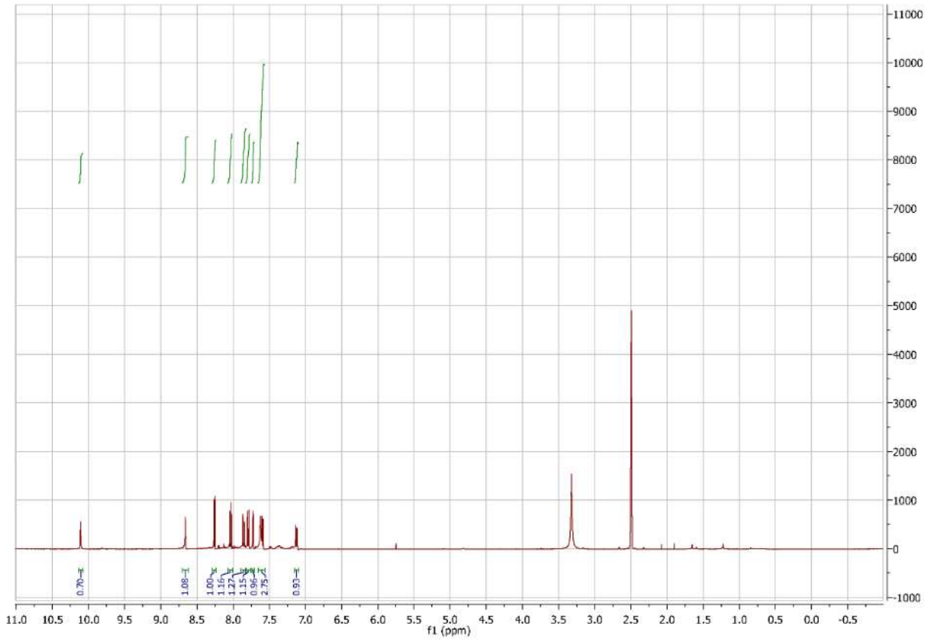

- HPLC-MS

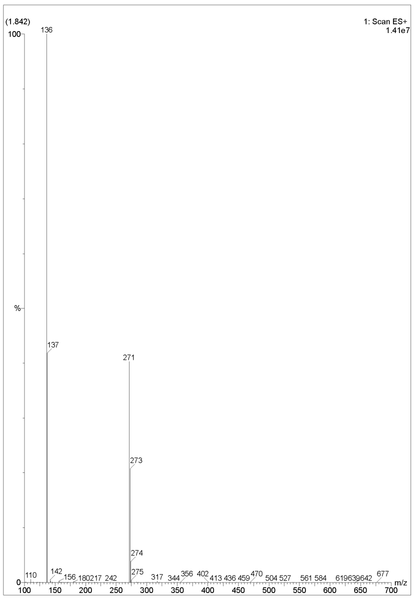

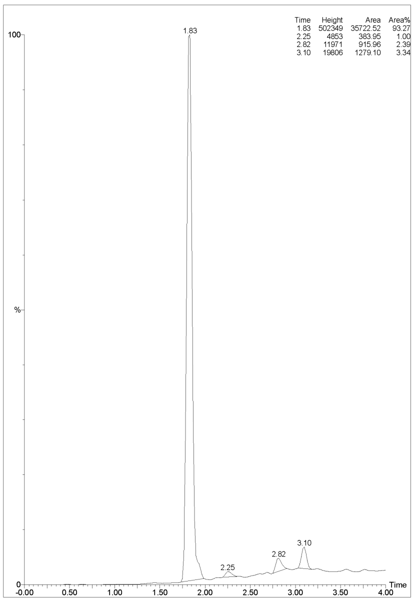

Supplement: Supplementary file 1 [file ijms-27-03631-s001.zip › File S2.pdf]
