# Supplementary material for: Development of Small-Molecule Allosteric Modulators of Beta-Galactosidase (β-Gal) for the Treatment of GM1 Gangliosidosis and Morquio B
Source: Int J Mol Sci. 2026 Apr 18;27(8):3631. doi: 10.3390/ijms27083631 (PMC13115887; doi:10.3390/ijms27083631)
Supplement: Supplementary file 1 [file ijms-27-03631-s001.zip › Table S1.pdf]

Table S1. Thermal Shift Assay Results for Virtual Hits (144 compounds). This table presents the thermal shift (ΔTm) observed for wild-type recombinant human β-Galactosidase (rhGLB1, Novoprotein) in the presence and absence of 144 virtually screened compounds. Differential scanning fluorimetry (DSF) screening was performed in triplicate (n=1) at pH 7.4 with a compound concentration of 100 μM or 30 μM, with select hits undergoing a second independent triplicate experiment (n=2). The significance of melting temperature (Tm) shifts was evaluated using two criteria: an absolute ΔTm shift ≥ 0.5 °C (instrumental criterion) and an absolute ΔTm standard deviation ≤ 0.2 °C (statistical criterion).

Compound (Validated hit)	Concentration (μM)	ΔTm (ºC)	S.D.	n
1	100	-0,25	0,20	1
2	100	-0,52	0,00	1
3	100	-0,51	0,00	1
4	100	-0,78	0,20	1
5	100	-1,07	0,20	1
6	100	0,25	0,20	1
7	100	-0,78	0,20	1
8	100	-0,52	0,00	1
9	100	-0,25	0,20	1
10	100	-0,76	0,20	1
11	100	-0,25	0,20	1
12	100	-0,51	0,00	1
13	100	-0,25	0,20	1
14	100	-0,25	0,20	1
15	100	-0,76	0,20	1
16	100	-0,27	0,20	1
17	100	0,25	0,20	1
18	100	0,23	0,20	1
19	100	-0,25	0,20	1
20	100	-0,52	0,20	1
21	100	0,00	0,20	1
22	100	-0,25	0,20	1
23	100	-0,04	0,20	1
24	100	-0,76	0,20	1
25	100	-0,78	0,20	1
26	100	-0,25	0,20	1
27	100	0,00	0,00	1
28	100	0,25	0,20	1
29	100	-0,25	0,20	1
30	100	0,25	0,20	1
31	100	-0,51	0,00	1
32	100	0,00	0,00	1
33	100	0,00	0,00	1

Compound (Validated hit)	Concentration (μM)	ΔTm (ºC)	S.D.	n
34	100	0,00	0,00	1
35	100	0,00	0,00	1
36 **(1)**	100	**0,51**	0,05	2
37	100	-0,25	0,20	1
38	100	0,00	0,00	1
39	100	0,00	0,00	1
40	100	0,00	0,00	1
41	100	-0,76	0,20	1
42	100	0,00	0,00	1
43	100	0,25	0,20	1
44	100	0,00	0,00	1
45	100	0,00	0,00	1
46	100	0,00	0,00	1
47	100	0,00	0,00	1
48	100	0,25	0,20	1
49	100	0,00	0,00	1
50	100	0,00	0,00	1
51	100	0,25	0,20	1
52	100	-0,25	0,20	1
53 **(2)**	100	**0,51**	0,10	2
54	100	0,00	0,20	1
55 **(3)**	100	**1,27**	0,12	2
56	100	0,00	0,00	1
57	100	-0,34	0,00	1
58	100	-0,59	0,20	1
59	100	-0,34	0,00	1
60	100	-0,34	0,00	1
61	100	-0,34	0,00	1
62	100	-15,37	0,00	1
63	100	-0,34	0,00	1
64	100	-0,34	0,00	1
65	100	-0,59	0,20	1
66	100	-0,34	0,00	1
67	100	-0,59	0,20	1
68	100	-0,34	0,00	1
69	100	-0,08	0,20	1
70	100	-0,59	0,20	1
71	100	-0,34	0,00	1
72	100	-0,59	0,20	1
73	100	-0,85	0,00	1
74	100	-0,85	0,00	1
75	100	-0,59	0,20	1
76	100	-0,34	0,00	1
77	100	-0,85	0,00	1

Compound (Validated hit)	Concentration (μM)	ΔTm (ºC)	S.D.	n
78	100	-0,34	0,00	1
79	100	-0,64	0,03	1
80	100	-0,51	0,04	1
81	100	-0,67	0,14	1
82	100	-0,47	0,03	1
83	100	-0,49	0,02	1
84	100	-0,58	0,04	1
85	100	-0,29	0,00	1
86	100	-0,35	0,09	1
87	100	-0,33	0,07	1
88	100	-0,51	0,20	1
89	100	-0,46	0,02	1
90	100	-0,55	0,22	1
91	100	-0,32	0,06	1
92	100	0,02	0,09	1
93	100	-0,45	0,04	1
94	100	-1,72	0,11	1
95	100	-0,37	0,05	1
96	100	-0,33	0,20	1
97	100	-0,21	0,05	1
98	100	-0,54	0,11	1
36 **(1)**	30	-0,11	0,15	2
53 **(2)**	30	-0,08	0,18	2
55 **(3)**	30	**1,08**	0,20	2
99	30	0,03	0,20	1
100	30	0,14	0,20	2
101	30	0,11	0,10	2
102	30	0,28	0,16	2
103 **(4)**	30	**0,98**	0,20	2
104	30	-0,17	0,20	1
105	30	0,25	0,03	1
106	30	0,05	0,21	1
107 **(5)**	30	**2,18**	0,14	2
108 **(6)**	30	**0,58**	0,10	2
109	30	0,16	0,20	2
110	30	-0,66	0,12	2
111	30	0,11	0,14	1
112	30	0,28	0,12	1
113	30	0,23	0,03	1
114	30	0,08	0,07	1
115	30	-0,02	0,04	1
116	30	-0,04	0,08	1
117	30	0,06	0,06	1
118	30	0,11	0,10	1

Compound (Validated hit)	Concentration (μM)	ΔTm (ºC)	S.D.	n
119	30	-0,86	0,12	1
120	30	0,07	0,03	1
121	30	0,22	0,02	1
122 **(7)**	30	**0,55**	0,20	2
123	30	0,14	0,08	1
124	30	0,07	0,09	1
125	30	0,26	0,05	1
126	30	-0,01	0,08	1
127 **(8)**	30	**0,88**	0,19	2
128	30	0,26	0,15	1
129	30	0,28	0,27	1
130	30	0,16	0,21	1
131	30	-0,89	0,10	1
132 **(9)**	30	**1,10**	0,07	2
133	30	-0,06	0,13	1
134	30	0,27	0,09	1
135	30	-0,07	0,12	1
136	30	0,23	0,09	1
137	30	-0,02	0,02	1
138	30	0,11	0,17	1
139	30	0,06	0,20	2
140	30	0,07	0,10	1
141	30	0,23	0,03	1
142	30	0,31	0,16	2
143	30	0,05	0,17	2
144	30	-0,02	0,15	2
